# Dynamic supervision of counterfeit products based on blockchain technology: A differential game on goodwill accumulation

**DOI:** 10.1371/journal.pone.0293346

**Published:** 2023-10-23

**Authors:** Zhongmiao Sun, Qi Xu, Jinrong Liu

**Affiliations:** 1 School of Economics & Management, Shanghai Maritime University, Shanghai, China; 2 Glorious Sun School of Business and Management, Donghua University, Shanghai, China; 3 School of Business and Management, Shanghai Urban Construction Vocational College, Shanghai, China; National Textile University, PAKISTAN

## Abstract

Counterfeiting is a serious problem in many industries, and the annual profit and tax losses caused by counterfeit products in China have reached over 60 billion yuan. The focus of this paper is to study the effect of blockchain technology in terms of supervising counterfeit products as well as product quality and service decisions under blockchain from a dynamic optimization perspective. We consider the role of blockchain when disclosing product quality information and develop a dynamic optimization model before and after blockchain adoption using differential game theory. Meanwhile, we solve the model using the Hamilton-Jacobi-Bellman (HJB) equation and backward induction and conduct parametric, comparative, and numerical analyses of the equilibrium solutions. The main findings are as follows: First, we find that in the absence of blockchain, counterfeit products can reduce brand goodwill and decrease the quality and service level of genuine products; however, supervising counterfeit product sales through blockchain can improve this problem under the critical conditions determined in this work. Second, in the direct sales mode, we find that if the unit operating cost and fixed cost of blockchain are small, the brand owner’s adoption of blockchain can not only combat the counterfeiter’s profit but also increase its own profit. Third, in the wholesale sales mode, we find that the best conditions in which a brand owner can establish blockchain are only related to the related costs of blockchain, while retailers also need to increase the selling price of genuine products when establishing blockchain. Fourth, we find that supply chain performance and blockchain supervisory effects are sensitive to key parameters through numerical analysis in a quantitative form. These findings have important implications for genuine enterprises in terms of determining the conditions for establishing blockchain to combat counterfeiting and for optimizing product quality and service decisions when using blockchain technology.

## 1 Introduction

### 1.1 Background and motivation

The problem of counterfeit products is an important issue that hinders the healthy development of China’s market economy. In many industries, counterfeit products are common, and they not only damage the interests of consumers but also seriously affect the brand goodwill for genuine products and lead to the loss of market share by genuine enterprises [1–3]. It is estimated that in the clothing, cosmetics, handbags and watch industries, the losses caused by counterfeits are $98 billion per year, while the economic impact caused by counterfeit electronic components is approximately $169 billion [4]. By imitating the design of existing products, counterfeiters can quickly forge fake products, which violate the legitimate rights and interests of genuine enterprises and endanger the survival and development of enterprises. In the past, genuine enterprises have mainly used traditional technologies (such as bar codes and RFID tags) to crack down on counterfeiters, but the data used in these technologies cannot be encrypted and verified. Counterfeiters can copy and print genuine products’ quality information on imitations, which causes the products purchased by consumers to still likely be counterfeit [5].

Blockchain technology based on distributed ledgers can help genuine enterprises provide consumers with product authenticity verification to supervise the sale of counterfeit products. Blockchain has the characteristics of decentralization, openness, and transparency, and it is tamper-proof. It can provide consumers with product traceability services in the supply chain; that is, consumers can be authorized to track and access the information of raw material selection, production and processing, transportation and circulation, and sales of products in the blockchain system. In practice, Walmart has implemented blockchain to provide consumers with traceability services for more than 100 commodities covering more than 10 categories, such as packaged fresh meat, vegetables, seafood and private brands; LVMH, the Richemont Group and Prada jointly launched the global luxury blockchain to help consumers check the authenticity of products; and HP helps manufacturers better control the design and production of products by uploading and coding the intellectual property rights related to specific parts of printers into the blockchain. When parts are produced, the relevant information is updated in the blockchain, which fundamentally changes the counterfeiting behavior for these parts. However, although these large enterprises have taken the lead in implementing blockchain, according to Gartner data, only 3.3% of the companies have deployed blockchain in actual operation [6], mainly because the establishment and implementation of blockchain increases the cost. For example, to develop the blockchain, companies in the diamond industry need to pay a relatively large cost to purchase a laser machine to establish the digital fingerprint function. Therefore, weighing the costs and benefits of implementing blockchain has important practical significance for research on enterprises’ supervision of supply chain counterfeit products based on blockchain technology.

In addition, for brand owners in genuine sales channels, the brand goodwill for products is related to the good image of enterprises and consumers’ favorable impression of enterprises, which originates from an enterprise’s good reputation, reliable product quality, high-quality store service level and a favorable business position. Existing evidence shows that [7] brand goodwill is not static with changes in the market environment but is more dynamic and continuous [8,9]. Although the literature has considered using blockchain technology in the supply chain to combat counterfeit products (e.g., Shen et al., [1]; Pun et al., [4]; Danese et al., [10]; Haq and Muselemu, [11]), there are still some research gaps. Specifically, there is a research gap in terms of considering the impact of counterfeiting on the goodwill for genuine brands and exploring the conditions and effects of applying blockchain technology to supervise counterfeiting by genuine enterprises. Meanwhile, there is also a research gap in terms of exploring the optimal dynamic trajectories of supply chain operation strategy under blockchain technology by making decisions about the product quality and store service level from a dynamic optimization perspective using differential game theory. Therefore, in order to fill these gaps, this paper examines the problem of the adverse impact of counterfeit products on brand goodwill, considers the impact of blockchain technology on the symmetry of product quality information, uses differential game theory, introduces the time factor to describe brand goodwill, and constructs the state change equation for brand goodwill without blockchain and blockchain application. From the perspective of dynamic changes in brand goodwill, studying the conditions and effects of genuine enterprises’ application of blockchain in the supervision of counterfeit products has a strong practical demand in terms of improving the product quality and service level of the supply chain from production to sales and improving the brand image of genuine products.

### 1.2 Research questions

Based on the above realistic background regarding counterfeit products and blockchain applications as well as the motivation to fill the research gaps, some new and important issues have arisen in supply chain management under blockchain technology. Thus, we propose the following four research questions (RQs).

**RQ1**: From the perspective of dynamic optimization, based on the state changes of brand goodwill, how can equilibrium decisions be made between genuine enterprises and counterfeiters before and after blockchain application?
**RQ2**: How do some key parameters affect the optimal decision-making of chain members, expected discounted profits and the adoption of blockchain?
**RQ3**: What are the optimal dynamic trajectory change rules of brand goodwill, consumer demand for genuine and counterfeit products, and expected discounted profits of related enterprises under different blockchain adoption scenarios?
**RQ4**: How effective is blockchain technology in terms of supervising counterfeit products?

### 1.3 Research objectives

The research objectives (ROs) of this paper are as follows:

**RO1**: Establish a dynamic optimization model for blockchain supervision of counterfeit products using differential game theory.

**RO2**: Identify the optimal quality and service decisions for genuine enterprises under blockchain technology when combating the sale of counterfeit products in direct and wholesale sales modes.

**RO3**: Clarify the optimal dynamic trajectory change rules of the supply chain operation strategy under blockchain technology.

**RO4**: Uncover the conditions and effects of brand owners using blockchain technology to supervise counterfeit products.

### 1.4 Contributions

The main contributions of this research are threefold.

First, this work expands the research methodology for the dynamic optimization of product quality and service decisions for genuine enterprises under blockchain technology. We design brand goodwill under the influence of counterfeit products as a state equation and introduce the differential game method to the study of blockchain adoption in supply chain management.

Second, this work contributes to research perspectives on blockchain technology to combat counterfeit products in the supply chain. We consider both the genuine sales channel and the counterfeit product sales channel and construct dynamic decision models of the supply chain under blockchain technology from the perspective of brand goodwill accumulation.

Third, this work makes practical contributions to the research content. We provide the optimal quality and service dynamic optimization decisions of counterfeiters and genuine enterprises under different differential game situations as well as the optimal dynamic trajectories of consumer demand, brand goodwill and expected discounted profits. Through a systematic analysis of the model, we also propose the best conditions under which brand owners can supervise counterfeit products based on blockchain technology under different sales modes and finally conduct a numerical analysis of supply chain performance and supervisory effects, which have rarely been covered in previous studies.

To the best of our knowledge, this paper is the first to consider both brand goodwill changes and product authenticity verification under blockchain technology in both the counterfeit product sales channel and genuine sales channels with a direct or wholesale sales mode and the resulting product quality, store service level decisions and blockchain supervision of counterfeit products.

### 1.5 Paper organization and structure

To maintain and increase the consistency of this paper, we clarify the organization and structure of the remaining sections as follows: In Section 2, we present a review of the related literature on counterfeit products, blockchain adoption, and dynamic optimization in the supply chain and explain the research differences between this work and the literature. Subsequently, we follow the existing relevant studies and provide the symbol description and assumptions as well as the methodology of this paper in Section 3. Furthermore, in Sections 4 and 5, we construct supply chain dynamic optimization models before and after blockchain adoption in direct and wholesale sales modes, respectively, and obtain equilibrium solutions. Based on this work, in Section 6, we describe the conducted model analysis, including the parameter analysis, comparative analysis of the optimal decisions, optimal dynamic trajectory analysis of the supply chain operation strategy, and the effect of blockchain technology in terms of supervising counterfeit products. To enhance the generality and robustness of the conclusion, in Section 7, we describe the conducted numerical analysis, which includes the sensitivity of supply chain performance and the blockchain regulatory effect to key parameters. Finally, we conclude this paper and discuss the limitations as well as potential future research directions in Section 8. All proofs can be found in S1 Appendix.

The logical structure of the entire paper is as follows: Firstly, in Sections 1 and 2, we explain “Why do this paper” by clarifying the research motivation and reviewing the gaps in the existing literature. Secondly, in Sections 3–5, we explain “how it is done” by introducing the methodology and constructing and solving the model. Finally, in Sections 6–8, we explain “what is done” through equilibrium analysis and summarization.

## 2 Literature review

The main studies related to this paper can be divided into three categories: counterfeit products in the supply chain, supply chain management under blockchain technology, and the dynamic decision-making of the supply chain based on brand goodwill.

### 2.1 Counterfeit products in the supply chain

In recent years, many scholars have considered both non-deceptive and deceptive sales of counterfeit products in the supply chain [4]. Regarding research on the sales of non-deceptive counterfeit products, Gao et al. [10] analyzed how brand makers’ decision to exit the market and the counterfeiters’ decision to enter the market affected consumer welfare and social surplus. They found that the entry of counterfeit products does not always increase consumer surplus and social welfare. Subsequently, Gao et al. [12] determined the conditions for the successful entry of counterfeiters into the market and found that the potential entry of counterfeiters created implicit pressure for brand makers to reduce their sales prices. Pun and DeYong [13] considered that consumers are strategic for the first time in the competition between brands and counterfeiters and found that when consumers prefer to buy products immediately, it may be disadvantageous to brands. Ghamat et al. [14] considered that suppliers and third-party manufacturers can imitate the products of brand manufacturers and sell them to the market. They found that signing an intellectual property agreement between manufacturers and their suppliers may have more serious consequences, even if the intellectual property agreement is cost-free and fully enforceable. Recently, Bian et al. [15] analyzed the strategic impact of vertical integration on non-deceptive counterfeiting behavior, and they found that integration has lower requirements for improving the quality and efficiency of brand products and can serve as an anti-counterfeiting strategy to help brand enterprises curb non-deceptive counterfeiting behavior. The above literature assumes that consumers can clearly identify the authenticity of products before making a purchase decision, but consumers may still choose to buy fake products for economic reasons.

In addition, Islam and Muhammad [16] were motivated by the fact that counterfeit drugs have become a threat to global public health and analyzed all relevant research on preventing or reducing counterfeit drugs through digital interventions. They found that over time, counterfeit drugs have become a key issue in research and investigation. Ziavrou reviewed [17] the trends in counterfeit drugs and drug trafficking, the impact of their use on health, and the measures and actions taken to limit the spread of counterfeit drugs and drugs before and during the COVID-19 pandemic. Ryu et al. [18] explored how linguistic and visual aspects of anti-counterfeiting advertisements influence the purchase of counterfeit luxury brands, and they found that anti-counterfeiting messages influenced participants’ purchase intentions through social adjustment and the perception of value-expression benefits. Pittiglio [19] examined the intra- and interindustry impact of counterfeiting on the survival of Italian firms. They found that the probability that a genuine firm exits the market increases when the impact flows from the “counterfeiting” industry to its upstream suppliers of genuine goods. Houck [20] noted that counterfeit goods not only pose a threat to the economy and consumer goods but also pose a threat to the end users of other types of goods, and counterfeit products not only threaten the global economy but also national security. Zhou et al. [21] studied the impact of anti-counterfeiting on retail platforms and the motivation of platforms and manufacturers to invest in anti-counterfeiting technology through game theory models. They found that when the production valuation is low, if the proportion of counterfeit goods is sufficiently low, the platform’s anti-counterfeiting return is negative.

Different from these studies, this paper considers the sale of deceptive counterfeit products, and consumers cannot distinguish the authenticity of products when purchasing products. Although some scholars, such as Qian [22], Cho et al. [23] and Gao [24], have proposed traditional solutions to price signals and quality improvement strategies for the sale of fraudulent counterfeit products, this work does not completely eliminate the possibility of counterfeiters cheating consumers.

### 2.2 Supply chain management under blockchain technology

Supply chain management based on blockchain technology is currently a popular research topic in academia. Some scholars have discussed the application value and possible problems of blockchain in the supply chain through investigation and research [25–27]. For example, blockchain can help supply chain management in terms of network security and protection, transparency and accountability, traceability and fraud prevention, and smart contracts [28,29]. Bhushan et al. [30] noted that blockchain may also lead to user privacy disclosure because the data on the public chain may be stolen. In addition, Nazam et al. [31] evaluated blockchain technology-related barriers by developing an integrated model in the textile supply chain. They suggested that top-level management should pay more attention to and encourage bottom-level management to adopt technological and system-related practices and that technological transformation in the textile and apparel industry would be complicated by a number of constraints. Danese et al. [10] analyzed the design of blockchain systems in terms of effectively preventing forgery based on situational crime prevention and found that the expected level of upstream and downstream counterfeit protection that brand owners intend to ensure to customers through blockchain is a key driving factor to consider in the blockchain system design. Haq and Muselemu [11] elaborated on how to apply blockchain technology to the pharmaceutical supply chain, increasing traceability, visibility, and security for the pharmaceutical supply system. Moreover, in the health care industry, Tan et al. [32] proposed a searchable encryption scheme based on blockchain for storing and updating electronic health records. Saeed et al. [33] systematically reviewed the research trends of blockchain technology in the health care field and noted that future research can support universally implemented blockchain technology for health diagnosis and improve the remote monitoring of patients’ health care processes or the integrity of data in emergency situations.

Some scholars have discussed supply chain decision-making under blockchain technology by building mathematical models. For example, Choi [34] considered blockchain in terms of disclosing product quality information for luxury supply chains. Subsequently, Sun et al. [35] further considered the value of blockchain in terms of identifying product authenticity and sharing demand information and studied pricing and product quality decision-making in a two-level supply chain. Liu et al. [36] considered that blockchain could be used to track the source and destination of imported fresh food and explored the optimal pricing before and after blockchain application and the conditions for blockchain implementation. Guo et al. [37] studied a two-period price competition model for second-hand product platforms with and without blockchain at different levels of supply and demand and found that the Matthew effect of whether to use blockchain is evident, with pros and cons accumulating over time. Recently, Xu et al. [38] considered that manufacturers can use blockchain technology to improve demand forecasting and studied optimal production, pricing, delivery time decision-making, and coordination issues in the supply chain. They found that cross-channel effects and blockchain have a significant impact on optimal decision-making and coordination outcomes, especially when the adoption of blockchain helps promote coordination between manufacturers and platforms. Liu et al. [39] explored the application of blockchain technology in a supply chain consisting of two competitive manufacturers and a retailer and found the equilibrium conditions between the firms. Zhong et al. [40] studied the value-added effect of manufacturers using blockchain technology and found that when manufacturers sell products through two channels, they are always willing to use blockchain technology to disclose product information.

In addition, Xu et al. [41] investigated the pricing problem for blockchain service providers and proposed an efficient algorithm for optimal decision-making regarding the conservative pricing of blockchain services for the service provider. Pu et al. [42] first explored a game-theoretic approach to optimal blockchain adoption time using ship operators as an example and developed an algorithm to obtain a numerical solution for the optimal adoption time of ship operators. Niu et al. [6] discussed the necessity for multinational companies to implement blockchain to provide consumers with product quality certification, and Zhang et al. [43] studied the bidirectional effects of the gray market and blockchain adoption in global supply chains and explored the equilibrium strategies of manufacturers and gray marketers under different power structures by constructing Stackelberg games. Relevant studies include Di and Varriale [28], Emel [44] and Choi et al. [45]. However, these documents do not regard counterfeiters in the supply chain as decision-makers. Recently, Pun et al. [4] considered the existence of a manufacturer and a counterfeiter in the market and tested the effectiveness of blockchain in terms of combating counterfeiting for the first time. Shen et al. [1] divided consumers into novice consumers and expert consumers and studied how blockchain technology platforms can crack down on imitators in the supply chain. There is an abundance of related research that has attracted the interest of many scholars [46–51].

However, Shen et al. [1] and Pun et al. [4] did not consider the impact of counterfeit products on the goodwill for genuine brands, nor did they study product quality and service decisions under blockchain technology from a dynamic perspective.

### 2.3 Dynamic decision-making for the supply chain based on brand goodwill

The research related to this paper also includes dynamic decision-making for the supply chain based on brand goodwill. Nerlove and Arrow [7] first defined the brand goodwill of products in the market as the state variable of time and proposed the classic Nerlove-Arrow dynamic model. On this basis, Krishnamoorthy et al. [52] analyzed the dynamic advertising and pricing decisions of durable products in a duopoly. Ma et al. [8] and Ma and Hu [9] incorporated the quality reference utility of consumers into the state equation of brand goodwill and studied the dynamic operation strategies of O2O supply chains and closed-loop supply chains; subsequently, Ma et al. [53] further investigated the optimal combination of blockchain in terms of recycling old products and sales forms in a closed-loop supply chain composed of a manufacturer and an online platform, selecting online platforms to achieve triple sustainability in the economy, environment, and society. In addition, Guan et al. [54] studied channel coordination under Nash bargaining fairness concerns from the perspective of a differential game of goodwill accumulation. Song et al. [55] studied dynamic innovation and pricing decisions in a two-stage supply chain and found that the reduction of the unit cost of innovation efforts would encourage enterprises to improve their brand goodwill. However, the above research does not introduce brand goodwill into the dynamic decision-making model of supply chains under blockchain technology.

Differential game theory is an effective method for solving dynamic optimization problems. Liu et al. [56] constructed an investment model for precooling energy and carbon emission reduction technologies in a fresh produce cold chain based on a differential game approach, and they found that reasonable investment in precooling and carbon emission reduction technologies by suppliers and retailers is very important in terms of maintaining the freshness of products and improving the overall profit of the supply chain. Zhang and Yu [57] conducted a differential game analysis of altruism model selection and coordination in a low-carbon closed-loop supply chain under compound government subsidies and found that when the manufacturer’s altruistic intensity is maintained at a reasonable level, the manufacturer’s altruistic behavior, as the dominant party, can promote emission reduction and recycling activities. Wang et al. [58] investigated the carbon reduction strategies of the construction supply chain under government subsidies using differential game methods and found that the total profit of the construction supply chain under decentralized decision-making reaches the level of centralized decision-making after the introduction of a cost-sharing contract. Meng et al. [59] considered different environmental regulations and explored emission reduction decisions between ports and shipping companies based on differential game theory; they found that government incentives for ports and shipping companies can improve both parties’ emission reduction efforts. Recently, Tao et al. [60] investigated the bilinear differential game problem of competitive advertisements with random perturbations and sudden shocks and proved the convergence of time-varying approximation sequences. Wang and Yan [61] explored the Stackelberg differential game for multi-follower stochastic systems based on the Pareto principle and obtained the feedback form for the Stackelberg equilibrium based on Pareto.

Following previous studies, we know that the role of differential game theory in solving dynamic optimization models lies mainly in the study of the decision-making process of two or more players to achieve their respective optimal goals when their control effects are simultaneously applied to a moving system described by differential equations. Thus, this paper considers the impact of counterfeit products on the goodwill of genuine products, makes dynamic optimization decisions about product quality and service using a differential game between counterfeiter and genuine enterprises, and explores the supervision of supply chain counterfeit products based on blockchain technology from the perspective of dynamic changes in brand goodwill, which is underrepresented in existing studies.

### 2.4 Research gap

To illustrate the differences between the literature review and this study and highlight the contributions of this paper, some representative articles are summarized and compared in Table 1. This paper fills the research gap in the literature regarding blockchain adoption and dynamic optimization in supply chain management. Specifically, first, although much of the literature focuses on blockchain adoption in the supply chain, most studies ignore the impact of counterfeit products on genuine brand goodwill, and fewer studies examine the dynamic optimization decisions regarding product quality and store service level under blockchain technology. We thus fill the research gap in this area. Second, following the existing dynamic optimization studies based on differential game theory, this paper fills this research gap by exploring the conditions under which brand owners should adopt blockchain technology to combat counterfeiters under direct and wholesale sales modes as well as the optimal dynamic trajectories of blockchain-driven supply chain operation strategy.

**Table 1:** Summary of the existing related literature.

Authors	Blockchain adoption	Brand goodwill accumulation	Supply chain structures		Modeling & decision-making		
			Genuine and counterfeit product channels	Direct sales or wholesale sales mode	Dynamic model based on differential games	Product quality decision	Store service level decisions
Shen et al. [1]	√		√	√		√	
Pun et al. [4]	√		√			√	
Shen et al. [5]	√					√	
Nerlove and Arrow [7]		√			√	√	
Ma et al. [8]		√		√	√	√	√
Ma and Hu [9]	√	√			√		
Danese et al. [10]	√		√			√	
Pun and DeYong [13]			√			√	
Zhou et al. [21]	√		√	√			
Choi [34]	√			√			
Sun et al. [35]	√					√	
Li et al. [48]	√	√			√		
Guan et al. [54]		√			√	√	
Song et al. [55]				√	√	√	
This paper	√	√	√	√	√	√	√

## 3 Symbol descriptions and assumptions

### 3.1 Symbol descriptions

First, the model symbols and descriptions in this paper are provided in Table 2.

**Table 2:** Model symbols and descriptions.

Symbol	Description
*t*	Time, *t*∈[0,+ ∞)
*e*	Potential market demand for branded products
*a*	Proportion of potential market demand for counterfeit products
*p*	Sales price of the product
*w*	Wholesale price of products
*ϕ*	Probability that consumers think the product is genuine without blockchain
*α*	Influence factor of product quality on brand goodwill
*δ*	Influence factor of service level on brand goodwill
*σ*	Natural decline rate of brand goodwill
*ρ*	Discount rate
*k*	Service cost coefficient
*η*	Product quality cost coefficient
*λ*	Impact of counterfeit products on brand goodwill
*θ*	Influence factor of brand goodwill on consumer demand
*β*	Influence factor of product quality on consumer demand
*γ*	Influence factors of service level on consumer demand
*u*	Quality reference coefficient of consumers
*c*	Unit operation cost of blockchain
*F*	Fixed cost of blockchain
*G*_0_	Initial value of brand goodwill
*G*	Brand goodwill
*d*_*f*_	Sales quantity of counterfeit products
*d*_*a*_	Sales quantity of genuine products
*q*_*f*_	Quality level of counterfeit products (decision variable)
*q*_*a*_	Quality level of genuine products (decision variable)
*s*_*a*_	Store service level of authentic sales (decision variable)
*Q*	Product quality perceived by consumers, *Q* = (1-*ϕ*) · *q*_*f*_ + *ϕ* · *q*_*a*_
*π*_*f*_	Total expected profit of the counterfeiter
*π*_*b*_	Total expected profit of the brand owner
*π*_*r*_	Total expected profit of the retailer
*V*_*f*_	Counterfeiter’s expected discounted profit
*V*_*b*_	Brand owner’s expected discounted profit
*V*_*r*_	Retailer’s expected discounted profit

### 3.2 Model assumptions

Considering that there are fake and inferior products in the market, suppose there is a brand owner in the supply chain who sells genuine products and a merchant who sells counterfeit products. This counterfeiter sells its counterfeit products to consumers by imitating the genuine product and passing it off as genuine. Compared with the genuine product, the counterfeit product has the following characteristics [1]: (1) The counterfeit product is a brand product of the counterfeiter that is highly similar to the genuine product but has differences in material and workmanship; (2) the merchants of counterfeit products are followers of the market; (3) the quality of counterfeit products is lower than that of genuine products; and (4) it is difficult for consumers to identify counterfeit products.

**Fig 1:**
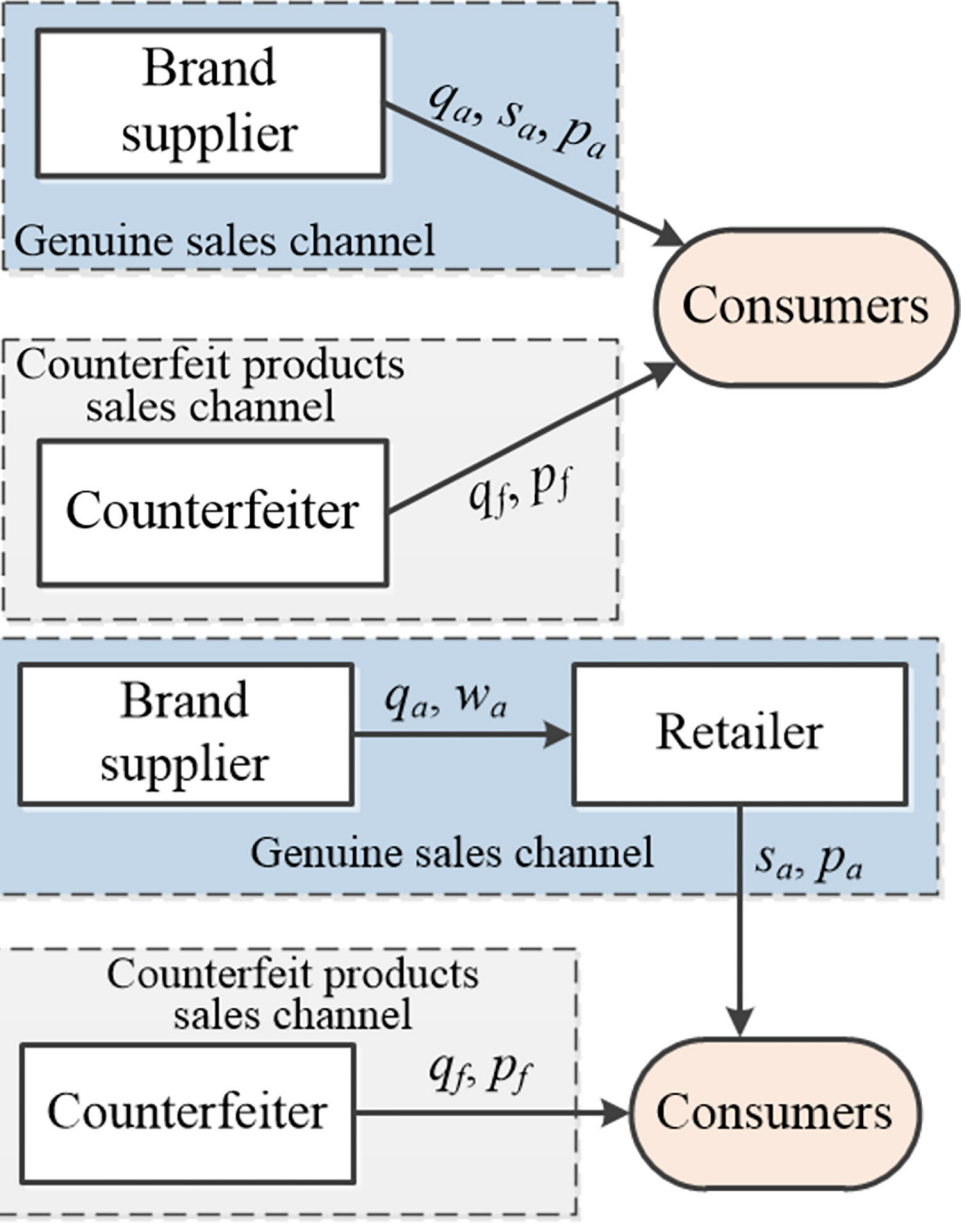
Supply chain structures.

As shown in Fig 1, in the supply chain with counterfeiters, two sales modes of brand owners are considered: one is the direct sales mode, as shown in Fig 1(A), and the other is the wholesale sales mode, as shown in Fig 1(B). In the direct sales mode, the brand owner sells genuine products of quality *q*_*a*_ and price *p*_*a*_ directly to consumers through its own offline stores at service level *s*_*a*_, while the counterfeiter sells counterfeit products of quality *q*_*f*_ and price *p*_*f*_ to consumers, for example, online. In the wholesale sales mode, the brand owner first wholesales genuine products of quality *q*_*a*_ and price *w*_*a*_ to an offline retailer who then sells them to consumers at price *p*_*a*_ and shop service level *s*_*a*_, while the counterfeiter also sells counterfeit products of quality *q*_*f*_ and price *p*_*f*_ to consumers. Generally, the sale of counterfeit products is fraudulent sales behavior, which does not involve fixed physical stores and involves mostly online sales.

For ease of illustration, subscript *a* is used to indicate the sales channel of genuine products, while subscript *f* is used to indicate the sales channel of counterfeit products. Referring to the existing relevant research and combining it with the realistic background, the main assumptions of this paper are as follows:

(1) In the absence of blockchain, consumers cannot recognize the authenticity of the product, and there is information asymmetry between the perceived product quality and the actual product quality. For this reason, it is assumed that the product quality perceived by consumers when purchasing the brand product in the market is *Q*_*a*_ = *Q*_*f*_ = *ϕ* ·*q*_*a*_+(1-*ϕ*) ·*q*_*f*_, where *ϕ* indicates the prior probability that consumers believe that the product may be genuine [1,62]; *Q*_*a*_ and *Q*_*f*_ represent the product quality perceived by consumers when purchasing products through the genuine sales channel and counterfeit sales channel, respectively.

To supervise and inhibit counterfeiters, the brand owner can disclose the authenticity information related to the product’s quality to consumers with the help of the blockchain characteristics, such as traceability, openness and transparency, to help consumers identify genuine or counterfeit products when purchasing brand products. For this reason, under blockchain technology, consumers’ perceived product quality when purchasing products from the brand owner is *Q*_*a*_ = *q*_*a*_, while the perceived product quality when purchasing products from counterfeiters is *Q*_*f*_ = *q*_*f*_. The product quality perceived by consumers is consistent with the actual quality of the products in the market.

(2) Previous studies have revealed that product quality and consumer service experience positively affect product brand goodwill [8,53]. Therefore, considering the dynamic change in brand goodwill over time under the influence of product quality and service level, the state change equations of brand goodwill with and without blockchain are constructed.

When there is no blockchain, due to the asymmetry of product quality information, it is assumed that brand goodwill of both genuine and counterfeit products is *G*(*t*). Referring to the Nerlove-Arrow model [7,52], the dynamic state change equation of brand goodwill *G*(*t*) can be designed as follows:

$$\begin{array}{l} dG(t)=\lambda \cdot (\alpha_{f}q_{f}(t)-\sigma G(t))dt+(1-\lambda )\cdot (\alpha_{a}q_{a}(t)+\delta_{a}s_{a}(t)-\sigma G(t))dt\\ \;=\lambda \cdot (\alpha_{f}q_{f}(t))dt+(1-\lambda )\cdot (\alpha_{a}q_{a}(t)+\delta_{a}s_{a}(t))dt-\sigma G(t)dt\\ \;G(0)=G_{0} \end{array}$$ (1)

where *α*_*f*_ ·*q*_*f*_ (*t*), *α*_*a*_ ·*q*_*a*_ (*t*) and *δ*_*a*_ ·*s*_*a*_ (*t*) reflect the impact of the counterfeit product quality, genuine product quality and service level on brand goodwill, respectively, and *α*_*f*_, *α*_*a*_, *δ*_*a*_>0 are the corresponding influencing factors. In the absence of blockchain, the accumulation of brand goodwill is jointly influenced by genuine and counterfeit products. 0<*λ*<1 characterizes the degree or proportion of the impact of counterfeit products on brand goodwill, while (1-*λ*) describes the proportion of the impact of genuine products on brand goodwill; *s* >0 indicates the natural decay rate of brand goodwill, which is usually caused by customers forgetting brand products and market competition; and *G*_0_ is the initial value of brand goodwill. It should be noted that the quality of counterfeit products in Eq (1) is positive to the accumulation of brand goodwill, but the appearance of counterfeit products is actually unfavorable to brand goodwill. The reasons for this phenomenon can be explained as follows: if there are no counterfeit products, the item *λ*·(*α*_*f*_ ·*q*_*f*_ (*t*)-*sG*(*t*)) in Eq (1) should be *λ*·(*α*_*a*_·*q*_*a*_(*t*)-*sG*(*t*)), while the appearance of counterfeit products occupies a certain market share. Additionally, the quality of counterfeit products is lower than that of genuine products, so the brand goodwill of genuine products will be damaged.

Under blockchain technology, product quality information is symmetrical, and genuine and counterfeit products are different brand products for consumers. Therefore, it is assumed that brand goodwill under the sales channels of genuine products and counterfeit products is *G*_*a*_(*t*) and *G*_*f*_ (*t*), respectively. At this time, the dynamic state equations of genuine brand goodwill *G*_*a*_(*t*) and counterfeit product brand goodwill *G*_*f*_ (*t*) are as follows:

$$dG_{a}(t)=(\alpha_{a}q_{a}(t)+\delta_{a}s_{a}(t))dt-\sigma G_{a}(t)dt,\;G_{a}(0)=G_{0}$$ (2)

$$dG_{f}(t)=\alpha_{f}q_{f}(t)dt-\sigma G_{f}(t)dt,\;G_{f}(0)=G_{0}$$ (3)

Eqs (2) and (3) are obtained from *λ* = 0 and *λ* = 1 in Eq (1), respectively, and they show that blockchain technology separates the brand goodwill of genuine products from that of counterfeit products by identifying genuine and counterfeit products for consumers. In particular, the brand goodwill for genuine products is no longer affected by counterfeit products, and the brand goodwill for counterfeit products has nothing to do with genuine prod.

(3) In fact, consumer demand is affected not only by product price but also by product quality, service experience, brand image and other factors. Therefore, based on the demand model in the relevant literature [8,9], it is assumed that the consumer demand for the genuine product sales channel and counterfeit product sales channel are as follows:

$$d_{a}(t)=(1-a)\cdot e-p_{a}+\beta Q_{a}(t)+u\cdot (Q_{a}(t)-Q_{f}(t))+\gamma s_{a}(t)+\theta G_{a}(t)$$ (4)

$$d_{f}(t)=a\cdot e-p_{f}+\beta Q_{f}(t)+u\cdot (Q_{f}(t)-Q_{a}(t))+\theta G_{f}(t)$$ (5)

where *e* represents the potential market demand for this type of product, and *a* and (1-*a*) represent the proportion of potential market demand for this type of product for counterfeit products and genuine products, respectively [63]. Based on the research assumption by Tsay and Agrawal [64], parameter *e* is normalized to 1 because the potential market demand share of counterfeit products and genuine products can be reflected through parameter *a*, and parameter *e* does not affect the robustness of the relevant conclusions. Parameters *β*, *γ* and *θ* represent the influence coefficients of product quality, service level and brand goodwill perceived by consumers on consumer demand. In addition, *u*·(*Q*_*a*_^*DN*^(*t*)-*Q*_*f*_^*DN*^(*t*)) and *u*·(*Q*_*f*_^*DN*^(*t*)-*Q*_*a*_^*DN*^(*t*)) reflect the impact of consumers’ quality reference effect on the demand for genuine and counterfeit products, respectively. *u* is the quality reference effect coefficient [65]. As mentioned earlier, in the absence of blockchain, the goodwill for genuine brand *G*_*a*_(*t*) and the goodwill for counterfeit product brand *G*_*f*_ (*t*) are both *G*(*t*).

(4) Referring to the relevant research by Shen et al. [1] and Ma et al. [8], it is assumed that the product quality and service cost functions in genuine sales channels are *η*_*a*_·*q*_*a*_^2^(*t*)/2 and *k*_*a*_·*s*_*a*_^2^(*t*)/2, respectively, while the product quality cost function of counterfeiters is *η*_*f*_ ·*q*_*f*_^2^(*t*)/2. *η*_*a*_ and *η*_*f*_ are the product quality cost coefficients of the brand owner and counterfeiter, respectively, while *k*_*a*_ is the service cost coefficient of relevant decision-makers in the supply chain in the genuine sales channel. The setting of the cost function meets the general convexity assumption in economics [66]; the economic implication is that the cost function for product quality and service meets the law of marginal cost increase, while the marginal return of supply chain decision-makers related to product quality and service level is decreasing. In reality, the product quality cost usually refers to the R&D investment, labor investment, equipment purchases and maintenance costs of enterprises, while service cost can be understood as the daily operation and maintenance of stores and the time and energy expended by service personnel. In addition, compared with the counterfeiter, the brand owner usually has more professional production technology and quality standards, which have certain advantages in terms of product quality and cost. At the same time, because the counterfeiter’s production equipment may be backward and the production environment is simple, the unit cost for improving the quality of unit products is also high. Therefore, the product quality cost coefficient of the brand owner is less than that of the counterfeiter, that is, *η*_*a*_ <*η*_*f*_.

(5) Suppose that the fixed cost of introducing blockchain technology into a genuine sales channel is *F*, and the unit operating cost of implementing blockchain is *c*. The fixed cost *F* of the blockchain is generally the cost of purchasing some blockchain authentication machines, while the unit operating cost *c* generally represents the cost incurred when a supply chain member creates blockchain authentication information for consumers.

(6) To simplify the analysis without losing generality, based on the relevant research by Niu et al. [6], the production cost per unit product is assumed to be 0 because the models constructed in this paper and in the related literature are theoretical analytical models; this assumption does not affect the validity of the relevant conclusions. For example, Shen et al. [5], Liu et al. [36], and Ma et al. [53] made the same assumption. In addition, it is assumed that the relevant enterprises operate at time *t*∈[0, +∞) and that the counterfeiter, the brand owner and retailer have the same discount rate in the market in the same period.

Based on the above notation and assumptions, the rest of this study will use differential game theory to construct dynamic optimization models for the supply chain when the brand owner is in the direct sales mode or wholesale sales mode with no blockchain and blockchain adoption, respectively. Based on this foundation, we solve for the optimal product quality and service strategy for the genuine product sales channel and the counterfeit product quality under different scenarios and derive the optimal dynamic trajectories of the sales quantity, brand goodwill and expected discounted profit of the genuine and counterfeit products for the supply chain members.

### 3.3 Methodology

Considering the lack of dynamic optimization research on product quality and service decisions in terms of adopting blockchain technology to combat counterfeiting in the supply chain, this study uses the following research methodology: (1) differential games, (2) backward induction and (3) numerical analysis.

(1) According to the research by Ma et al. [8] and Ma and Hu [9], differential game theory is a particularly good approach when solving problems of dynamic optimization in continuous time. Compared with existing technologies, this paper focuses on a new exploration framework for studying the problem of dynamically combating counterfeit products with blockchain technology.

(2) Moreover, according to Xu et al. [38] and Zhong et al. [40], backward induction is an effective method for solving multistage supply chain optimization decision models. This paper therefore uses this method to solve the dynamic game problem between counterfeiters and genuine enterprises.

(3) Furthermore, according to Song et al. [55] and Meng et al. [59], numerical analysis is a popular method for verifying the validity of conclusions and analyzing complex solutions. We use this method to verify the generalization and robustness of the results in this paper.

## 4 Counterfeit product supervision in the brand owner’s direct sales mode

In this section, we consider that the brand owner is in a direct sales mode, as is the case for directly owned boutiques selling fashion luxury goods such as Louis Vuitton and Gucci, and study the case of counterfeit product sales in this environment without blockchain (denoted by *DN*) and the case of counterfeit product sales under blockchain technology (denoted by *DB*). Furthermore, there is a two-stage Stackelberg game between the brand owner and counterfeiter; that is, during the first stage, the brand owner determines the quality of the genuine products and the service level of its own stores, while during the second stage, the counterfeiter, as a market follower, determines the quality of its counterfeit products to obtain greater benefits.

### 4.1 No blockchain in the direct sales mode (*DN*)

In the case of *DN*, which involves counterfeit product sales in the direct sales mode by brand owners without blockchain, consumers usually cannot recognize the authenticity of the product, and the quality information regarding the genuine and counterfeit products is asymmetric. The product quality *Q*_*a*_^*DN*^(*t*) perceived by consumers in the genuine sales channel is the same as the product quality *Q*_*f*_^*DN*^(*t*) perceived in the counterfeit product sales channel, and both are *ϕ* ·*q*_*a*_^*DN*^(*t*)+(1-*ϕ*) ·*q*_*f*_^*DN*^(*t*). At this time, consumers’ demands for genuine products and counterfeit products are as follows:

$$d_{a}{}^{DN}(t)=(1-a)\cdot e-p_{a}+\beta Q_{a}{}^{DN}(t)+\gamma s_{a}{}^{DN}(t)+\theta G_{a}{}^{DN}(t)$$ (6)

$$d_{f}{}^{DN}(t)=a\cdot e-p_{f}+\beta Q_{f}{}^{DN}(t)+\theta G_{f}{}^{DN}(t)$$ (7)

From Eqs (6) and (7), we know that in the absence of blockchain, consumers’ quality reference effect on genuine and counterfeit products does not exist because *Q*_*a*_^*DN*^(*t*) = *Q*_*f*_^*DN*^(*t*), that is, *u*·(*Q*_*a*_^*DN*^(*t*)-*Q*_*f*_^*DN*^(*t*)) = *u*·(*Q*_*f*_^*DN*^(*t*)- *Q*_*a*_^*DN*^(*t*)) = 0.

The differential game model for counterfeiters and brands is constructed as follows:

$$\underset{q_{a}{}^{DN}(t),s_{a}{}^{DN}(t)}{\max}\{\begin{array}{l} \pi_{b}{}^{DN}=\int_{0}^{+\infty }e^{-\rho \cdot t}[p_{a}\cdot d_{a}{}^{DN}(t)-\frac{1}{2}\eta_{a}\cdot q_{a}{{}^{DN}}^{{}^{2}}(t)-\frac{1}{2}k_{a}\cdot s_{a}{{}^{DN}}^{{}^{2}}(t)]dt\\ \;=\int_{0}^{+\infty }e^{-\rho \cdot t}[p_{a}\cdot ((1-a)-p_{a}+\beta Q_{a}{}^{DN}(t)+\gamma s_{a}{}^{DN}(t)+\theta G_{a}{}^{DN}(t))-\frac{1}{2}\eta_{a}\cdot \\ \;q_{a}{{}^{DN}}^{{}^{2}}(t)-\frac{1}{2}k_{a}\cdot s_{a}{{}^{DN}}^{{}^{2}}(t)]dt \end{array}\}$$

$$\underset{q_{f}{}^{DN}(t)}{\max}\{\begin{array}{l} \pi_{f}{}^{DN}=\int_{0}^{+\infty }e^{-\rho \cdot t}[p_{f}\cdot d_{f}{}^{DN}(t)-\frac{1}{2}\eta_{f}\cdot q_{f}{{}^{DN}}^{{}^{2}}(t)]dt\\ \;=\int_{0}^{+\infty }e^{-\rho \cdot t}[p_{f}\cdot (a-p_{f}+\beta Q_{f}{}^{DN}(t)+\theta G_{f}{}^{DN}(t))-\frac{1}{2}\eta_{f}\cdot q_{f}{{}^{DN}}^{{}^{2}}(t)]dt \end{array}\}$$

$$s.t.\{\begin{array}{l} dG^{DN}(t)=\lambda \cdot (\alpha_{f}q_{f}{}^{DN}(t))dt+(1-\lambda )\cdot (\alpha_{a}q_{a}{}^{DN}(t)+\delta_{a}s_{a}{}^{DN}(t))dt-\sigma G^{DN}(t)dt\\ G^{DN}(0)=G_{0} \end{array}$$

#### Theorem 1

In the case of DN, there is no blockchain, and the brand owner is in the direct sales mode. The optimal product quality and service level of the brand owner are:

$$q_{a}{{}^{DN}}^{*}=\frac{p_{a}(\alpha_{a}(\theta -\theta \lambda )+\beta \phi (\rho +\sigma ))}{\eta_{a}(\rho +\sigma )}$$ (8)

$$s_{a}{{}^{DN}}^{*}=\frac{p_{a}(\gamma (\rho +\sigma )+\delta_{a}(\theta -\theta \lambda ))}{k_{a}(\rho +\sigma )}$$ (9)

The product quality decision by the counterfeiter is:

$$q_{f}{{}^{DN}}^{*}=\frac{p_{f}}{\eta_{f}}\cdot (\frac{\alpha_{f}\theta \lambda }{\rho +\sigma }-\beta \phi +\beta )$$ (10)

According to Theorem 1, we find that the quality *q*_*f*_^DN*^ of the counterfeit products is positively correlated with the sales price *p*_*f*_, while the optimal quality *q*_*a*_^DN*^ and the optimal service level *s*_*a*_^DN*^ of the genuine products are positively correlated with the sales price *p*_*a*_ of the genuine products. This relationship indicates that when brand owners increase the sales price of products, the quality of the genuine products and the service level of the stores improves. However, to conceal the product quality information, counterfeiters usually use a sales price that is similar to the brand product as the sales price of their own counterfeit products to deceive consumers into purchasing from them [4]. On the basis of imitating the sales price of genuine products, when the sales price of counterfeit products has the opportunity to increase, counterfeiters have more incentive to imitate the authenticity of products, improve the quality and sales of their products, and make more profits.

#### Corollary 1

In the case of DN, the optimal dynamic trajectory of brand goodwill is as follows:

$$\begin{array}{l} G^{DN}{}^{*}(t)=\frac{1}{\sigma }\cdot [s_{a}{{}^{DN}}^{*}\delta_{a}+e^{-\sigma \cdot t}(-s_{a}{{}^{DN}}^{*}\delta_{a}-(-1+e^{\sigma \cdot t})(q_{a}{{}^{DN}}^{*}\alpha_{a}(-1+\lambda )-q_{f}{{}^{DN}}^{*}\alpha_{f}\lambda \\ \;+s_{a}{{}^{DN}}^{*}\delta_{a}\lambda )+G_{0}\sigma )] \end{array}$$ (11)

The optimal dynamic trajectory of the sales quantity of genuine products is:


$$\begin{array}{l} d_{a}{{}^{DN}}^{*}(t)=1-a-p_{a}+s_{a}{{}^{DN}}^{*}\gamma +(\theta (-(q_{a}{{}^{DN}}^{*}\alpha_{a}+s_{a}{{}^{DN}}^{*}\delta_{a})(-1+\lambda )+q_{f}{{}^{DN}}^{*}\alpha_{f}\lambda ))/\sigma +\\ \;(e^{-\sigma \cdot t}\theta ((q_{a}{{}^{DN}}^{*}\alpha_{a}+s_{a}{{}^{DN}}^{*}\delta_{a})(-1+\lambda )-q_{f}{{}^{DN}}^{*}\alpha_{f}\lambda +G_{0}\sigma ))/\sigma -q_{f}{{}^{DN}}^{*}\beta (-\\ \;1+\phi )+q_{a}{{}^{DN}}^{*}\beta \phi \end{array}$$ (12)

The optimal dynamic trajectory of the sales quantity of counterfeit products is:

$$\begin{array}{l} d_{f}{{}^{DN}}^{*}(t)=a-p_{f}+(\theta (-(q_{a}{{}^{DN}}^{*}\alpha_{a}+s_{a}{{}^{DN}}^{*}\delta_{a})(-1+\lambda )+q_{a}{{}^{DN}}^{*}\alpha_{f}\lambda ))/\sigma +(e^{-\sigma \cdot t}\theta ((q_{a}{{}^{DN}}^{*}\\ \;\alpha_{a}+s_{a}{{}^{DN}}^{*}\delta_{a})(-1+\lambda )-q_{f}{{}^{DN}}^{*}\alpha_{f}\lambda +G_{0}\sigma ))/\sigma -q_{f}{{}^{DN}}^{*}\beta (-1+\phi )+q_{a}{{}^{DN}}^{*}\beta \phi \end{array}$$ (13)

The optimal dynamic trajectory of the expected discounted profit of the brand owner is:

$$\begin{array}{l} V_{b}{{}^{DN}}^{*}(t)=L_{b}{}^{DN}+(p_{a}\theta (s_{a}{{}^{DN}}^{*}\delta_{a}+e^{-\sigma \cdot t}(-s_{a}{{}^{DN}}^{*}\delta_{a}-(-1+e^{\sigma \cdot t})(q_{a}{{}^{DN}}^{*}\alpha_{a}(-1+\lambda )-q_{f}{{}^{DN}}^{*}\\ \;\alpha_{f}\lambda +s_{a}{{}^{DN}}^{*}\delta_{a}\lambda )+G_{0}\sigma )))/(\sigma (\rho +\sigma )) \end{array}$$ (14)

The optimal dynamic trajectory of the expected discounted profit obtained by the counterfeiter is:

$$\begin{array}{l} V_{f}{{}^{DN}}^{*}(t)=L_{f}{}^{DN}+(p_{f}\theta (s_{a}{{}^{DN}}^{*}\delta_{a}+e^{-\sigma \cdot t}(-s_{a}{{}^{DN}}^{*}\delta_{a}-(-1+e^{\sigma \cdot t})(q_{a}{{}^{DN}}^{*}\alpha_{a}(-1+\lambda )-q_{f}{{}^{DN}}^{*}\alpha_{f}\lambda \\ \;+s_{a}{{}^{DN}}^{*}\delta_{a}\lambda )+G_{0}\sigma )))/(\sigma (\rho +\sigma )) \end{array}$$ (15)

$$where\;\begin{array}{l} L_{f}{}^{DN}=(-((q_{f}{{}^{DN}}^{*}{}^{{}^{2}}\eta_{f})/2)+(p_{f}\theta ((-q_{a}{{}^{DN}}^{*}\alpha_{a}-s_{a}{{}^{DN}}^{*}\delta_{a})(-1+\lambda )+q_{f}{{}^{DN}}^{*}\alpha_{f}\lambda ))/(\rho +\sigma )+p_{f}(\\ \;a-p_{f}+\beta (q_{f}{{}^{DN}}^{*}+q_{a}{{}^{DN}}^{*}\phi -q_{f}{{}^{DN}}^{*}\phi )))/\rho \end{array};$$

$$\begin{array}{l} L_{b}{}^{DN}=(-((k_{a}s_{a}{{}^{DN}}^{*}{}^{{}^{2}})/2)-(q_{a}{{}^{DN}}^{*}{}^{{}^{2}}\eta_{a})/2+(p_{a}\theta ((-q_{a}{{}^{DN}}^{*}\alpha_{a}-s_{a}{{}^{DN}}^{*}\delta_{a})(-1+\lambda )+q_{f}{{}^{DN}}^{*}\alpha_{f}\lambda ))/(\rho +\sigma \\ \;)+p_{a}(1-a-p_{a}+s_{a}{{}^{DN}}^{*}\gamma +\beta (q_{f}{{}^{DN}}^{*}+q_{a}{{}^{DN}}^{*}\phi -q_{f}{{}^{DN}}^{*}\phi )))/\rho \end{array}.$$

Corollary 1 shows that in the absence of blockchain, brand goodwill changes dynamically over time under the joint influence of the optimal quality *q*_*a*_^DN*^ and optimal service level *s*_*a*_^DN*^ of genuine products as well as the quality *q*_*f*_^DN*^ of counterfeit products. This phenomenon implies that when consumers buy counterfeit products, the poor quality of counterfeit products may affect consumers’ reputation for the brand products, affect the brand goodwill of the products, affect the sales of genuine products, and ultimately damage the profits of brand owners, which may explain the pain experienced by brand owners in the face of counterfeit product sales in reality. In addition, under the dynamic influence of brand goodwill, the consumer demand for genuine and counterfeit products and the expected discounted profits for the brand owner and counterfeiter also change dynamically over time.

### 4.2 Blockchain adoption in the direct sales mode (*DB*)

Different from the case without blockchain, in the case of *DB*, where the brand owner adopts blockchain to supervise and prevent the sales of counterfeit products, the brand owner can record information about the products, including the origin, production and processing, logistics and sales in the blockchain’s distributed network. The recorded information cannot be tampered with, and consumers can be authorized to access it; therefore, the brand can verify the authenticity of product quality information [1]. Therefore, quality information about genuine and counterfeit products is symmetrical for consumers. When a consumer purchases a product through a genuine sales channel, the prior probability *ϕ* = 1 represents the likelihood that the consumer believes that the product may be genuine, and the perceived product quality is expressed as *Q*_*a*_^*DB*^(*t*) = *ϕ* ·*q*_*a*_^*DB*^(*t*)+(1-*ϕ*)·*q*_*f*_^*DB*^(*t*) = *q*_*a*_^*DB*^(*t*). However, when consumers buy products through the counterfeit sales channel, because the counterfeiter cannot provide traceable services that verify the authenticity of the products to consumers, the prior probability *ϕ* = 0 represents the likelihood that consumers believe that the products may be genuine, and the perceived product quality is expressed as *Q*_*f*_^*DB*^(*t*) = *ϕ*·*q*_*a*_^*DB*^(*t*)+(1-*ϕ*)·*q*_*f*_^*DB*^(*t*) = *q*_*f*_^*DB*^(*t*). At this time, the brand owner can use blockchain technology to supervise the product quality information. The demand for genuine products and the demand for counterfeit products can be expressed as follows:

$$d_{a}{}^{DB}(t)=(1-a)\cdot e-p_{a}+\beta Q_{a}{}^{DB}(t)+u\cdot (Q_{a}{}^{DB}(t)-Q_{f}{}^{DB}(t))+\gamma s_{a}{}^{DB}(t)+\theta G_{a}{}^{DB}(t)$$ (16)

$$d_{f}{}^{DB}(t)=a\cdot e-p_{f}+\beta Q_{f}{}^{DB}(t)+u\cdot (Q_{f}{}^{DB}(t)-Q_{a}{}^{DB}(t))+\theta G_{f}{}^{DB}(t)$$ (17)

Under blockchain technology, the consumers’ perceived quality of the genuine and counterfeit products is different, that is, *Q*_*a*_^*DB*^(*t*)≠ *Q*_*f*_^*DB*^(*t*). At this time, the consumers’ quality reference effect affects the sales of genuine and counterfeit products, which is different from the case without blockchain.

Combined with the symbol description and assumptions in Section 3, the differential game model that allows the brand owner to supervise the sale of counterfeit products in the blockchain environment under the direct sales mode is constructed as follows:

$$\underset{q_{a}{}^{DB}(t),s_{a}{}^{DB}(t)}{\max}\{\pi_{b}{}^{DB}=\int_{0}^{+\infty }e^{-\rho \cdot t}[(p_{a}-c)\cdot d_{a}{}^{DB}(t)-\frac{1}{2}\eta_{a}\cdot q_{a}{{}^{DB}}^{{}^{2}}(t)-\frac{1}{2}k_{a}\cdot s_{a}{{}^{DB}}^{{}^{2}}(t)]dt-F\}$$

$$s.t.\{\begin{array}{l} dG_{a}{}^{DB}(t)=(\alpha_{a}q_{a}{}^{DB}(t)+\delta_{a}s_{a}{}^{DB}(t))dt-\sigma G_{a}{}^{DB}(t)dt,\\ G_{a}{}^{DB}(0)=G_{0} \end{array}$$

$$\underset{q_{f}{}^{DB}(t)}{\max}\{\pi_{f}{}^{DB}=\int_{0}^{+\infty }e^{-\rho \cdot t}[p_{f}\cdot d_{f}{}^{DB}(t)-\frac{1}{2}\eta_{f}\cdot q_{f}{{}^{DB}}^{{}^{2}}(t)]dt\}$$

$$s.t.\{\begin{array}{l} dG_{f}{}^{DB}(t)=\alpha_{f}q_{f}{}^{DN}(t)dt-\sigma G_{f}{}^{DB}(t)dt,\\ G_{f}{}^{DB}(0)=G_{0} \end{array}$$

#### Theorem 2

In the case of DB, the brand owner in the direct sales mode adopts blockchain, and the optimal product quality and service level of the brand owner are expressed as follows:

$$q_{a}{{}^{DB}}^{*}=\frac{\left(p_{a}-c)(\alpha_{a}\theta +(\beta +\mu )(\rho +\sigma )\right)}{\eta_{a}(\rho +\sigma )}$$ (18)

$$s_{a}{{}^{DB}}^{*}=\frac{\left(p_{a}-c)(\gamma (\rho +\sigma )+\delta_{a}\theta \right)}{k_{a}(\rho +\sigma )}$$ (19)

The quality level of counterfeit products is:

$$q_{f}{{}^{DB}}^{*}=\frac{p_{f}}{\eta_{f}}\cdot (\frac{\alpha_{f}\theta }{\rho +\sigma }+\beta +\mu )$$ (20)

Unlike Theorem 1, in Theorem 2, the decisions by the brand owner and counterfeiters have nothing to do with the prior probability *ϕ* that consumers believe that products may be genuine. This shows that blockchain technology eliminates the asymmetry of product quality information and realizes the supervision of product quality from the perspective of enterprise decision-making. However, due to the role of blockchain, in this situation, consumers have a reference quality effect (*u*) for products, which affects product quality decisions by both brands and counterfeiters (note: parameter analysis is provided in Section 6).

#### Corollary 2

*In the case of DB*, *the optimal dynamic trajectories of genuine and counterfeit brand goodwill are expressed as follows*:

$$G_{a}{{}^{DB}}^{*}(t)=(q_{a}{{}^{DB}}^{*}\alpha_{a}+s_{a}{{}^{DB}}^{*}\delta_{a}-e^{-\sigma \cdot t}(q_{a}{{}^{DB}}^{*}\alpha_{a}+s_{a}{{}^{DB}}^{*}\delta_{a}-G_{0}\sigma ))/\sigma$$ (21)

$$G_{f}{{}^{DB}}^{*}(t)=e^{-\sigma \cdot t}(G_{0}-\frac{q_{f}{{}^{DB}}^{*}\alpha_{f}}{\sigma })+\frac{q_{f}{{}^{DB}}^{*}\alpha_{f}}{\sigma }$$ (22)

*The optimal dynamic trajectories of the sales quantities of genuine and counterfeit products are*:

$$\begin{array}{l} d_{a}{{}^{DB}}^{*}(t)=1-a-p_{a}+q_{a}{{}^{DB}}^{*}\beta +s_{a}{{}^{DB}}^{*}\gamma +(q_{a}{{}^{DB}}^{*}-q_{f}{{}^{DB}}^{*})\mu +e^{-\sigma \cdot t}\theta (G_{0}-(q_{a}{{}^{DB}}^{*}\alpha_{a}\\ \;+s_{a}{{}^{DB}}^{*}\delta_{a})/\sigma )+((q_{a}{{}^{DB}}^{*}\alpha_{a}+s_{a}{{}^{DB}}^{*}\delta_{a})\theta )/\sigma \end{array}$$ (23)

$$\begin{array}{l} d_{f}{{}^{DB}}^{*}(t)=a-p_{f}+q_{f}{{}^{DB}}^{*}\beta +u(-q_{a}{{}^{DB}}^{*}+q_{f}{{}^{DB}}^{*})+e^{-\sigma \cdot t}\theta (G_{0}-(q_{f}{{}^{DB}}^{*}\alpha_{f})/\sigma )\\ \;+(q_{f}{{}^{DB}}^{*}\alpha_{f}\theta )/\sigma \end{array}$$ (24)

*The optimal dynamic trajectories of the expected discounted profits for the brand owner and counterfeiter are*:

$$\begin{array}{l} V_{b}{{}^{DB}}^{*}(t)=L_{b}{}^{DB}+((-c+p_{a})\theta (q_{a}{{}^{DB}}^{*}\alpha_{a}+s_{a}{{}^{DB}}^{*}\delta_{a}-e^{-\sigma \cdot t}(q_{a}{{}^{DB}}^{*}\alpha_{a}+s_{a}{{}^{DB}}^{*}\delta_{a}-G_{0}\sigma \\ \;)))/(\sigma (\rho +\sigma )) \end{array}$$ (25)

$$V_{f}{{}^{DB}}^{*}(t)=L_{f}{}^{DB}+\frac{p_{f}\theta ((-1+e^{\sigma \cdot t})q_{f}{{}^{DB}}^{*}\alpha f+G_{0}\sigma )}{e^{\sigma \cdot t}(\sigma (\rho +\sigma ))}$$ (26)

$$\mathrm{where}\;L_{f}{}^{DB}=(-((q_{f}{{}^{DB}}^{*}{}^{{}^{2}}\eta_{f})/2)+p_{f}(a-p_{f}-q_{a}{{}^{DB}}^{*}\mu +q_{f}{{}^{DB}}^{*}(\beta +\mu ))+(p_{f}q_{f}{{}^{DB}}^{*}\alpha_{f}\theta )/(\rho +\sigma ))/\rho ;$$

$$\begin{array}{l} L_{b}{}^{DB}=(-((k_{a}s_{a}{{}^{DB}}^{*}{}^{{}^{2}})/2)-(q_{a}{{}^{DB}}^{*}{}^{{}^{2}}\eta_{a})/2+(c-p_{a})(-1+a+p_{a}-s_{a}{{}^{DB}}^{*}\gamma +q_{f}{{}^{DB}}^{*}\mu -q_{a}{{}^{DB}}^{*}(\beta \\ \;+\mu ))-F\rho +((-c+p_{a})(q_{a}{{}^{DB}}^{*}\alpha_{a}+s_{a}{{}^{DB}}^{*}\delta_{a})\theta )/(\rho +\sigma ))/\rho \end{array}.$$

Corollary 2 reveals that the brand owner helps consumers identify counterfeit products and genuine products with the support of blockchain technology. We find that although the optimal dynamic trajectories of both genuine brand goodwill and counterfeit product brand goodwill are not affected by the other party’s product quality decision, the dynamic trajectories of the consumers’ demand for genuine products and the sales quantity of counterfeit products are related to the optimal quality decision by both parties due to the role of blockchain technology, which provides consumers with a reference regarding the quality of genuine and counterfeit products. At the same time, the optimal dynamic trajectories of the expected discounted profits for the counterfeiter and brand owner are also affected by each other’s quality decisions. Moreover, the quality reference effect of consumers may be beneficial to the brand owner because the quality of genuine products is usually better than that of counterfeit products, which helps to increase the consumer demand for genuine products and reduce the sales market for counterfeit products.

## 5 Counterfeit product supervision in the brand owner’s wholesale sales mode

In reality, the wholesale sales mode, in which brand owners sell through offline retailers, is relatively common. For example, brand enterprises such as Mengniu and Yili wholesale their products to retail enterprises such as Walmart for offline sales. This section discusses the situation in which counterfeit products are sold when there is no blockchain under the brand owner’s wholesale sales mode (denoted by *WN*), the situation in which the brand owner establishes blockchain to supervise the sale of counterfeit products (denoted by *WB*) and the situation in which the retailer establishes blockchain to supervise the sale of counterfeit products (denoted by *WB*). Although the implementation of blockchain requires joint participation and information collaboration between upstream and downstream enterprises in the supply chain, the adoption and establishment of blockchain may be initiated by the brand owner or retailer. For example, Walmart cooperates with IBM to develop blockchain technology for brands such as Nestle and Dole Food [1]. Thus, we consider two cases of blockchain adoption (i.e., *WB* and *WR*) and study the impact of blockchain adoption by the brand owner or retailer on the optimal decision-making in the supply chain and the effect of supervising the sale of counterfeit products in the wholesale sales mode, which is rare in the existing relevant studies. In this section, there are three stages of the Stackelberg game among the brand owner, retailer and counterfeiter. In the first stage, the brand owner determines the quality of the genuine products and wholesale products to the retailer; in the second stage, the retailer determines the service level of their stores and sells genuine products to consumers; and in the third stage, the counterfeiter determines the quality of its counterfeit products and sells them.

### 5.1 No blockchain in the wholesale sales mode (*WN*)

As a benchmark, we first consider the *WN* case in which there are counterfeit product sales under the brand owner’s wholesale sales mode without blockchain. Similar to the *DN* case without blockchain in the direct sales mode, the consumer demand for genuine and counterfeit products in the *WN* case is expressed as follows:

$$d_{a}^{WN}(t)=(1-a)\cdot e-p_{a}+\beta Q_{a}^{WN}(t)+\gamma S_{a}^{WN}(t)+\theta G_{a}^{WN}(t)$$ (27)

$$d_{f}{}^{WN}(t)=a\cdot e-p_{f}+\beta Q_{f}{}^{WN}(t)+\theta G_{f}{}^{WN}(t)$$ (28)

where *Q*_*a*_^*WN*^(*t*) = *Q*_*f*_^*WN*^(*t*) = *ϕ* ·*q*_*a*_^*WN*^(*t*)+(1-*ϕ*) ·*q*_*f*_^*WN*^(*t*). Furthermore, the differential game model of the brand owner, retailer and counterfeiter in the *WN* case is constructed as follows:

$$\underset{q_{a}{}^{DN}(t)}{\max}\{\pi_{b}{}^{WN}=\int_{0}^{+\infty }e^{-\rho \cdot t}[w_{a}\cdot d_{a}{}^{WN}(t)-\frac{1}{2}\eta_{a}\cdot q_{a}{{}^{WN}}^{{}^{2}}(t)]dt\}$$

$$\underset{s_{a}{}^{WN}(t)}{\max}\{\pi_{r}{}^{DN}=\int_{0}^{+\infty }e^{-\rho \cdot t}[(p_{a}-w_{a})\cdot d_{a}{}^{WN}(t)-\frac{1}{2}k_{a}\cdot s_{a}{{}^{WN}}^{{}^{2}}(t)]dt\}$$

$$\underset{q_{f}{}^{WN}(t)}{\max}\{\pi_{f}{}^{WN}=\int_{0}^{+\infty }e^{-\rho \cdot t}[p_{f}\cdot d_{f}{}^{WN}(t)-\frac{1}{2}\eta_{f}\cdot q_{f}{{}^{WN}}^{{}^{2}}(t)]dt\}$$

$$s.t.\{\begin{array}{l} dG^{WN}(t)=\lambda \cdot (\alpha_{f}q_{f}{}^{WN}(t))dt+(1-\lambda )\cdot (\alpha_{a}q_{a}{}^{WN}(t)+\delta_{a}s_{a}{}^{WN}(t))dt-\sigma G^{WN}(t)dt\\ G^{WN}(0)=G_{0} \end{array}$$

#### Theorem 3

In the case of WN, there is no blockchain, and the brand owner is in the wholesale sales mode. The optimal product quality of the brand owner and the optimal service level of the retailer are:

$$q_{a}{{}^{WN}}^{*}=\frac{w_{a}}{\eta_{a}}\cdot (\frac{\alpha_{a}\theta (1-\lambda )}{\rho +\sigma }+\beta \phi )$$ (29)

$$s_{a}{{}^{WN}}^{*}=\frac{\left(p_{a}-w_{a}\right)}{k_{a}}\cdot (\frac{\delta_{a}\theta (1-\lambda )}{\rho +\sigma }+\gamma )$$ (30)

The product quality determined by the counterfeiter is:

$$q_{f}{{}^{WN}}^{*}=\frac{p_{f}}{\eta_{f}}\cdot (\frac{\alpha_{f}\theta \lambda }{\rho +\sigma }-\beta \phi +\beta )$$ (31)

#### Corollary 3

In the case of WN, the optimal dynamic trajectory of brand goodwill is:

$$\begin{array}{l} G^{WN}{}^{*}(t)=e^{-\sigma \cdot t}(G_{0}-((q_{a}{{}^{WN}}^{*}\alpha_{a}+s_{a}{{}^{WN}}^{*}\delta_{a})(1-\lambda )+q_{f}{{}^{WN}}^{*}\alpha_{f}\lambda )/\sigma )+((q_{a}{{}^{WN}}^{*}\alpha_{a}+s_{a}{{}^{WN}}^{*}\delta_{a})\\ \;(1-\lambda )+q_{f}{{}^{WN}}^{*}\alpha_{f}\lambda )/\sigma \end{array}$$ (32)

The optimal dynamic trajectories of the sales quantities of genuine and counterfeit products are:

$$\begin{array}{l} d_{a}{{}^{WN}}^{*}(t)=1-a-p_{a}+s_{a}{{}^{WN}}^{*}\gamma +\theta (((q_{a}{{}^{WN}}^{*}\alpha_{a}+s_{a}{{}^{WN}}^{*}\delta_{a})(1-\lambda )+q_{f}{{}^{WN}}^{*}\alpha_{f}\lambda )/\sigma +e^{-\sigma \cdot t}(G_{0}\\ \;-((q_{a}{{}^{WN}}^{*}\alpha_{a}+s_{a}{{}^{WN}}^{*}\delta_{a})(1-\lambda )+q_{f}{{}^{WN}}^{*}\alpha_{f}\lambda )/\sigma ))+\beta (q_{f}{{}^{WN}}^{*}(1-\phi )+q_{a}{{}^{WN}}^{*}\phi ) \end{array}$$ (33)

$$\begin{array}{l} d_{f}{{}^{WN}}^{*}(t)=a-p_{f}+\theta (((q_{a}{{}^{WN}}^{*}\alpha_{a}+s_{a}{{}^{WN}}^{*}\delta_{a})(1-\lambda )+q_{f}{{}^{WN}}^{*}\alpha_{f}\lambda )/\sigma +e^{-\sigma \cdot t}(G_{0}-((q_{a}{{}^{WN}}^{*}\alpha_{a}\\ \;+s_{a}{{}^{WN}}^{*}\delta_{a})(1-\lambda )+q_{f}{{}^{WN}}^{*}\alpha_{f}\lambda )/\sigma ))+\beta (q_{f}{{}^{WN}}^{*}(1-\phi )+q_{a}{{}^{WN}}^{*}\phi ) \end{array}$$ (34)

The optimal dynamic trajectories of the expected discounted profits for the brand owner, retailer and counterfeiter are:

$$\begin{array}{l} V_{b}{{}^{WN}}^{*}(t)=L_{b}{}^{WN}+(w_{a}\theta (((q_{a}{{}^{WN}}^{*}\alpha_{a}+s_{a}{{}^{WN}}^{*}\delta_{a})(1-\lambda )+q_{f}{{}^{WN}}^{*}\alpha_{f}\lambda )/\sigma +e^{-\sigma \cdot t}(G_{0}-((q_{a}{{}^{WN}}^{*}\alpha_{a}\\ \;+s_{a}{{}^{WN}}^{*}\delta_{a})(1-\lambda )+q_{f}{{}^{WN}}^{*}\alpha_{f}\lambda )/\sigma ))/(\rho +\sigma ) \end{array}$$ (35)

$$\begin{array}{l} V_{r}{{}^{WN}}^{*}(t)=L_{r}{}^{WN}+(p_{a}-w_{a})\theta (((q_{a}{{}^{WN}}^{*}\alpha_{a}+s_{a}{{}^{WN}}^{*}\delta_{a})(1-\lambda )+q_{f}{{}^{WN}}^{*}\alpha_{f}\lambda )/\sigma +e^{-\sigma \cdot t}(G_{0}-((\\ \;q_{a}{{}^{WN}}^{*}\alpha_{a}+s_{a}{{}^{WN}}^{*}\delta_{a})(1-\lambda )+q_{f}{{}^{WN}}^{*}\alpha_{f}\lambda )/\sigma ))/(\rho +\sigma ) \end{array}$$ (36)

$$\begin{array}{l} V_{f}{{}^{WN}}^{*}(t)=L_{f}{}^{WN}+(p_{f}\theta (((q_{a}{{}^{WN}}^{*}\alpha_{a}+s_{a}{{}^{WN}}^{*}\delta_{a})(1-\lambda )+q_{f}{{}^{WN}}^{*}\alpha_{f}\lambda )/\sigma +e^{-\sigma \cdot t}(G_{0}-((q_{a}{{}^{WN}}^{*}\alpha_{a}\\ \;+s_{a}{{}^{WN}}^{*}\delta_{a})(1-\lambda )+q_{f}{{}^{WN}}^{*}\alpha_{f}\lambda )/\sigma ))/(\rho +\sigma ) \end{array}$$ (37)

$$\mathrm{where}\;\begin{array}{l} L_{f}{}^{WN}=(-((q_{f}{{}^{WN}}^{*}{}^{{}^{2}}\eta_{f})/2)+(p_{f}\theta ((-q_{a}{{}^{WN}}^{*}\alpha_{a}-s_{a}{{}^{WN}}^{*}\delta_{a})(-1+\lambda )+q_{f}{{}^{WN}}^{*}\alpha_{f}\lambda ))/(\rho +\sigma )+p_{f}(\\ \;a-p_{f}+\beta (q_{f}{{}^{WN}}^{*}+q_{a}{{}^{WN}}^{*}\phi -q_{f}{{}^{WN}}^{*}\phi )))/\rho \end{array};$$

$$\begin{array}{l} L_{b}{}^{WN}=(-(q_{a}{{}^{WN}}^{*}{}^{{}^{2}}\eta_{a})/2+(w_{a}\theta ((-q_{a}{{}^{WN}}^{*}\alpha_{a}-s_{a}{{}^{WN}}^{*}\delta_{a})(-1+\lambda )+q_{f}{{}^{WN}}^{*}\alpha_{f}\lambda ))/(\rho +\sigma )+w_{a}(1-a-p_{a}\\ \;+s_{a}{{}^{WN}}^{*}\gamma +\beta (q_{f}{{}^{WN}}^{*}+q_{a}{{}^{WN}}^{*}\phi -q_{f}{{}^{WN}}^{*}\phi )))/\rho \end{array};$$

$$\begin{array}{l} L_{r}{}^{WN}=(-((k_{a}s_{a}{{}^{WN}}^{*}{}^{{}^{2}})/2)+((p_{a}-w_{a})\theta ((-q_{a}{{}^{WN}}^{*}\alpha_{a}-s_{a}{{}^{WN}}^{*}\delta_{a})(-1+\lambda )+q_{f}{{}^{WN}}^{*}\alpha_{f}\lambda ))/(\rho +\sigma )+(p_{a}\\ \;-w_{a})(1-a-p_{a}+s_{a}{{}^{WN}}^{*}\gamma +\beta (q_{f}{{}^{WN}}^{*}+q_{a}{{}^{WN}}^{*}\phi -q_{f}{{}^{WN}}^{*}\phi )))/\rho \end{array}.$$

### 5.2 Blockchain establishment by the brand owner in the wholesale sales mode (*WB*)

In the wholesale sales mode, this section considers the *WB* case in which the brand owner adopts blockchain to supervise the sale of counterfeit products. The fixed cost *F* and unit operating cost *c* of establishing blockchain in the genuine sales channel is determined by the brand owner, while the brand owner, retailer and consumers jointly participate in the blockchain implementation process. Similar to the *DB* case in which the brand owner adopts blockchain in the direct sales mode, the consumer demand for genuine and counterfeit products in the *WB* case is expressed as follows:

$$d_{a}{}^{WB}(t)=(1-a)\cdot e-p_{a}+\beta Q_{a}{}^{WB}(t)+u\cdot (Q_{a}{}^{WB}(t)-Q_{f}{}^{WB}(t))+\gamma s_{a}{}^{WB}(t)+\theta G_{a}{}^{WB}(t)$$ (38)

$$d_{f}{}^{WB}(t)=a\cdot e-p_{f}+\beta Q_{f}{}^{WB}(t)+u\cdot (Q_{f}{}^{WB}(t)-Q_{a}{}^{WB}(t))+\theta G_{f}{}^{WB}(t)$$ (39)

where *Q*_*a*_^*WB*^(*t*) = *q*_*a*_^*WB*^(*t*) and *Q*_*f*_^*WB*^(*t*) = *q*_*f*_^*WB*^(*t*). In this case, the differential game model of the brand owner, retailer and counterfeiter is constructed as follows:

$$\underset{q_{a}{}^{WB}(t)}{\max}\{\pi_{b}{}^{WB}=\int_{0}^{+\infty }e^{-\rho \cdot t}[(w_{a}-c)\cdot d_{a}{}^{WB}(t)-\frac{1}{2}\eta_{a}\cdot q_{a}{{}^{WB}}^{{}^{2}}(t)-\frac{1}{2}k_{a}\cdot s_{a}{{}^{WB}}^{{}^{2}}(t)]dt-F\}$$

$$\underset{s_{a}{}^{WB}(t)}{\max}\{\pi_{r}{}^{WB}=\int_{0}^{+\infty }e^{-\rho \cdot t}[(p_{a}-w_{a})\cdot d_{a}{}^{WB}(t)-\frac{1}{2}k_{a}\cdot s_{a}{{}^{WB}}^{{}^{2}}(t)]dt\}$$

$$s.t.\{\begin{array}{l} dG_{a}{}^{WB}(t)=(\alpha_{a}q_{a}{}^{WB}(t)+\delta_{a}s_{a}{}^{WB}(t))dt-\sigma G_{a}{}^{WB}(t)dt\\ G_{a}{}^{WB}(0)=G_{0} \end{array}$$

$$\underset{q_{f}{}^{WB}(t)}{\max}\{\pi_{f}{}^{WB}=\int_{0}^{+\infty }e^{-\rho \cdot t}[p_{f}\cdot d_{f}{}^{WB}(t)-\frac{1}{2}\eta_{f}\cdot q_{f}{{}^{WB}}^{{}^{2}}(t)]dt\}$$

$$s.t.dG_{f}{}^{WB}(t)=\alpha_{f}q_{f}{}^{WB}(t)dt-\sigma G_{f}{}^{WB}(t)dt,\;G_{f}{}^{WB}(0)=G_{0}$$

#### Theorem 4

In the case of WB, the brand owner in the wholesale sales mode establishes blockchain, and the optimal product quality of the brand owner and the optimal service level of the retailer are:

$$q_{a}{{}^{WB}}^{*}=\frac{\left(w_{a}-c\right)}{\eta_{a}}\cdot (\frac{\alpha_{a}\theta }{\rho +\sigma }+\beta +\mu )$$ (40)

$$s_{a}{{}^{WB}}^{*}=\frac{\left(p_{a}-w_{a}\right)}{k_{a}}(\frac{\delta_{a}\theta }{\rho +\sigma }+\gamma )$$ (41)

*The product quality determined by the counterfeiter is*:

$$q_{f}{{}^{WB}}^{*}=\frac{p_{f}}{\eta_{f}}\cdot (\frac{\alpha_{f}\theta }{\rho +\sigma }+\beta +\mu )$$ (42)

#### Corollary 4

*In the case of WB*, *the optimal dynamic trajectories of genuine and counterfeit brand goodwill are*:

$$G_{a}{{}^{WB}}^{*}(t)=(q_{a}{{}^{WB}}^{*}\alpha_{a}+s_{a}{{}^{WB}}^{*}\delta_{a}-e^{-\sigma \cdot t}(q_{a}{{}^{WB}}^{*}\alpha_{a}+s_{a}{{}^{WB}}^{*}\delta_{a}-G_{0}\sigma ))/\sigma$$ (43)

$$G_{f}{{}^{WB}}^{*}(t)=e^{-\sigma \cdot t}(G_{0}-\frac{q_{f}{{}^{WB}}^{*}\alpha_{f}}{\sigma })+\frac{q_{f}{{}^{WB}}^{*}\alpha_{f}}{\sigma }$$ (44)

The optimal dynamic trajectories of the sales quantities of genuine and counterfeit products are:

$$\begin{array}{l} d_{a}{{}^{WB}}^{*}(t)=1-a-p_{a}+q_{a}{{}^{WB}}^{*}\beta +s_{a}{{}^{WB}}^{*}\gamma +(q_{a}{{}^{WB}}^{*}-q_{f}{{}^{WB}}^{*})\mu +e^{-\sigma \cdot t}\theta (G_{0}-(q_{a}{{}^{WB}}^{*}\alpha_{a}+s_{a}{{}^{WB}}^{*}\\ \;\delta_{a})/\sigma )+((q_{a}{{}^{WB}}^{*}\alpha_{a}+s_{a}{{}^{WB}}^{*}\delta_{a})\theta )/\sigma \end{array}$$ (45)

$$\begin{array}{l} d_{f}{{}^{WB}}^{*}(t)=a-p_{f}+q_{f}{{}^{WB}}^{*}\beta +(-q_{a}{{}^{WB}}^{*}+q_{f}{{}^{WB}}^{*})u+e^{-\sigma \cdot t}\theta (G_{0}-(q_{f}{{}^{WB}}^{*}\alpha_{f})/\sigma )+\\ \;(q_{f}{{}^{WB}}^{*}\alpha_{f}\theta )/\sigma \end{array}$$ (46)

The optimal dynamic trajectories of the expected discounted profits for the brand owner, retailer and counterfeiter are:

$$\begin{array}{l} V_{b}{{}^{WB}}^{*}(t)=L_{b}{}^{WB}+(-c+w_{a})\theta ((q_{a}{{}^{WB}}^{*}\alpha_{a}+s_{a}{{}^{WB}}^{*}\delta_{a}-e^{-\sigma \cdot t}(q_{a}{{}^{WB}}^{*}\alpha_{a}+s_{a}{{}^{WB}}^{*}\delta_{a}-G_{0}\sigma ))/\sigma \\ \;)/(\rho +\sigma ) \end{array}$$ (47)

$$\begin{array}{l} V_{r}{{}^{DB}}^{*}(t)=L_{r}{}^{DB}+(-w_{a}+p_{a})\theta ((q_{a}{{}^{WB}}^{*}\alpha_{a}+s_{a}{{}^{WB}}^{*}\delta_{a}-e^{-\sigma \cdot t}(q_{a}{{}^{WB}}^{*}\alpha_{a}+s_{a}{{}^{WB}}^{*}\delta_{a}-G_{0}\sigma \\ \;))/\sigma )/(\sigma (\rho +\sigma )) \end{array}$$ (48)

$$V_{f}{{}^{WB}}^{*}(t)=L_{f}{}^{WB}+\frac{p_{f}\theta }{\left(\rho +\sigma \right)}\cdot (e^{-\sigma \cdot t}(G_{0}-\frac{q_{f}{{}^{WB}}^{*}\alpha_{f}}{\sigma })+\frac{q_{f}{{}^{WB}}^{*}\alpha_{f}}{\sigma })$$ (49)

$$where\;\begin{array}{l} L_{f}{}^{WB}=(-((q_{f}{{}^{WB}}^{*}{}^{{}^{2}}\eta_{f})/2)+p_{f}(a-p_{f}-q_{a}{{}^{WB}}^{*}\mu +q_{f}{{}^{WB}}^{*}(\beta +\mu ))+(p_{f}q_{f}{{}^{WB}}^{*}\alpha_{f}\theta )\\ \;/(\rho +\sigma ))/\rho \end{array};$$

$$\begin{array}{l} L_{b}{}^{WB}=(-(q_{a}{{}^{WB}}^{*}{}^{{}^{2}}\eta_{a})/2+(c-w_{a})(-1+a+p_{a}-s_{a}{{}^{WB}}^{*}\gamma +q_{f}{{}^{WB}}^{*}\mu -q_{a}{{}^{WB}}^{*}(\beta +\mu ))-F\rho +\\ \;((-c+w_{a})(q_{a}{{}^{WB}}^{*}\alpha_{a}+s_{a}{{}^{WB}}^{*}\delta_{a})\theta )/(\rho +\sigma ))/\rho \end{array};$$

$$\begin{array}{l} L_{r}{}^{WB}=(-((k_{a}s_{a}{{}^{WB}}^{*}{}^{{}^{2}})/2)+(w_{a}-p_{a})(-1+a+p_{a}-s_{a}{{}^{WB}}^{*}\gamma +\mu q_{f}{{}^{WB}}^{*}-q_{a}{{}^{WB}}^{*}(\beta +\mu ))+\\ \;((-w_{a}+p_{a})(q_{a}{{}^{WB}}^{*}\alpha_{a}+s_{a}{{}^{WB}}^{*}\delta_{a})\theta )/(\rho +\sigma ))/\rho \end{array}.$$

### 5.3 Blockchain establishment by the retailer in the wholesale sales mode (*WR*)

The key to the establishment of blockchain in genuine sales channels lies in the introduction of the relevant costs of blockchain, namely, the fixed cost *F* and unit operating cost *c* of establishing blockchain. Different from the *WN* case in which the brand owner establishes blockchain, in the *WR* case, the retailer introduces blockchain to supervise the sale of counterfeit products, and the retailer bears the relevant costs of blockchain, which affects the optimal decision-making and profit distribution of the supply chain members. These factors affect the implementation effect of blockchain. In this case, the differential game model of the brand, retailer and counterfeiter can be constructed as follows:

$$\underset{q_{a}{}^{WR}(t)}{\max}\{\pi_{b}{}^{WB}=\int_{0}^{+\infty }e^{-\rho \cdot t}[w_{a}\cdot d_{a}{}^{WR}(t)-\frac{1}{2}\eta_{a}\cdot q_{a}{{}^{WR}}^{{}^{2}}(t)-\frac{1}{2}k_{a}\cdot s_{a}{{}^{WR}}^{{}^{2}}(t)]dt\}$$

$$\underset{s_{a}{}^{WR}(t)}{\max}\{\pi_{r}{}^{WR}=\int_{0}^{+\infty }e^{-\rho \cdot t}[(p_{a}-w_{a}-c)\cdot d_{a}{}^{WR}(t)-\frac{1}{2}k_{a}\cdot s_{a}{{}^{WR}}^{{}^{2}}(t)]dt-F\}$$

$$s.t.\{\begin{array}{l} dG_{a}{}^{WR}(t)=(\alpha_{a}q_{a}{}^{WR}(t)+\delta_{a}s_{a}{}^{WR}(t))dt-\sigma G_{a}{}^{WR}(t)dt\\ G_{a}{}^{WR}(0)=G_{0} \end{array}$$

$$\underset{q_{f}{}^{WR}(t)}{\max}\{\pi_{f}{}^{WR}=\int_{0}^{+\infty }e^{-\rho \cdot t}[p_{f}\cdot d_{f}{}^{WR}(t)-\frac{1}{2}\eta_{f}\cdot q_{f}{{}^{WR}}^{{}^{2}}(t)]dt\}$$

$$s.t.dG_{f}{}^{WR}(t)=\alpha_{f}q_{f}{}^{WR}(t)dt-\sigma G_{f}{}^{WR}(t)dt,G_{f}{}^{WR}(0)=G_{0}$$

#### Theorem 5

In the case of WR, the brand owner in the wholesale sales mode establishes blockchain, and the optimal product quality of the brand owner and the optimal service level of the retailer are:

$$q_{a}{{}^{WR}}^{*}=\frac{\left(w_{a}-c)(\alpha_{a}\theta +(\beta +\mu )(\rho +\sigma )\right)}{\eta_{a}(\rho +\sigma )}$$ (50)

$$s_{a}{{}^{WR}}^{*}=\frac{\left(p_{a}-w_{a}-c)(\gamma (\rho +\sigma )+\delta_{a}\theta \right)}{k_{a}(\rho +\sigma )}$$ (51)

The product quality determined by the counterfeiter is:

$$q_{f}{{}^{WR}}^{*}=\frac{p_{f}}{\eta_{f}}\cdot (\frac{\alpha_{f}\theta }{\rho +\sigma }+\beta +\mu )$$ (52)

#### Corollary 5

In the case of WR, the optimal dynamic trajectories of genuine and counterfeit brand goodwill are:

$$G_{a}{{}^{WR}}^{*}(t)=(q_{a}{{}^{WR}}^{*}\alpha_{a}+s_{a}{{}^{WR}}^{*}\delta_{a}-e^{-\sigma \cdot t}(q_{a}{{}^{WR}}^{*}\alpha_{a}+s_{a}{{}^{WR}}^{*}\delta_{a}-G_{0}\sigma ))/\sigma$$ (53)

$$G_{f}{{}^{WR}}^{*}(t)=e^{-\sigma \cdot t}(G_{0}-\frac{q_{f}{{}^{WR}}^{*}\alpha_{f}}{\sigma })+\frac{q_{f}{{}^{WR}}^{*}\alpha_{f}}{\sigma }$$ (54)

The optimal dynamic trajectories of the sales quantities of genuine and counterfeit products are:

$$\begin{array}{l} d_{a}{{}^{WR}}^{*}(t)=1-a-p_{a}+q_{a}{{}^{WR}}^{*}\beta +s_{a}{{}^{WR}}^{*}\gamma +(q_{a}{{}^{WR}}^{*}-q_{f}{{}^{WR}}^{*})\mu +e^{-\sigma \cdot t}\theta (G_{0}-(q_{a}{{}^{WR}}^{*}\alpha_{a}+\\ \;s_{a}{{}^{WR}}^{*}\delta_{a})/\sigma )+((q_{a}{{}^{WR}}^{*}\alpha_{a}+s_{a}{{}^{WR}}^{*}\delta_{a})\theta )/\sigma \end{array}$$ (55)

$$\begin{array}{l} d_{f}{{}^{WR}}^{*}(t)=a-p_{f}+q_{f}{{}^{WR}}^{*}\beta +u(-q_{a}{{}^{WR}}^{*}+q_{f}{{}^{WR}}^{*})+\theta e^{-\sigma \cdot t}(G_{0}-(q_{f}{{}^{WR}}^{*}\alpha_{f})/\sigma )+(\\ \;q_{f}{{}^{WR}}^{*}\alpha_{f}\theta )/\sigma \end{array}$$ (56)

The optimal dynamic trajectories of the expected discounted profits for the brand owner, retailer and counterfeiter are:

$$\begin{array}{l} V_{b}{{}^{WB}}^{*}(t)=L_{b}{}^{DB}+(-c+w_{a})\theta (q_{a}{{}^{WR}}^{*}\alpha_{a}+s_{a}{{}^{WR}}^{*}\delta_{a}-e^{-\sigma \cdot t}(q_{a}{{}^{WR}}^{*}\alpha_{a}+s_{a}{{}^{WR}}^{*}\delta_{a}-G_{0}\sigma )\\ \;)/(\sigma (\rho +\sigma )) \end{array}$$ (57)

$$\begin{array}{l} V_{r}{{}^{WR}}^{*}(t)=L_{r}{}^{WR}+(-w_{a}+p_{a})\theta (q_{a}{{}^{WR}}^{*}\alpha_{a}+s_{a}{{}^{WR}}^{*}\delta_{a}-e^{-\sigma \cdot t}(q_{a}{{}^{WR}}^{*}\alpha_{a}+s_{a}{{}^{WR}}^{*}\delta_{a}-G_{0}\sigma \\ \;))/(\sigma (\rho +\sigma )) \end{array}$$ (58)

$$V_{f}{{}^{WR}}^{*}(t)=L_{f}{}^{WR}+\frac{e^{-\sigma \cdot t}(p_{f}\theta ((-1+e^{\sigma \cdot t})q_{f}{{}^{WR}}^{*}\alpha f+G_{0}\sigma ))}{\sigma (\rho +\sigma )}$$ (59)

$$where\;\begin{array}{l} L_{f}{}^{WR}=(-((q_{f}{{}^{WR}}^{*}{}^{{}^{2}}\eta_{f})/2)+p_{f}(a-p_{f}-q_{a}{{}^{WR}}^{*}\mu +q_{f}{{}^{WR}}^{*}(\beta +\mu ))+(p_{f}q_{f}{{}^{WR}}^{*}\alpha_{f}\theta )/(\\ \;\rho +\sigma ))/\rho \end{array};$$

$$\begin{array}{l} L_{b}{}^{WR}=(-(q_{a}{{}^{WR}}^{*}{}^{{}^{2}}\eta_{a})/2+w_{a}(1-a-p_{a}+s_{a}{{}^{WR}}^{*}\gamma -q_{f}{{}^{WR}}^{*}\mu +q_{a}{{}^{WR}}^{*}(\beta +\mu )+((q_{a}{{}^{WR}}^{*}\alpha_{a}+s_{a}{{}^{WR}}^{*}\delta_{a})\theta )\\ \;/(\rho +\sigma )))/\rho \end{array};$$

$$\begin{array}{l} L_{r}{}^{WR}=(-((k_{a}s_{a}{{}^{WR}}^{*}{}^{{}^{2}})/2)+(c-p_{a}+w_{a})(-1+a+p_{a}-\gamma s_{a}{{}^{WR}}^{*}+q_{f}{{}^{WR}}^{*}\mu -q_{a}{{}^{WR}}^{*}(\beta +\mu ))-F\rho -((\\ \;c-p_{a}+w_{a})(q_{a}{{}^{WR}}^{*}\alpha_{a}+s_{a}{{}^{WR}}^{*}\delta_{a})\theta )/(\rho +\sigma ))/\rho \end{array}.$$

## 6 Model analysis

According to the abovementioned scenarios, which include two cases of no blockchain (*DN*) and blockchain adoption (*DB*) when the brand owner is employing the direct sales mode and three cases of no blockchain (*WN*), brand owner-established blockchain (*WB*) and the retailer-established blockchain (*WR*) in the brand owner’s wholesale sales mode. This section will systematically study the equilibrium solutions for the brand owner, retailer and counterfeiter in five cases, discusses the effects of model parameters on the optimal solutions in relevant cases, compares the optimal decisions of supply chain members in different cases, and analyzes the optimal dynamic trajectories of supply chain operation strategy in different differential game models. Additionally, we will study the effect of genuine enterprises using blockchain to supervise the sale of counterfeit products in the supply chain in the direct sales mode and the wholesale sales mode. For the convenience of analysis and description, let *i* = *DN*, *WN* indicate case *DN* or case *WN*; *j* = *DB*, *WB*, *WR* indicate case *WN*, case *WB* or case *WR*.

### 6.1 Parameter analysis

According to the optimal solutions for the model in the different cases in Sections 4 and 5, in this section, the impacts of the parameters related to brand goodwill (i.e., *α*_*f*_, *α*_*a*_, *δ*_*a*_, *λ*, *θ*, *σ*), the influencing factors of service and quality on demand (i.e., *β*, *γ*), the cost coefficients of service and quality (i.e., *η*_*f*_, *η*_*a*_, *k*_*a*_), the probability (*ϕ*) that consumers perceive the product to be genuine, the coefficient of the quality reference effect of consumers (*u*) and the unit operating cost of the blockchain (*c*) on the optimal quality and optimal service level of genuine products and the quality of counterfeit products in the relevant cases are investigated, and the results are summarized in Propositions 1 to 3.

#### Proposition 1

In different cases, the impacts of brand goodwill-related parameters (i.e., α_f_, α_a_, δ_a_, λ, θ, σ) on the optimal decisions are analyzed as follows:

- $\frac{\partial q_{f}{{}^{i,j}}^{*}}{\partial \alpha_{f}}>0$, $\frac{\partial q_{f}{{}^{i,j}}^{*}}{\partial \alpha_{a}}=0$, $\frac{\partial q_{f}{{}^{i,j}}^{*}}{\partial \delta_{a}}=0$, $\frac{\partial q_{f}{{}^{i}}^{*}}{\partial \lambda }>0$, $\frac{\partial q_{f}{{}^{i,j}}^{*}}{\partial \theta }>0$, $\frac{\partial q_{f}{{}^{i,j}}^{*}}{\partial \sigma }<0$;
- $\frac{\partial q_{a}{{}^{i,j}}^{*}}{\partial \alpha_{f}}=0$, $\frac{\partial q_{a}{{}^{i,j}}^{*}}{\partial \alpha_{a}}>0$, $\frac{\partial q_{a}{{}^{i,j}}^{*}}{\partial \delta_{a}}=0$, $\frac{\partial q_{a}{{}^{i}}^{*}}{\partial \lambda }<0$, $\frac{\partial q_{a}{{}^{i,j}}^{*}}{\partial \theta }>0$, $\frac{\partial q_{a}{{}^{i,j}}^{*}}{\partial \sigma }<0$;
- $\frac{\partial s_{a}{{}^{i,j}}^{*}}{\partial \alpha_{f}}=0$, $\frac{\partial s_{a}{{}^{i,j}}^{*}}{\partial \alpha_{a}}=0$, $\frac{\partial s_{a}{{}^{i,j}}^{*}}{\partial \delta_{a}}>0$, $\frac{\partial s_{a}{{}^{i}}^{*}}{\partial \lambda }<0$, $\frac{\partial s_{a}{{}^{i,j}}^{*}}{\partial \theta }>0$, $\frac{\partial s_{a}{{}^{i,j}}^{*}}{\partial \sigma }<0$;

where i = DN, WN; j = DB, WB, WR.

The relevant parameters of brand goodwill include the influence factor *α*_*f*_ of the counterfeit product quality on brand goodwill, the influence factor *α*_*a*_ of the genuine product quality on brand goodwill, the influence factor *δ*_*a*_ of the service level of genuine product sales on brand goodwill, the influence degree *λ* of the counterfeit products on brand goodwill, the influence factor *θ* of the brand goodwill on consumer demand, and the natural decline rate *σ* of brand goodwill. Proposition 1 shows that the quality of counterfeit products *q*_*f*_^*i*, j*^ in different cases is positively correlated with parameter *α*_*f*_ but not with parameters *α*_*a*_ and *δ*_*a*_. However, the optimal quality *q*_*a*_^*i*, j*^ of genuine products is positively correlated with parameter *α*_*a*_ but not with parameters *α*_*f*_ and *δ*_*a*_. Moreover, the optimal service level *s*_*a*_^*i*, j*^ of genuine goods is positively correlated with parameter *δ*_*a*_ but not with parameters *α*_*a*_ and *δ*_*a*_. This relationship implies that the influence factor of a certain decision on brand goodwill in the model only positively affects the optimal value of the decision and does not affect other optimal decisions. For example, when the influence factor *α*_*a*_ of the quality of genuine products on brand goodwill increases, brand owners are more motivated to improve the quality of their products to improve their brand goodwill and increase consumers’ demand for genuine products.

In the direct sales mode and wholesale sales mode without blockchain, the quality of counterfeit products *q*_*f*_^i*^ is positively correlated with parameter *λ*, while the optimal quality and service level of genuine products are negatively correlated with parameter *λ* because when the impact of counterfeit products on brand goodwill increases, counterfeiters use that opportunity to improve the quality of their counterfeit products and increase the consumer demand for counterfeit products. However, due to the asymmetry of quality information, the influence of genuine products on brand goodwill is reduced, and the influence of product quality and service level on brand goodwill in genuine channels is weakened, which forces genuine enterprises to reduce their optimal decisions. This result explains that in the absence of blockchain, in practice, the sale of counterfeit products damages the quality and service level of genuine products by affecting brand goodwill, which is often unfavorable to genuine enterprises, and it is easy to form a “lemon market” to harm the interests of consumers.

In addition, in different cases, optimal decisions made by both the counterfeiter and genuine enterprises are positively correlated with parameter *θ* but negatively correlated with parameter *σ*. Intuitively, when consumers pay more attention to brand goodwill, both the counterfeiter and genuine enterprises have the motivation to improve their optimal decision-making to improve their brand reputation, increase the demand from consumers and improve their own profits. In contrast, an increase in the decline rate of brand goodwill usually weakens the impact of relevant decisions on the accumulation of brand goodwill, thus undermining the optimal decisions made by the counterfeiter and genuine enterprises.

#### Proposition 2

In different cases, the influence factors of service and quality on demand (i.e., β, γ) and the cost coefficients of service and quality (i.e., η_f_, η_a_, k_a_), the impacts of these parameters on optimal decisions are analyzed as follows:

- $\frac{\partial q_{f}{{}^{i,j}}^{*}}{\partial \eta_{f}}<0$, $\frac{\partial q_{f}{{}^{i,j}}^{*}}{\partial \eta_{a}}=0$, $\frac{\partial q_{f}{{}^{i,j}}^{*}}{\partial k_{a}}=0$, $\frac{\partial q_{f}{{}^{i,j}}^{*}}{\partial \beta }>0$, $\frac{\partial q_{f}{{}^{i,j}}^{*}}{\partial \gamma }=0$;
- $\frac{\partial q_{a}{{}^{i,j}}^{*}}{\partial \eta_{a}}=0$, $\frac{\partial q_{a}{{}^{i,j}}^{*}}{\partial \eta_{a}}<0$, $\frac{\partial q_{a}{{}^{i,j}}^{*}}{\partial k_{a}}=0$, $\frac{\partial q_{a}{{}^{i,j}}^{*}}{\partial \beta }>0$, $\frac{\partial q_{a}{{}^{i,j}}^{*}}{\partial \gamma }=0$;
- $\frac{\partial s_{a}{{}^{i,j}}^{*}}{\partial \eta_{a}}=0$, $\frac{\partial s_{a}{{}^{i,j}}^{*}}{\partial \eta_{a}}<0$, $\frac{\partial s_{a}{{}^{i,j}}^{*}}{\partial k_{a}}<0$, $\frac{\partial s_{a}{{}^{i,j}}^{*}}{\partial \beta }=0$, $\frac{\partial s_{a}{{}^{i,j}}^{*}}{\partial \gamma }>0$;

where i = DN, WN; j = DB, WB, WR.

Proposition 2 reveals that the cost coefficients *η*_*f*_, *η*_*a*_ and *k*_*a*_ negatively affect the optimal quality of counterfeit products *q*_*f*_^*i*, j*^, the optimal quality of genuine products *q*_*a*_^*i*, j*^ and the service level *s*_*a*_^*i*, j*^ under different cases, respectively, because intuitively, when the cost coefficient of a quality or service increases, the marginal cost incurred by the optimal decision increases, and both the counterfeiter and genuine enterprises will appropriately reduce the product quality and service level to avoid excessive costs. In addition, the optimal quality of counterfeit products *q*_*f*_^*i*, j*^ and the optimal quality of genuine products *q*_*a*_^*i*, j*^ in different cases are positively correlated with the influence factor *β* of quality on demand but not with the optimal service level *s*_*a*_^*i*, j*^ of genuine products. However, the optimal service level *s*_*a*_^*i*, j*^ of genuine products is positively correlated with the influence factor *γ* of service on demand but has nothing to do with the optimal quality *q*_*f*_^*i*, j*^ of counterfeit products and the optimal quality *q*_*a*_^*i*, j*^ of genuine products. This phenomenon implies that when consumers are more sensitive to the quality of products, the brand owner and counterfeiter will use that opportunity to improve the quality of their products, increase consumer demand, and improve their profits. Similarly, when consumers pay more attention to their service experience in stores, the service level of genuine sales will also be improved to meet the needs of consumers and improve the profits for genuine enterprises.

#### Proposition 3

In different cases, the probability ϕ that consumers believe that the product is genuine, the consumer’s quality reference effect coefficient u and the unit operating cost c, the impacts of these parameters on optimal decisions are analyzed as follows:

- $\frac{\partial q_{f}{{}^{i}}^{*}}{\partial \phi }<0$, $\frac{\partial q_{f}{{}^{j}}^{*}}{\partial u}>0$, $\frac{\partial q_{f}{{}^{j}}^{*}}{\partial c}=0$;
- $\frac{\partial q_{a}{{}^{i}}^{*}}{\partial \phi }>0$, $\frac{\partial q_{a}{{}^{j}}^{*}}{\partial u}>0$, $\frac{\partial q_{a}{{}^{DB,WB}}^{*}}{\partial c}<0$;
- $\frac{\partial s_{a}{{}^{i}}^{*}}{\partial \phi }=0$, $\frac{\partial s_{a}{{}^{j}}^{*}}{\partial u}=0$, $\frac{\partial s_{a}{{}^{DB,WR}}^{*}}{\partial c}<0$; *where i = DN*, *WN; j = DB*, *WB*, *WR*.

According to Proposition 3, in the absence of blockchain, the asymmetry of product quality information affects the optimal decision-making regarding genuine product quality and counterfeit product quality. Specifically, the optimal quality of counterfeit products *q*_*f*_^i*^ is negatively correlated with the probability *ϕ* of genuine products, while the optimal quality of genuine products *q*_*a*_^i*^ is positively correlated; however, the optimal service level of genuine products *s*_*a*_^j*^ is irrelevant. This phenomenon implies that when consumers believe that the product is more likely to be counterfeited (i.e., the smaller *ϕ*), the brand owner loses confidence and reduces the quality of genuine products because the impact of the quality of genuine products on the perceived product quality *Q* ^i*^ = *ϕ* · *q*_*a*_^i*^+(1-*ϕ*) · *q*_*f*_^i*^ is reduced; additionally, the brand owner has no choice but to reduce its product quality to balance revenue and cost. It can be seen that for brand owners, when the quality information is more asymmetric, that is, the more *ϕ* deviates from 1, the more unfavorable it is in terms of product quality improvement.

Under blockchain technology, the quality reference effect coefficient *u* of consumers has a positive impact on the optimal quality *q*_*f*_^j*^ of counterfeit products and the optimal quality *q*_*a*_^j*^ of genuine products but does not affect the optimal service level *s*_*a*_^j*^ of genuine products, which means that blockchain technology helps consumers distinguish between genuine and counterfeit products. When the consumer quality reference effect is enhanced, both brand owners and counterfeiters improve the quality of their products because the brand owner wants to improve the quality *q*_*a*_^j*^ of genuine products to widen its difference with the quality *q*_*f*_^j*^ of counterfeit products, that is, to increase *u*·(*q*_*a*_^j*^- *q*_*f*_^j*^), to improve consumers’ demand for genuine products. In contrast, the counterfeiter tends to reduce the difference between the quality *q*_*f*_^j*^of counterfeit products and the quality *q*_*a*_^j*^ of genuine products, that is, they reduce *u*·(*q*_*a*_^j*^- *q*_*f*_^j*^) to increase consumers’ demand for counterfeit products.

In addition, Proposition 3 intuitively shows that the optimal quality *q*_*f*_^j*^ of counterfeit products is not affected by the unit operating cost *c* of blockchain. However, when blockchain is adopted by a brand owner in the direct sales mode, the optimal quality *q*_*a*_^DB*^ and service level *s*_*a*_^DB*^ of genuine products are negatively correlated with *c*. In the wholesale sales mode, when blockchain is established by the brand owner, the optimal quality *q*_*a*_^WB*^ of genuine products is negatively correlated with *c*, while the optimal service level *s*_*a*_^WB*^ of genuine products is not related to *c*. When blockchain is established by a retailer employing the wholesale sales mode, the optimal quality *q*_*a*_^WB*^ of genuine products has nothing to do with *c*, while the optimal service level *s*_*a*_^WB*^ of genuine products has a negative correlation with *c*. This phenomenon indicates that the impact of the blockchain unit operating cost on optimal decisions for genuine products is related not only to the sales modes of the brand owner but also to the genuine enterprises that introduce and establish blockchain. In addition, the blockchain unit operating cost is generally unfavorable to decision-making for genuine products, which also causes genuine enterprises to weigh the advantages and disadvantages when deciding to adopt blockchain.

### 6.2 Comparative analysis of optimal decisions

This section compares and analyzes the quality of counterfeit products and the optimal quality and service level of genuine products under different cases and examines the impact of the brand owner’s sales mode shifts and blockchain implementation on the optimal decisions of counterfeit companies and genuine enterprises.

#### Proposition 4

In the direct sales mode, the comparative analyses of the optimal decisions when there is no blockchain (DN case) and blockchain adoption (DB case) are as follows:

q_f_^DB*^>q_f_^DN*^,$\left\{ \begin{array}{l} if\;0<\frac{c}{p_{a}}\leq f(\cdot ),\;then\;q_{a}{{}^{DB}}^{*}\geq q_{a}{{}^{DN}}^{*}\\ if\;f(\cdot )<\frac{c}{p_{a}},\;then\;q_{a}{{}^{DB}}^{*}<q_{a}{{}^{DN}}^{*} \end{array}\right.$, $\left\{ \begin{array}{l} if\;0<\frac{c}{p_{a}}\leq g(\cdot ),\;then\;s_{a}{{}^{DB}}^{*}\geq s_{a}{{}^{DN}}^{*}\\ if\;g(\cdot )<\frac{c}{p_{a}},\;then\;s_{a}{{}^{DB}}^{*}<s_{a}{{}^{DN}}^{*} \end{array}\right.$,

where f(·)=$\frac{\alpha_{a}\theta \lambda +(\beta +u-\phi \beta )(\rho +\sigma )}{\alpha_{a}\theta +(\beta +u)(\rho +\sigma )}$ and g(·) = $\frac{\delta_{a}\theta \lambda }{\delta_{a}\theta +\gamma (\rho +\sigma )}$.

Proposition 4 shows that when the brand owner is in the direct sales mode, the quality of counterfeit products under blockchain technology is higher than that without blockchain, that is, *q*_*f*_^DB*^ > *q*_*f*_^DN*^. This relationship implies that a brand owner’s adoption of blockchain forces counterfeiters to improve the quality of their products because consumers can identify genuine and counterfeit products by using blockchain. If counterfeiters provide consumers with inferior products, the consumer demand for counterfeit products will be greatly reduced, and the profits of counterfeiters will be severely affected. Proposition 4 also states that for the brand owner, when the ratio *c*/*p*_*a*_ of parameters *c* and *p*_*a*_ is small, the adoption of blockchain improves the optimal quality and service level of genuine products, that is, *q*_*a*_^DB*^ > *q*_*a*_^DN*^, *s*_*a*_^DB*^ > *s*_*a*_^DN*^. When the ratio of *c*/*p*_*a*_ is large, the result is the opposite. It can be seen that the implementation of blockchain by brand owners does not improve the optimal quality and service level of their genuine products, which depends on the unit operating cost *c* of blockchain and the sales price *p*_*a*_ of authentic products. This finding is interesting and suggests that when the blockchain unit operating cost is small and the sales price of genuine products is high, brand owners’ investment in blockchain improves the optimal quality and service level of their genuine products; otherwise, it may reduce the optimal quality and service level of their genuine products.

#### Proposition 5

In the wholesale sales mode, the comparative analyses of the optimal decisions when there is no blockchain (DN case) and blockchain adoption (WB and WR cases) are as follows:

(i) q_f_^WB*^> q_f_^WN*^, $\left\{ \begin{array}{l} if\;0<\frac{c}{w_{a}}\leq f(\cdot ),\;then\;q_{a}{{}^{WB}}^{*}\geq q_{a}{{}^{WN}}^{*}\\ if\;f(\cdot )<\frac{c}{w_{a}},\;then\;q_{a}{{}^{WB}}^{*}<q_{a}{{}^{WN}}^{*} \end{array}\right.$, s_a_^WB*^ > s_a_^WN*^;

(ii) q_f_^WR*^> q_f_^WN*^, q_a_^WR*^> q_a_^WN*^, $\left\{ \begin{array}{l} if\;0<\frac{c}{p_{a}-w_{a}}\leq g(\cdot ),\;then\;s_{a}{{}^{WR}}^{*}\geq s_{a}{{}^{WN}}^{*}\\ if\;g(\cdot )<\frac{c}{p_{a}-w_{a}},\;then\;s_{a}{{}^{WR}}^{*}<s_{a}{{}^{WN}}^{*} \end{array}\right.$

where f(·) = $\frac{\alpha_{a}\theta \lambda +(\beta +u-\phi \beta )(\rho +\sigma )}{\alpha_{a}\theta +(\beta +u)(\rho +\sigma )}$ and g(·) = $\frac{\delta_{a}\theta \lambda }{\delta_{a}\theta +\gamma (\rho +\sigma )}$.

From Proposition 5, it can be seen that even when the brand owner is in a wholesale sales mode, the quality of counterfeit products under blockchain technology is higher than that without blockchain, that is, *q*_*f*_^WB*^>*q*_*f*_^WN*^, *q*_*f*_^WR*^>*q*_*f*_^WN*^, which is similar to the direct sales mode in Proposition 4. However, whether a brand owner’s adoption of blockchain will improve the optimal quality of genuine products depends on the ratio *c*/*w*_*a*_ of the blockchain unit operating cost *c* and the wholesale price *w*_*a*_ of genuine products, and the optimal service level under blockchain is higher than that without blockchain. The difference is that the blockchain established by the retailer will improve the optimal quality of genuine products, and the improvement of the optimal service level depends on the ratio *c*/(*p*_*a*_ -*w*_*a*_) of the blockchain unit operating cost *c* and the retailer’s marginal revenue (*p*_*a*_-*w*_*a*_). This phenomenon indicates that the impact of the introduction of blockchain by the brand owner or retailer on the decision-making regarding the optimal quality and service level of genuine products is different because the party implementing the blockchain needs to weigh the unit operating cost and marginal income of the blockchain to optimize the decision-making. The party participating in blockchain will improve its optimal decision to enhance the impact of genuine products’ quality or service on brand goodwill and improve brand image.

#### Proposition 6

In the wholesale sales mode, the comparative analyses of the optimal decisions when the brand owner establishes the blockchain (WB case) and the retailer establishes the blockchain (WR case) are as follows: q_f_^WB*^ = q_f_^WB*^, q_a_^WB*^< q_a_^WR*^, s_a_^WB*^>s_a_^WR*^.

Proposition 6 reveals that when the brand owner is in the wholesale sales mode, the quality decision for counterfeit products is the same in the case of two blockchain adoptions, but the optimal quality and service level of genuine products are not the same. When the blockchain is established by the retailer, the optimal quality of genuine products is higher, while when the blockchain is established by the brand owner, the optimal service level of genuine products is higher. This finding is nonintuitive and implies that the adoption of blockchain by the brand owner or retailer has the same impact and effect on the decision-making for counterfeit products because blockchain has the same effect on supervising counterfeiters to forge genuine products. However, a brand owner establishes the blockchain differently from how a retailer establishes the blockchain in terms of the decisions about the optimal quality and service level of genuine products. The reason is similar to the analysis in Proposition 5; that is, the party implementing blockchain needs to reduce its optimal decision to reduce costs and balance the blockchain unit operating cost to maximize its profit.

#### Proposition 7

When there is no blockchain, the comparative analyses of the optimal decisions in the direct sales mode (DN case) and wholesale sales mode (WN case) are as follows: q_f_^DN*^ = q_f_^WN*^, q_a_^DN*^>q_a_^WN*^, s_a_^DN*^>s_a_^WN*^.

Proposition 7 shows the impact of the brand owner’s choice of different sales modes on the optimal quality of counterfeit products and the optimal quality and service level of genuine products in the absence of blockchain. The results identify that a brand owner’s choice to employ a direct sales mode or wholesale sales mode does not affect quality decisions for counterfeiters but affects the optimal decisions for genuine products. Specifically, the optimal quality and service level of genuine products are higher in the direct sales mode than in the wholesale sales mode, which means that the brand owner’s choice of sales modes will not influence the improvement of the quality of fake and inferior products by counterfeiters. However, brand owners can provide consumers with a better product quality and service level under the direct selling mode, which is consistent with reality. In particular, some large brand owners (such as Louis Vuitton) rely more on selling through their own direct franchised stores, which often helps to improve their product quality and service and enhance their brand image.

#### Proposition 8

When blockchain is adopted, the comparative analyses of the optimal decisions in the direct sales mode (DN case) and wholesale sales mode (WB and WR cases) are as follows:

(i) q_f_^DB*^ = q_f_^WB*^, q_a_^DB*^>q_a_^WB*^, s_a_^DB*^>s_a_^WB*^;

*(ii) q*_*f*_^DB*^ *= q*_*f*_^WR*^, *q*_*a*_^DB*^>q_*a*_^WR*^, *s*_*a*_^DB*^>s_a_^WR*^.

According to Proposition 8, it can be seen that when blockchain is adopted by a brand owner using different sales modes, the quality of counterfeit products is the same, while the optimal quality and service level of genuine products are higher in the direct sales mode than in the wholesale sales mode. This finding is similar to Proposition 7. It also shows that even under blockchain technology, the direct sales mode is still more advantageous than the wholesale sales mode in terms of improving the quality and service of genuine products.

### 6.3 Optimal dynamic trajectory analysis of supply chain operation strategy

The optimal dynamic trajectories of brand goodwill, consumer demand for genuine and counterfeit products, and expected discounted profits of relevant enterprises under the equilibrium decisions for the five cases of *DN*, *DB*, *WN*, *WB* and *WR*, are studied in this section to provide a reference and theoretical basis for relevant decision-makers in genuine enterprises using different sales modes to optimize their brand image, sales, profits, and other important operational indicators before and after the adoption of blockchain.

#### Proposition 9

When there is no blockchain, the optimal dynamic trajectories of the supply chain operation strategy in the direct sales mode (DN case) and wholesale sales mode (WN case) are as follows:

(i) When t < t_th_^i^,

$$\left\{ \begin{array}{l} if\;G_{0}\leq T_{2}{}^{i},\;then\frac{\partial G^{i}}{\partial t}>0,\frac{\partial d_{f}{}^{i}}{\partial t}>0,\frac{\partial d_{a}{}^{i}}{\partial t}>0,\frac{\partial V_{f}{}^{i}}{\partial t}>0,\frac{\partial V_{a}{}^{i}}{\partial t}>0,\frac{\partial V_{r}{}^{WN}}{\partial t}>0\\ if\;G_{0}>T_{2}{}^{i}\;and\;p_{a}\leq \frac{\left(G_{0}-T_{2}{}^{i}\right)}{T_{1}{}^{i}},\;then\frac{\partial G^{i}}{\partial t}\leq 0,\frac{\partial d_{f}{}^{i}}{\partial t}\leq 0,\frac{\partial d_{a}{}^{i}}{\partial t}\leq 0,\frac{\partial V_{f}{}^{i}}{\partial t}\leq 0,\frac{\partial V_{a}{}^{i}}{\partial t}\leq 0,\frac{\partial V_{r}{}^{WN}}{\partial t}\leq 0\\ if\;G_{0}>T_{2}{}^{i}\;and\;p_{a}>\frac{\left(G_{0}-T_{2}{}^{i}\right)}{T_{1}{}^{i}}\;then\frac{\partial G^{i}}{\partial t}>0,\frac{\partial d_{f}{}^{i}}{\partial t}>0,\frac{\partial d_{a}{}^{i}}{\partial t}>0,\frac{\partial V_{f}{}^{i}}{\partial t}>0,\frac{\partial V_{a}{}^{i}}{\partial t}>0,\frac{\partial V_{r}{}^{WN}}{\partial t}>0 \end{array};\right.$$

(ii) When t ≥ t_th_^i^, $\frac{\partial G^{i}}{\partial t}=0$, $\frac{\partial d_{f}{}^{i}}{\partial t}=0$, $\frac{\partial d_{a}{}^{i}}{\partial t}=0$, $\frac{\partial V_{f}{}^{i}}{\partial t}=0,\frac{\partial V_{a}{}^{i}}{\partial t}=0,$ $\frac{\partial V_{r}{}^{WN}}{\partial t}=0$;

where i=DN, WN; t_th_^i^ is the time threshold in (0, +∞);

$$T_{1}{}^{DN}=\frac{1}{k_{a}\eta_{a}\sigma (\rho +\sigma )}[(1-\lambda )(\delta_{a}\eta_{a}(\delta_{a}(\theta -\theta \lambda )+\gamma (\rho +\sigma ))+k_{a}\alpha_{a}(\alpha_{a}(\theta -\theta \lambda )+\beta (\rho +\sigma )\phi ))];$$

$$T_{2}{}^{DN}=(p_{f}\alpha_{f}\lambda (\alpha_{f}\theta \lambda +\beta (\rho +\sigma )(1-\phi )))/(\eta_{f}\sigma (\rho +\sigma ));$$

$$T_{1}{}^{WN}=(\delta_{a}(1-\lambda )(\delta_{a}\theta (1-\lambda )+\gamma (\rho +\sigma )))/(k_{a}\sigma (\rho +\sigma ));$$

$$\begin{array}{l} T_{2}{}^{WN}=\frac{1}{\eta_{a}\eta_{f}k_{a}\sigma (\rho +\sigma )}(\delta_{a}\eta_{a}\eta_{f}(1-\lambda )w_{a}(\gamma (\rho +\sigma )+\delta_{a}\theta (1-\lambda ))-k_{a}(\alpha_{f}\eta_{a}\lambda p_{f}(\alpha_{f}\theta \lambda +\beta (1\\ \;-\phi )(\rho +\sigma ))+\alpha_{a}\eta_{f}(1-\lambda )w_{a}(\alpha_{a}(\theta -\theta \lambda )+\beta \phi (\rho +\sigma )))) \end{array}.$$

Proposition 9 provides the key insight that in the direct sales mode and wholesale sales mode without blockchain, the optimal dynamic trajectories of the operation strategy for the counterfeiter and genuine enterprises are related to the operation time *t*, the initial value of brand goodwill *G*_0_ and the genuine sales price *p*_*a*_, and they have a consistent change rule. Specifically, Proposition 9 (i) shows that in the early stage of enterprise operations, that is, *t*<*t*_*th*_^*i*^, if the initial value of brand goodwill *G*_0_ is low, the optimal dynamic trajectories of the supply chain operation strategy will monotonously increase over time to gradually improve the brand image, increase consumer demand and improve the profitability of the enterprises.

However, if the initial value of brand goodwill *G*_0_ is high and the sales price *p*_*a*_ of genuine products is small, the optimal dynamic trajectories of the supply chain operation strategy monotonically decrease with time. When the initial value of brand goodwill *G*_0_ is high and the sales price *p*_*a*_ of genuine products is large, the result is the opposite. This phenomenon implies that when the brand product has a high initial goodwill, if the sales price of the genuine enterprise is small, the product quality will be low, the brand goodwill will be gradually reduced, and the consumer demand and corporate profit will also be reduced. If the sales price of the genuine enterprise is high, the product quality can be improved, the brand goodwill will be improved, and the consumer demand and corporate profit will also be improved. Proposition 9 (*i*) also shows that the critical points affecting the change in supply chain operation strategy under different sales modes are not the same, and the counterfeiter relies on the brand goodwill of genuine enterprises and seeks profit when there is no blockchain. Proposition 9 (ii) shows that when the operation time of an enterprise exceeds a critical point, that is, *t* ≥ *t*_*th*_^*i*^, the optimal dynamic trajectories of the supply chain operation strategy will not change with time to realize the steady-state operation of relevant enterprises in the market.

#### Proposition 10

When blockchain is adopted, the optimal dynamic trajectories of the supply chain operation strategy in the direct sales mode (DB case) and the wholesale sales mode (WB and WR cases) are as follows:

(i) when t < t_tha_^j^,

$\left\{ \begin{array}{l} if\;G_{0}\geq T_{2}{}^{j},\;then\frac{\partial G_{a}{}^{j}}{\partial t}<0,\frac{\partial d_{a}{}^{j}}{\partial t}<0,\frac{\partial V_{a}{}^{j}}{\partial t}<0,\frac{\partial V_{r}{}^{k}}{\partial t}<0\\ if\;G_{0}<T_{2}{}^{j}\;and\;c\leq \frac{\left(T_{2}{}^{j}-G_{0}\right)}{T_{1}{}^{j}},\;then\frac{\partial G_{a}{}^{j}}{\partial t}\geq 0,\frac{\partial d_{a}{}^{j}}{\partial t}\geq 0,\frac{\partial V_{a}{}^{j}}{\partial t}\geq 0,\frac{\partial V_{r}{}^{k}}{\partial t}\geq 0\\ if\;G_{0}<T_{2}{}^{j}\;and\;c>\frac{\left(T_{2}{}^{j}-G_{0}\right)}{T_{1}{}^{j}},\;then\frac{\partial G_{a}{}^{j}}{\partial t}<0,\frac{\partial d_{a}{}^{j}}{\partial t}<0,\frac{\partial V_{a}{}^{j}}{\partial t}<0,\frac{\partial V_{r}{}^{k}}{\partial t}<0 \end{array};\right.$

(ii) when t ≥ t_tha_^j^, $\frac{\partial G_{a}{}^{j}}{\partial t}=0,\frac{\partial d_{a}{}^{j}}{\partial t}=0,\frac{\partial V_{a}{}^{j}}{\partial t}=0$;

(iii) when t < t_thf_^j^,

$\left\{ \begin{array}{l} if\;p_{f}\leq \frac{G_{0}\eta_{f}\sigma (\rho +\sigma )}{\alpha_{f}(\alpha_{f}\theta +(\beta +\mu )(\rho +\sigma ))},\;then\frac{\partial G_{f}{}^{j}}{\partial t}\leq 0,\frac{\partial d_{f}{}^{j}}{\partial t}\leq 0,\frac{\partial V_{f}{}^{j}}{\partial t}\leq 0\\ if\;p_{f}>\frac{G_{0}\eta_{f}\sigma (\rho +\sigma )}{\alpha_{f}(\alpha_{f}\theta +(\beta +\mu )(\rho +\sigma ))},\;then\frac{\partial G_{f}{}^{j}}{\partial t}>0,\frac{\partial d_{f}{}^{j}}{\partial t}>0,\frac{\partial V_{f}{}^{j}}{\partial t}>0 \end{array}\right.$;

(iv) when t ≥ t_thf_^j^, $\frac{\partial G_{f}{}^{j}}{\partial t}=0,\frac{\partial d_{f}{}^{j}}{\partial t}=0,\frac{\partial V_{f}{}^{j}}{\partial t}=0$;

where k=WB, WR; j= DB, WB, WR. t_tha_^j^ and t_thf_^j^ are the time thresholds in (0, +∞);

$$T_{1}{}^{DB}=(\delta_{a}\eta_{a}(\delta_{a}\theta +\gamma (\rho +\sigma ))+k_{a}\alpha_{a}(\alpha_{a}\theta +(\beta +\mu )(\rho +\sigma )))/(k_{a}\eta_{a}\sigma (\rho +\sigma ));$$

$$T_{2}{}^{DB}=(p_{a}\delta_{a}\eta_{a}(\delta_{a}\theta +\gamma (\rho +\sigma ))+k_{a}p_{a}\alpha_{a}(\alpha_{a}\theta +(\beta +\mu )(\rho +\sigma )))/(k_{a}\eta_{a}\sigma (\rho +\sigma ));$$

$$T_{1}{}^{WB}=\alpha_{a}(\alpha_{a}\theta +(\beta +\mu )(\rho +\sigma ))/(\eta_{a}\sigma (\rho +\sigma ));$$

$$T_{2}{}^{WB}=\frac{1}{\left(k_{a}\eta_{a}\sigma (\rho +\sigma )\right)}[((p_{a}-w_{a})\delta_{a}\eta_{a}(\delta_{a}\theta +\gamma (\rho +\sigma ))+k_{a}w_{a}\alpha_{a}(\alpha_{a}\theta +(\beta +\mu )(\rho +\sigma )))];$$

$$T_{1}{}^{WR}=(\delta_{a}(\delta_{a}\theta +\gamma (\rho +\sigma )))/(k_{a}\sigma (\rho +\sigma ));$$

$$T_{2}{}^{WR}=\frac{1}{\eta_{a}k_{a}\sigma (\rho +\sigma )}[\alpha_{a}k_{a}w_{a}(\alpha_{a}\theta +(\beta +\mu )(\rho +\sigma ))+\delta_{a}\eta_{a}(p_{a}-w_{a})(\gamma (\rho +\sigma )+\delta_{a}\theta )].$$

Proposition 10 presents an interesting new insight: the optimal dynamic trajectories of the supply chain operation strategy after the adoption of blockchain are different from those without blockchain. The differences from Proposition 9 are as follows: (1) Under blockchain technology, the critical conditions that affect the optimal dynamic trajectories of the operation strategy adopted by both the counterfeiter and genuine enterprises are different and independent of each other, which implies that blockchain distinguishes genuine products from counterfeit products and eliminates the negative impact of the sale of counterfeit products on the brand image, sales quantity and profits of a genuine enterprise operation strategy. (2) The optimal dynamic trajectories of the operation strategy of genuine enterprises under blockchain technology are different from those without blockchain. This phenomenon is mainly reflected in the early stage of enterprise operation, that is, *t* < *t*_*tha*_^*j*^. If the initial value of brand goodwill *G*_0_ is high, the optimal dynamic trajectories of the operation strategy of genuine enterprises are decreasing functions of time. However, if the initial value of brand goodwill *G*_0_ is low, the optimal dynamic trajectories of the operation strategy chosen by genuine enterprises depend on the unit operation cost *c* of the blockchain. Especially when the unit operation cost *c* of the blockchain is large, the optimal dynamic trajectories of the operation strategy chosen by genuine enterprises will still monotonously decrease over time. It can be seen that if the implementation of blockchain incurs higher unit operating costs, the operation status of genuine enterprises may deteriorate. (3) For the counterfeiter, the optimal dynamic trajectories of its operation strategy are related to the operation time *t* and the sales price *p*_*f*_ of counterfeit products. According to Proposition 10 (iii), in the early stage of the counterfeiter’s operation, that is, *t* < *t*_*thf*_^*j*^, if the sales price *p*_*f*_ is small, the optimal dynamic trajectories of the counterfeiter’s operation strategy are decreasing functions of time; if the sales price *p*_*f*_ is large, the result is the opposite. This phenomenon means that under blockchain technology, if counterfeiters choose to produce inferior products at a lower price to pursue the low-end market, their operation status will worsen; if counterfeiters choose to improve the quality of their products at a higher price to pursue a high-quality market, their operation status will continue to improve. Similar to Proposition 9, under blockchain technology, the optimal dynamic trajectories of the respective operation strategies eventually tend to become stable over time.

### 6.4 Effect of blockchain technology in supervising counterfeit products

The adoption of blockchain by genuine enterprises can eliminate the asymmetry of product quality information, identify genuine and counterfeit products for consumers, and thus allow for the supervision of the sale of counterfeit products. However, whether to adopt blockchain ultimately depends on whether the implementation of blockchain can reduce the counterfeiter’s profit and improve the profits for genuine enterprises. This section studies the impact of blockchain adoption on the profits of both counterfeit and genuine enterprises to analyze the supervisory effect of supply chain counterfeit products when the brand owner employs the direct sales mode or wholesale sales mode under blockchain technology. It can be seen from Section 6.3 that from the perspective of the long-term operation of enterprises, the expected discounted profits of supply chain members tend to be stable. Therefore, to continuously and effectively supervise the sales of counterfeit products in the long term, this section analyzes the expected discounted profit for supply chain members in the steady state and obtains Propositions 11–13.

#### Proposition 11

In the direct sales mode, the effect of blockchain on the supervision of counterfeit products in the supply chain is expressed as follows:

(i) $\left\{ \begin{array}{l} if\;c\leq c_{th}{}^{DB},\;then\;V_{f}{}^{DB}\leq V_{f}{}^{DN}\\ if\;c>c_{th}{}^{DB},\;then\;V_{f}{}^{DB}>V_{f}{}^{DN} \end{array}\right.$; (ii) $\left\{ \begin{array}{l} if\;F\leq F_{th}{}^{DB},\;then\;V_{b}{}^{DB}\geq V_{b}{}^{DN}\\ if\;F>F_{th}{}^{DB},\;then\;V_{b}{}^{DB}<V_{b}{}^{DN} \end{array}\right.$;

where

$$\begin{array}{l} c_{th}{}^{DB}=((2p_{a}\delta_{a}\eta_{a}\eta_{f}\theta (1-\lambda )(\rho +\sigma )(\delta_{a}\theta (1-\lambda )+\gamma (\rho +\sigma ))+k_{a}(2p_{a}\eta_{f}(\rho +\sigma )(\alpha_{a}{}^{2}\theta^{2}{\left(1-\lambda \right)}^{2}+\\ \;\alpha_{a}\theta (\mu \sigma +\beta (1-\lambda )(\rho +2\sigma )\phi )+\sigma (\rho +\sigma )(\mu (\beta +\mu )+\beta^{2}\phi^{2}))))-k_{a}(p_{f}\eta_{a}(\alpha_{f}{}^{2}\theta^{2}(1-\lambda^{2}\\ \;)(2\rho +\sigma )+2\alpha_{f}\theta {\left(\rho +\sigma \right)}^{2}(\mu +\beta (1-\lambda (1-\phi )))+\sigma {\left(\rho +\sigma \right)}^{2}(\mu +\beta (2-\phi ))(\mu +\beta \phi ))))\\ \;/2k_{a}\eta_{f}\mu \sigma (\rho +\sigma )(\alpha_{a}\theta +(\beta +\mu )(\rho +\sigma )) \end{array};$$

$$\begin{array}{l} F_{th}{}^{DB}=(1/(2k_{a}\eta_{a}\eta_{f}\rho \sigma {\left(\rho +\sigma \right)}^{2}))((c^{2}\eta_{f}(k_{a}(\alpha_{a}\theta +(\beta +\mu )(\rho +\sigma ))((\beta +\mu )\sigma (\rho +\sigma )+\alpha_{a}\theta (2\rho \\ \;+\sigma ))+\eta_{a}(\delta_{a}\theta +\gamma (\rho +\sigma ))(\gamma \sigma (\rho +\sigma )+\delta_{a}\theta (2\rho +\sigma )))+2c(k_{a}(p_{a}\eta_{f}(\eta_{a}\sigma (\rho +\sigma )2)+\eta_{a}\\ \;\sigma (\rho +\sigma )(p_{f}\mu (\alpha_{f}\theta +(\beta +\mu )(\rho +\sigma )))))+p_{a}(p_{a}\delta_{a}\eta_{a}\eta_{f}\theta \lambda (2\gamma {\left(\rho +\sigma \right)}^{2}+\delta_{a}\theta (2-\lambda )(2\rho \\ \;+\sigma ))+k_{a}p_{a}\eta_{f}(\alpha_{a}{}^{2}\theta^{2}(2-\lambda )\lambda (2\rho +\sigma )+2\alpha_{a}\theta {\left(\rho +\sigma \right)}^{2}(\beta +\mu -\beta (1-\lambda )\phi )+\sigma {\left(\rho +\sigma \right)}^{2}\\ \;({\left(\beta +\mu \right)}^{2}))))-(2c(p_{a}\eta_{a}\eta_{f}(\delta_{a}\theta +\gamma (\rho +\sigma ))(\gamma \sigma (\rho +\sigma )+\delta_{a}\theta (2\rho +\sigma ))+k_{a}(p_{a}\eta_{f}(2\alpha_{a}\theta \\ \;(\beta +\mu ){\left(\rho +\sigma \right)}^{2}+{\left(\beta +\mu \right)}^{2}\sigma {\left(\rho +\sigma \right)}^{2}+\alpha_{a}{}^{2}\theta^{2}(2\rho +\sigma ))+\eta_{a}\sigma (\rho +\sigma )((1-a)\eta_{f}(\rho +\sigma )\\ \;)))+p_{a}k_{a}(2p_{f}\eta_{a}(\rho +\sigma )(\alpha_{f}{}^{2}\theta^{2}\lambda^{2}+\alpha_{f}\theta (\mu \sigma +\beta \lambda (\rho +2\sigma )(1-\phi ))+\sigma (\rho +\sigma )(\beta \mu +\mu^{2}\\ \;+\beta^{2}{\left(1-\phi \right)}^{2}))+p_{a}\eta_{f}(\sigma (\rho +\sigma )2\beta^{2}\phi^{2})))) \end{array}$$

Proposition 11 intuitively shows the relationship between the expected discounted profits for the brand owner and counterfeiter before and after the adoption of blockchain in the direct sales mode. Combined with Proposition 11 (i) and (ii), the research results can provide the following insights into the effect of blockchain on the supervision of counterfeit products in the supply chain in the direct sales mode: (1) If the unit operating cost *c* and fixed cost *F* of blockchain are both small, using blockchain can not only combat the counterfeiter’s profit but also improve the brand owner’s profit; (2) if *c* is small and *F* is large, the adoption of blockchain can reduce the counterfeiter’s profit but also hurt the profit of the brand owner; (3) if *c* is larger and *F* is smaller, the adoption of blockchain will not combat the counterfeiter’s profit but will improve the profit of the brand owner; and (4) if both *c* and *F* are large, the adoption of blockchain will not combat the counterfeiter’s profit and will hurt the profit of the brand owner.

Therefore, in the direct sales mode, the best condition under which the brand owner can use blockchain to achieve the supervision of counterfeit products in the supply chain is condition (1); condition (3) is suboptimal, and conditions (2) and (4) are relatively poor. These conditions can remind brand owners to weigh the relevant costs of blockchain to inhibit counterfeit products in the supply chain and improve their own profits under certain conditions.

#### Proposition 12

In the wholesale sales mode, when blockchain is established by the brand owner, the effect of blockchain on the supervision of counterfeit products in the supply chain is as follows:

- $\left\{ \begin{array}{l} if\;c\leq c_{th1}{}^{WB},\;then\;V_{f}{}^{WB}\leq V_{f}{}^{WN}\\ if\;c>c_{th1}{}^{WB},\;then\;V_{f}{}^{WB}>V_{f}{}^{WN} \end{array}\right.$;
- $\left\{ \begin{array}{l} if\;F\leq F_{th}{}^{WB},\;then\;V_{b}{}^{WB}\geq V_{b}{}^{WN}\\ if\;F>F_{th}{}^{WB},\;then\;V_{b}{}^{WB}<V_{b}{}^{WN} \end{array}\right.$;
- $\left\{ \begin{array}{l} if\;c\leq c_{th2}{}^{WB},\;then\;V_{r}{}^{WB}\geq V_{r}{}^{WN}\\ if\;c>c_{th2}{}^{WB},\;then\;V_{r}{}^{WB}<V_{r}{}^{WN} \end{array}\right.$;

where

$$\begin{array}{l} c_{th}{{}_{1}}^{WB}=w_{a}+((\sigma \eta_{f}(q_{f}{{}^{WB*}}^{{}^{2}}-q_{f}{{}^{WN*}}^{{}^{2}})+s_{a}{}^{WN*}\delta_{a}\theta +q_{a}{}^{WN*}\alpha_{a}\theta (1-\lambda )+2p_{f}(q_{f}{}^{WN*}\alpha_{f}\theta \lambda +q_{f}{}^{WN*}\beta \sigma \\ \;+q_{a}{}^{WN*}\beta \sigma \phi ))-(2p_{f}(q_{f}{{}^{WB}}^{*}\alpha_{f}\theta +s_{a}{{}^{WN}}^{*}\delta_{a}\theta \lambda +q_{f}{{}^{WB}}^{*}(\beta +\mu )\sigma +q_{f}{{}^{WN}}^{*}\beta \sigma \phi )))\eta_{a}(\rho +\sigma \\ \;)/(p_{f}\mu \sigma (\alpha_{a}\theta +(\beta +\mu )(\rho +\sigma ))) \end{array};$$

$$\begin{array}{l} c_{th}{{}_{2}}^{WB}=\eta_{a}(\rho +\sigma )((2q_{f}{{}^{WN}}^{*}w_{a}\alpha_{f}\theta \lambda +2q_{f}{{}^{WN}}^{*}w_{a}\beta \sigma +2q_{f}{{}^{WB}}^{*}wa\mu \sigma +2q_{a}{{}^{WN}}^{*}w_{a}(\alpha_{a}(\theta -\theta \lambda )+\beta \sigma \phi )\\ \;+2p_{a}(\delta_{a}\theta (s_{a}{{}^{WB}}^{*}-s_{a}{{}^{WN}}^{*}(1-\lambda ))+\gamma \sigma (s_{a}{{}^{WB}}^{*}-s_{a}{{}^{WN}}^{*})+q_{f}{{}^{WN}}^{*}\beta \sigma \phi ))-(2w_{a}\delta_{a}\theta (s_{a}{}^{WB*}-s_{a}{}^{WN*}\\ \;(1-\lambda ))+k_{a}\sigma (s_{a}{{}^{WB*}}^{{}^{2}}-s_{a}{{}^{WN*}}^{{}^{2}})+2w_{a}\gamma \sigma (s_{a}{}^{WB*}-s_{a}{}^{WN*})+2q_{f}{}^{WN*}w_{a}\beta \sigma \phi +2p_{a}(q_{a}{}^{WN*}\alpha_{a}\theta (\\ \;1-\lambda ))+q_{f}{}^{WN*}\alpha_{f}\theta \lambda +q_{f}{}^{WN*}\beta \sigma +q_{f}{}^{WB*}\mu \sigma +q_{a}{}^{WN*}\beta \sigma \phi ))/(2(p_{a}-w_{a})(\alpha_{a}\theta +(\beta +\mu )\\ \;\sigma )(\alpha_{a}\theta +(\beta +\mu )(\rho +\sigma )))+w_{a} \end{array};$$

$$\begin{array}{l} F_{th}{}^{WB}=((2q_{a}{{}^{WB}}^{*}w_{a}\alpha_{a}\theta +2s_{a}{{}^{WB}}^{*}w_{a}\delta_{a}\theta +2s_{a}{{}^{WN}}^{*}w_{a}\delta_{a}\theta \lambda +2q_{a}{{}^{WB}}^{*}w_{a}\beta \sigma +2s_{a}{{}^{WB}}^{*}w_{a}\gamma \sigma +q_{a}{{}^{WN}}^{*}{}^{{}^{2}}\eta_{a}\sigma +\\ \;2q_{a}{{}^{WB}}^{*}w_{a}\mu \sigma +2c(p_{a}+q_{f}{{}^{WB}}^{*}\mu )\sigma +2q_{f}{{}^{WN}}^{*}w_{a}\beta \sigma \phi )-(2s_{a}{{}^{WN}}^{*}w_{a}\delta_{a}\theta +2c(q_{a}{{}^{WB}}^{*}\alpha_{a}+s_{a}{{}^{WB}}^{*}\delta_{a}\\ \;)\theta +2q_{f}{{}^{WN}}^{*}w_{a}\alpha_{f}\theta \lambda +2q_{f}{{}^{WN}}^{*}w_{a}\beta \sigma +2s_{a}{{}^{WN}}^{*}w_{a}\gamma \sigma +qa^{WB}{{}^{*}}^{{}^{2}}\eta_{a}\sigma +2q_{f}{{}^{WB}}^{*}w_{a}\mu \sigma +2c(1-a\\ \;+s_{a}{{}^{WB}}^{*}\gamma +q_{a}{{}^{WB}}^{*}(\beta +\mu ))\sigma +2q_{a}{{}^{WN}}^{*}w_{a}(\alpha_{a}\theta (1-\lambda )+\beta \sigma \phi )))/2\rho \sigma \end{array}.$$

According to Proposition 12, when a brand owner establishes blockchain in the wholesale sales mode, the effect of blockchain on the supervision of counterfeit products in the supply chain can be expressed as follows: (1) If the unit operating cost of the blockchain is *c*≤min{*c*_*th*1_^*WB*^, *c*_*th*2_^*WB*^} and the fixed cost is *F*≤*F*_*th*_^*WB*^, we find that the establishment of blockchain by the brand owner can not only combat the counterfeiter’s profit but also improve the profits for both the brand owner and the retailer; (2) if *c*≤min{*c*_*th*1_^*WB*^, *c*_*th*2_^*WB*^} and the fixed cost *F*>*F*_*th*_^*WB*^, we find that the brand owner can reduce crack down on the counterfeiter’s profit and improve the retailer’s profit by using blockchain, but the brand owner will damage its own profit; (3) if *c*_*th*1_^*WB*^< *c*≤*c*_*th*2_^*WB*^ and the fixed cost *F*≤*F*_*th*_^*WB*^, we find that the adoption of blockchain cannot combat the profit of the counterfeiter, but improve the profit of the brand owner and retailer; (4) if *c*_*th*1_^*WB*^<*c*≤*c*_*th*2_^*WB*^ and the fixed cost *F*>*F*_*th*_^*WB*^, we find that the adoption of blockchain by the brand owner cannot combat the counterfeiter’s profit, and the brand owner will damage its own profit but improve the profit of the retailer; (5) if *c*_*th*2_^*WB*^<*c*≤*c*_*th*1_^*WB*^ and the fixed cost *F*≤*F*_*th*_^*WB*^, we find that the brand owner’s adoption of blockchain can combat the counterfeiter’s profit and improve its own profit but damage the retailer’s profit; (6) if *c*_*th*2_^*WB*^<*c*≤*c*_*th*1_^*WB*^ and the fixed cost *F*>*F*_*th*_^*WB*^, we find that blockchain can combat the counterfeiter’s profit but damage the profit for the brand owner and retailer; (7) if *c*>max{*c*_*th*1_^*WB*^, *c*_*th*2_^*WB*^} and the fixed cost is *F*≤*F*_*th*_^*WB*^, we find that the adoption of blockchain by the brand owner cannot combat the counterfeiter’s profit, and it damages the profit for the retailer but improve its own profit; and (8) if *c*>max{*c*_*th*1_^*WB*^, *c*_*th*2_^*WB*^} and the fixed cost *F*>*F*_*th*_^*WB*^, the adoption of blockchain by the brand owner cannot combat the counterfeiter’s profit, and it damages the profit for both the brand owner and retailer. Therefore, the best condition under which a brand owner can implement blockchain in the wholesale sales mode is condition (1). At this time, adopting blockchain under this condition not only combat the counterfeiter’s profits but also ensures that the profits for retailers are not damaged; as a result, the retailer is encouraged to participate in the implementation of blockchain, and at the same time, the brand owner’s profit is also improved.

#### Proposition 13

In the wholesale sales mode, when blockchain is established by the retailer, the effect of blockchain on the supervision of counterfeit products in the supply chain is as follows:

- $\left\{ \begin{array}{l} if\;p_{a}\leq p_{a}{{}_{th}}^{WR},\;then\;V_{f}{}^{WR}\geq V_{f}{}^{WN}\\ if\;p_{a}>p_{a}{{}_{th}}^{WR},\;then\;V_{f}{}^{WR}<V_{f}{}^{WN} \end{array}\right.$;
- $\left\{ \begin{array}{l} if\;c\leq c_{th}{}^{WR},\;then\;V_{b}{}^{WR}\geq V_{b}{}^{WN}\\ if\;c>c_{th}{}^{WR},\;then\;V_{b}{}^{WR}<V_{b}{}^{WN} \end{array}\right.$;
- $\left\{ \begin{array}{l} if\;F\leq F_{th}{}^{WR},\;then\;V_{r}{}^{WR}\geq V_{r}{}^{WN}\\ if\;F>F_{th}{}^{WR},\;then\;V_{r}{}^{WR}<V_{r}{}^{WN} \end{array}\right.$;

where

$$\begin{array}{l} p_{a}{{}_{th}}^{WR}=((2p_{f}(q_{f}{}^{WR*}\alpha_{f}\theta +q_{f}{}^{WR*}(\beta +\mu )\sigma +(w_{a}\delta_{a}\theta (1-\lambda )(\delta_{a}\theta (1-\lambda )+\gamma (\rho +\sigma )))/(k_{a}(\rho +\sigma \\ \;))+q_{f}{}^{WN*}\beta \sigma \phi ))-(\eta_{f}\sigma (q_{f}{{}^{WR*}}^{{}^{2}}-q_{f}{{}^{WN*}}^{{}^{2}})+2p_{f}(q_{a}{}^{WN*}\alpha_{a}\theta (1+\lambda )+q_{f}{}^{WN*}\alpha_{f}\theta \lambda +q_{f}{}^{WN*}\\ \;\beta \sigma +q_{a}{}^{WR*}\mu \sigma +q_{a}{}^{WN*}\beta \sigma \phi )))/((2p_{f}\delta_{a}\theta (1-\lambda )(\delta_{a}\theta (1-\lambda )+\gamma (\rho +\sigma )))/(k_{a}(\rho +\sigma ))) \end{array};$$

$$\begin{array}{l} c_{th}{}^{WR}=p_{a}-w_{a}+((2q^{WR*}w_{a}(\alpha_{a}\theta +(\beta +\mu )\sigma ))-(\eta_{a}\sigma (q_{a}{{}^{WR*}}^{{}^{2}}-q_{a}{{}^{WN*}}^{{}^{2}})+2w_{a}(q_{f}{}^{WN*}\alpha_{f}\theta \lambda +s_{a}{}^{WN*}\\ \;\delta_{a}(\theta -\theta \lambda )+sa^{WN*}\gamma \sigma +q_{f}{}^{WR*}\mu \sigma +q_{f}{}^{WN*}\beta \sigma (1-\phi ))+2q_{a}{}^{WN*}w_{a}(\alpha_{a}\theta (1-\lambda )+\beta \sigma \phi )))k_{a}\\ \;(\rho +\sigma )/(2w_{a}(\delta_{a}\theta +\gamma \sigma )(\delta_{a}\theta +\gamma (\rho +\sigma ))) \end{array};$$

$$\begin{array}{l} F_{th}{}^{WR}=((2s_{a}{}^{WN*}w_{a}\delta_{a}\theta (1-\lambda )+2q_{f}{}^{WN*}w_{a}\alpha_{f}\theta \lambda +k_{a}\sigma s_{a}{{}^{WN*}}^{{}^{2}}+2q_{f}{}^{WN*}w_{a}\beta \sigma +2s_{a}{}^{WN*}w_{a}\gamma \sigma +2q_{f}{}^{WR*}\\ \;w_{a}\mu \sigma +2c(q_{f}{}^{WR*}\mu \sigma )+2(q_{a}{}^{WN*}-q_{f}{}^{WN*})w_{a}\beta \sigma \phi +2p_{a}(q_{a}{}^{WR*}\alpha_{a}\theta +s_{a}{}^{WN*}\delta_{a}\theta \lambda +c\sigma +q_{a}{}^{WR*}\\ \;(\beta +\mu )\sigma +q_{f}{}^{WN*}\beta \sigma \phi ))-(2w_{a}\alpha_{a}\theta (q_{a}{}^{WR*}-(1-\lambda )q_{a}{}^{WN*})+2q_{a}{}^{WR*}w_{a}\sigma (\beta +\mu )+2c(q_{a}{}^{WR*}\\ \;\alpha_{a}\theta +q_{a}{}^{WR*}(\beta +\mu )\sigma +(1-a)\sigma )+2p_{a}(s_{a}{}^{WN*}(\delta_{a}\theta +\gamma \sigma )+qa^{WN*}(\theta \alpha_{a}(1-\lambda )+\beta \sigma \phi )+\\ \;q_{f}{}^{WN*}(\alpha_{f}\theta \lambda +\beta \sigma )+q_{f}{}^{WR*}\mu \sigma ))/2\rho \sigma \end{array}.$$

In Proposition 13, by comparing the *WR* case, in which retailers establish blockchain, with the *WN* case, in which blockchain is not implemented in the wholesale sales mode, we find that the relationship between the expected discounted profits for the counterfeiter, brand owner and retailer in terms of blockchain adoption depends on the sales price *p*_*a*_ of genuine products, the unit operating cost *c* of blockchain and the fixed cost *F*, which is different from Proposition 12. According to Proposition 13 (i)~(iii), if and only if *p*_*a*_>*p*_*ath*_^*WR*^, *c*≤*c*_*th*_^*WR*^ and *F*≤*F*_*th*_^*WR*^, the retailer’s establishment of blockchain in wholesale sales mode can combat the counterfeiter’s profit and improve the profits for itself and the brand owner, which implies that if the sales price positioning of genuine products is high but the related costs of blockchain are small, the retailer is more motivated to establish blockchain, as it not only reduces the counterfeiter’s profit but also benefits genuine enterprises.

## 7 Numerical analysis

The equilibrium results under different cases have been discussed in detail above. However, due to the complexity of the expected discounted profits of supply chain members and the conditions for genuine enterprises to supervise the effect of counterfeit products in the supply chain, it is difficult to intuitively judge the impact of some key parameters. Therefore, this section will conduct numerical research through MATLAB software to further explore the impact of key parameters on supply chain performance in different cases, and further study the impact of key parameters on the supervisory effect of supply chain counterfeit products according to the results in Section 6.4 above, in order to get the corresponding managerial insights. Referring to Shen et al. [1] and Pun et al. [4] on blockchain and Ma et al. [8] on brand goodwill, and accounting for the specific background of this paper, the basic parameters are set as follows: *a* = 0.4; *p*_*f*_ = 0.28; *p*_*a*_ = 0.3; *w* = 0.2; *ρ* = 0.1; *F* = 0.1; *G*_*0*_ = 0.1; *α*_*f*_ = 0.7; *α*_*a*_ = 0.8; *δ*_*f*_ = 0.4; *δ*_*a*_ = 0.5; *σ* = 0.05; *λ* = 0.3; *θ* = 0.1; *k*_*a*_ = 0.01; *η*_*f*_ = 0.02; *η*_*a*_ = 0.01; *β* = 0.6; *γ* = 0.4; *ϕ* = 0.6; *u* = 0.25; and *c* = 0.03.

### 7.1 Impacts of key parameters on supply chain performance in different cases

**Fig 2:**
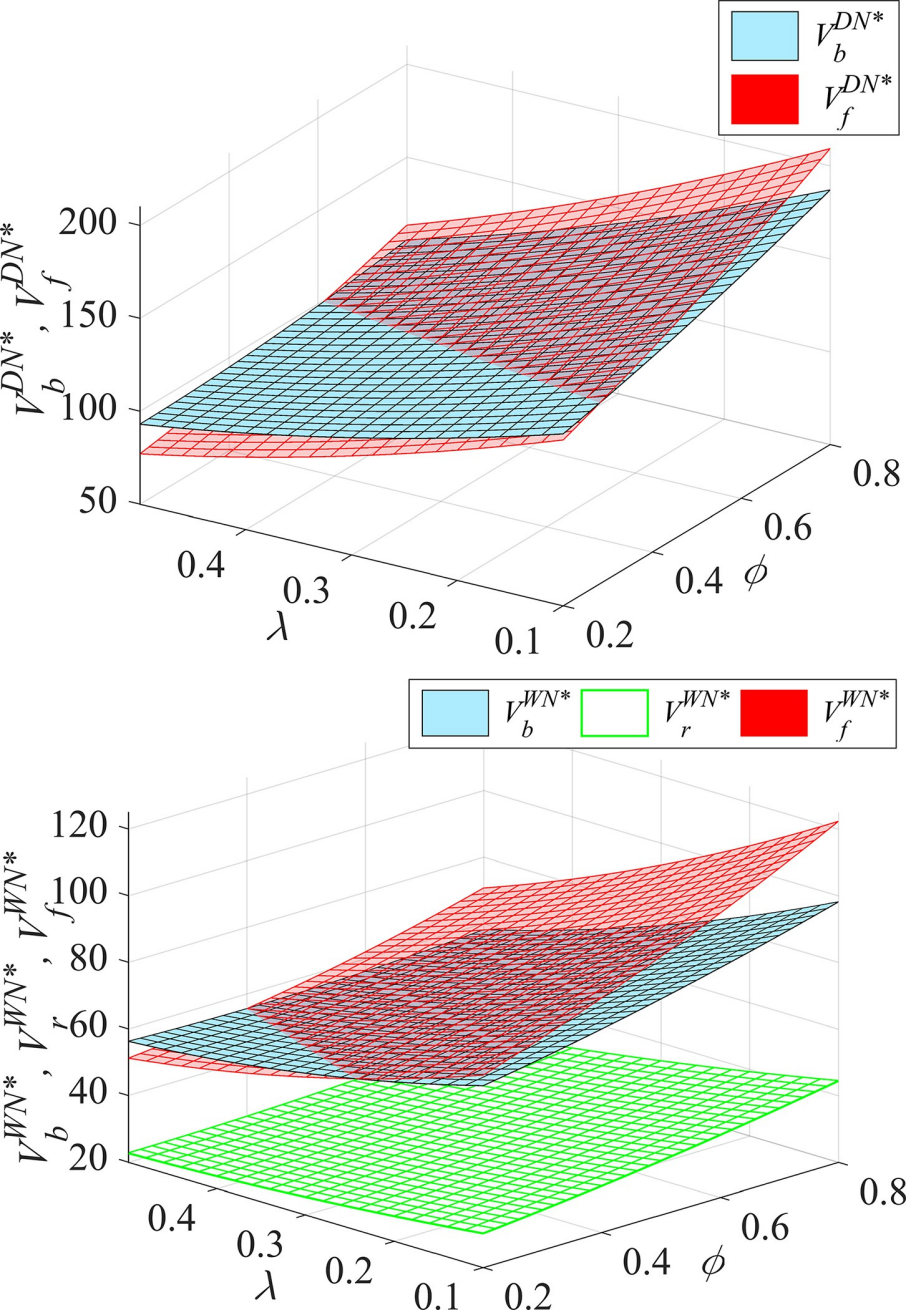
Effects of *λ* and *ϕ* on supply chain profits without blockchain.

**Fig 3:**
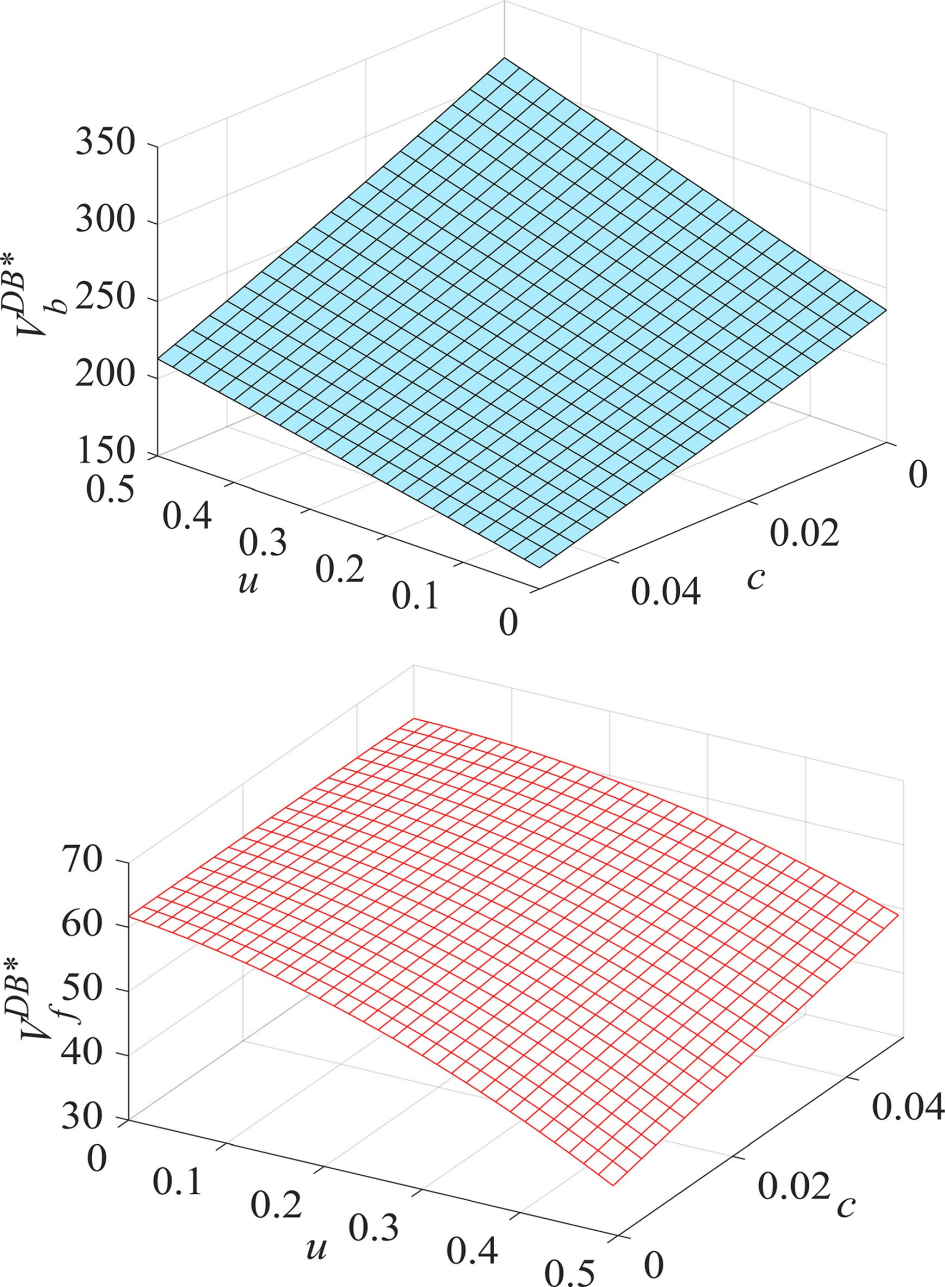
Effects of *u* and *c* on supply chain profits in the *DB* case.

**Fig 4:**
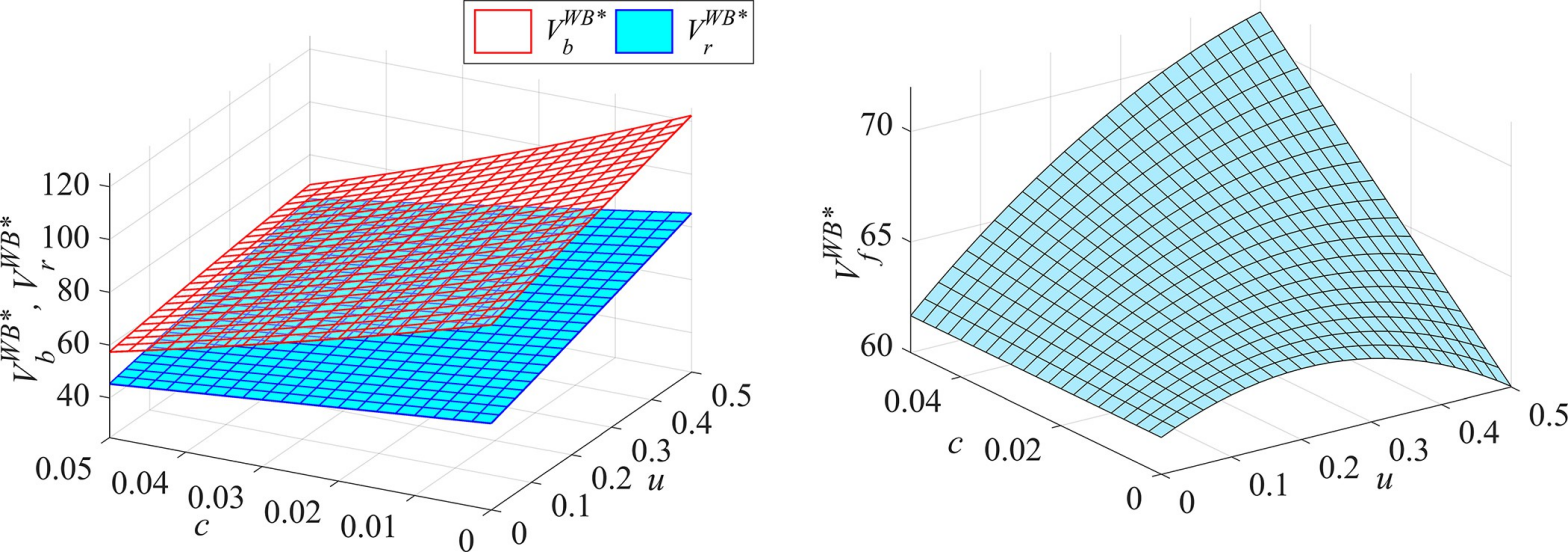
Effects of *u* and *c* on supply chain profits in the *WB* case.

**Fig 5:**
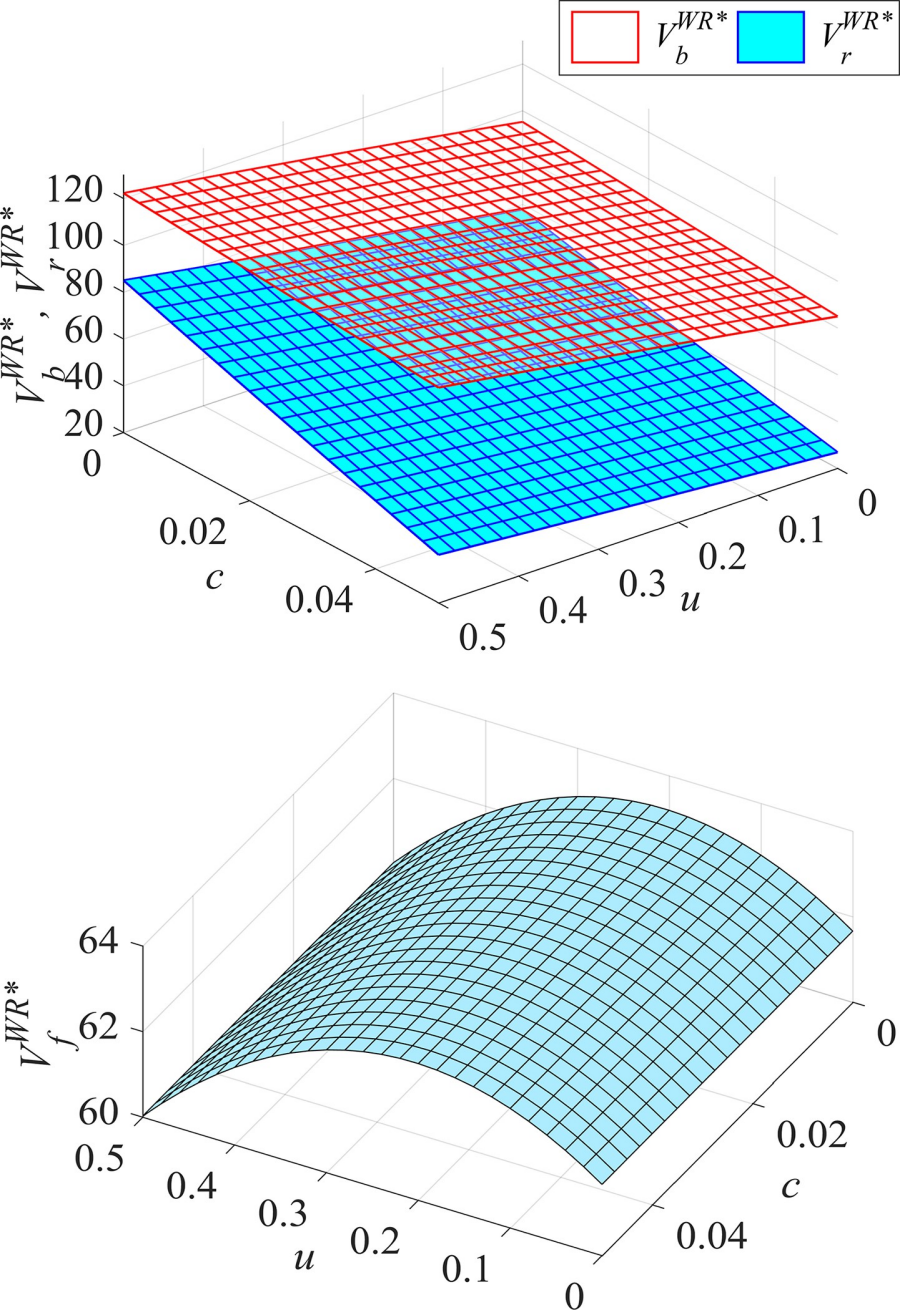
Effects of *u* and *c* on supply chain profits in the *WR* case.

The simulation results in Figs 2–5 show the impact of four key parameters on the supply chain performance under different scenarios. These include the influence degree *λ* of counterfeit products on brand goodwill, the probability *ϕ* that consumers consider the products to be genuine when there is no blockchain, and the reference utility coefficient *u* of consumers’ perception of the quality of counterfeit products and genuine products under blockchain technology and the unit operating cost *c* of the blockchain.

As seen from Fig 2, the profits for the counterfeiter and genuine enterprises in the two sales modes without blockchain are positively related to parameter *ϕ*, while both are negatively related to parameter *λ*. This relationship shows that when there is no blockchain, regardless of whether the brand owner chooses the direct sales mode or wholesale sales mode, due to the asymmetry of quality information, when the probability of consumers believing that the product is genuine increases, it is beneficial to the genuine enterprises, but the counterfeiter also makes more profit. However, as the impact of counterfeit products on brand goodwill increases, consumers’ image and reputation of the brand products deteriorate, and the profits of the counterfeiter and genuine enterprises will be negatively affected. In addition, it can also be found from Fig 2 that the counterfeiter’s profit is related to parameter *ϕ* and is greater than that of genuine enterprises, while the sensitivity of genuine enterprises’ profits with respect to parameter *λ* is greater than that of the counterfeiter. This phenomenon implies that an increase in parameter *ϕ* is more profitable for the counterfeiter, while an increase in parameter *λ* is more detrimental for genuine enterprises. Information asymmetry usually has a greater impact on the profitability of genuine enterprises relative to counterfeiters.

Fig 3 intuitively shows that when a brand owner adopts blockchain in the direct sales mode, the brand owner’s profit is positively correlated with parameter *u* and negatively correlated with parameter *c*. In contrast, the counterfeiter’s profit is negatively correlated with parameter *u* but positively correlated with parameter *c*. This phenomenon reveals that the quality reference effect of consumers on genuine and counterfeit products is beneficial to brand owners but unfavorable to counterfeiters because brand owners usually have a higher quality level than counterfeiters, which can improve consumers’ demand for genuine products and combat the demand for counterfeit products. In addition, brand owners will reduce the quality of genuine products to reduce the cost expenditure due to the increase in the blockchain unit operation cost, which is intuitively unfavorable to brand owners. However, this decision is beneficial to counterfeiters because the reduction in the quality of genuine products indirectly affects the sales of counterfeit products due to the consumer quality reference effect so that counterfeiters can obtain additional profits. Especially when parameter *u* is large, with an increase in parameter *c*, the counterfeiter will obtain extra profits.

Fig 4 presents the impact of parameters *u* and *c* on the profits of counterfeiter and genuine enterprises when blockchain is established by the brand owner in the wholesale sales mode. The difference from Fig 3 is that if parameter *c* is small, the counterfeiter’s profit first increases and then decreases with the increase in parameter *u*, while if parameter *c* is large, the counterfeiter’s profit increases with the increase in parameter *u*. This result indicates that the impact of parameter *u* on the counterfeiter’s profit depends on parameter *c* and is related to the brand owner’s sales modes. The reason is that in the wholesale sales mode, the brand owner’s decision about the quality of its genuine products is different from that in the direct sales mode, which affects the sensitivity of genuine products’ quality with parameters *u* and *c*, thus indirectly affecting the variation in the counterfeiter’s profit with parameter *u*. When the blockchain unit operation cost is large, the brand owner should not establish blockchain. At this time, the cost of blockchain seriously deteriorates the quality of genuine products and increases the counterfeiter’s profit under the quality reference effect of consumers. It can be seen from Fig 5 that when blockchain is established by a retailer using the wholesale sales mode, the counterfeiter’s profit is first positively correlated and then negatively correlated with parameter *u* but not with parameter *c*. This relationship is different from that when the brand owner establishes blockchain. The reason is that the retailer bears the unit operation cost of the blockchain at this time. The decision by the brand owner regarding the quality of its genuine products is not affected by parameter *c*, and the counterfeiter’s decision regarding the quality of its counterfeit products is not affected. In addition, according to the comprehensive Figs 2–5, regardless of whether blockchain is adopted, the brand owner usually has a better profit effect under the direct sales mode, which explains why, in practice, some large brand owners with independent production and marketing ability prefer to establish direct franchised stores for sales. Moreover, the adoption of blockchain does not affect this advantage.

### 7.2 Impacts of key parameters on the supervisory effect of blockchain in different cases

According to Propositions 11 to 13, this section further explores the impacts of key parameters on the supervisory effect of blockchain technology-based counterfeit products in the direct and wholesale sales modes to reveal the sensitivity of the conditions for the supervisory effect of genuine enterprises to key parameters, thereby providing new insights for managers.

**Fig 6:**
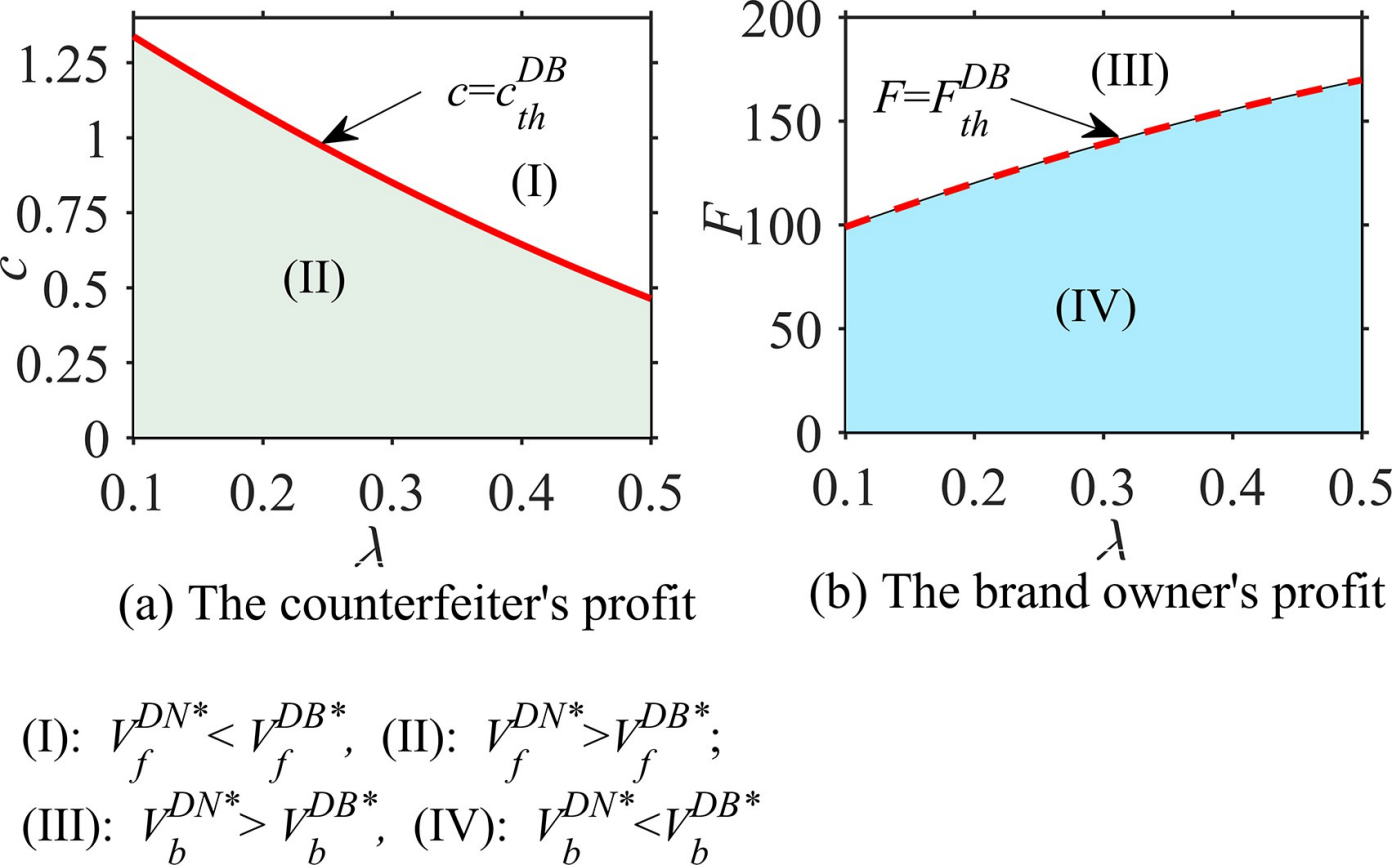
Impact of *λ* on the supervisory effect of blockchain in the direct sales mode.

**Fig 7:**
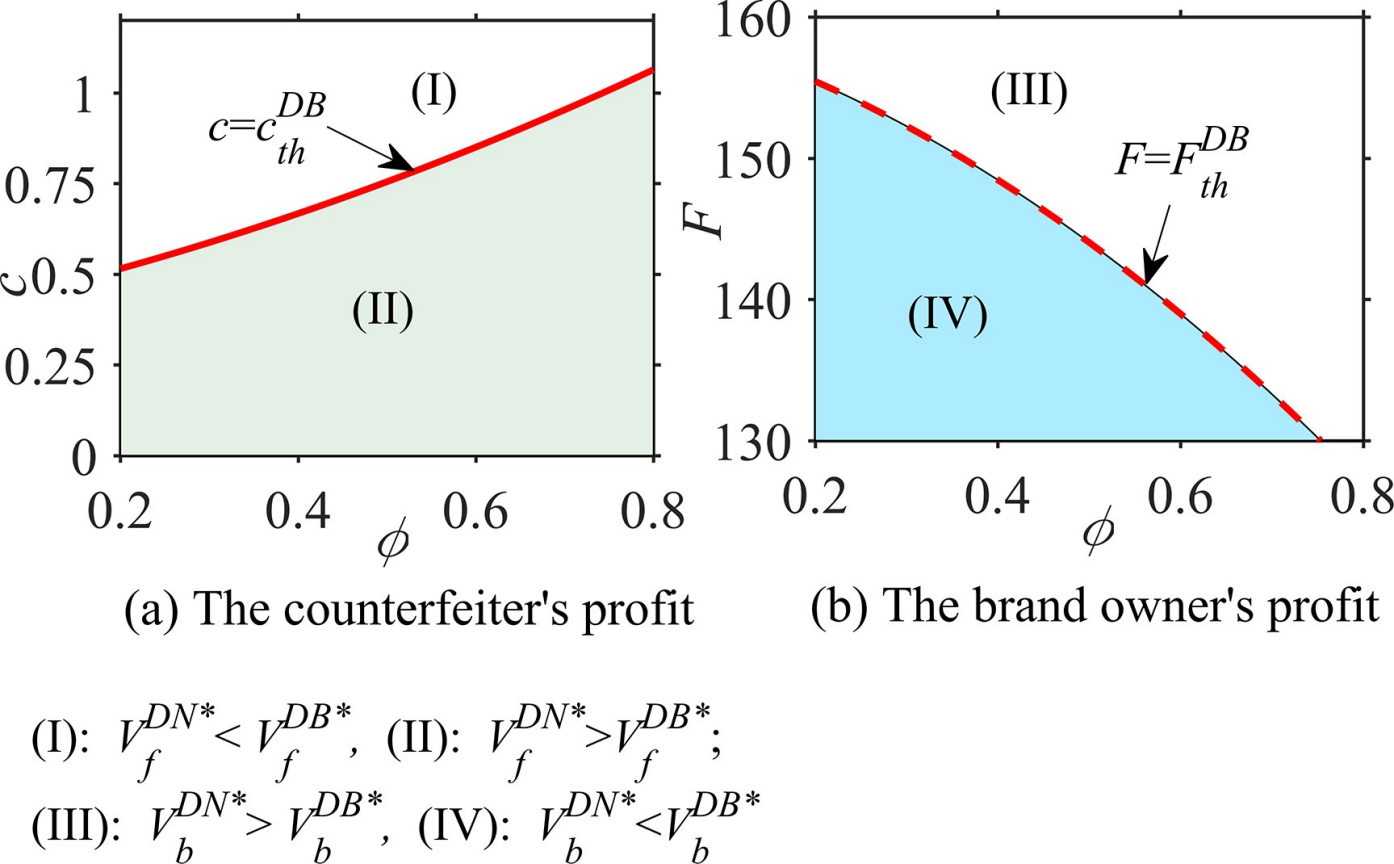
Impact of *ϕ* on the supervisory effect of blockchain in the direct sales mode.

**Fig 8:**
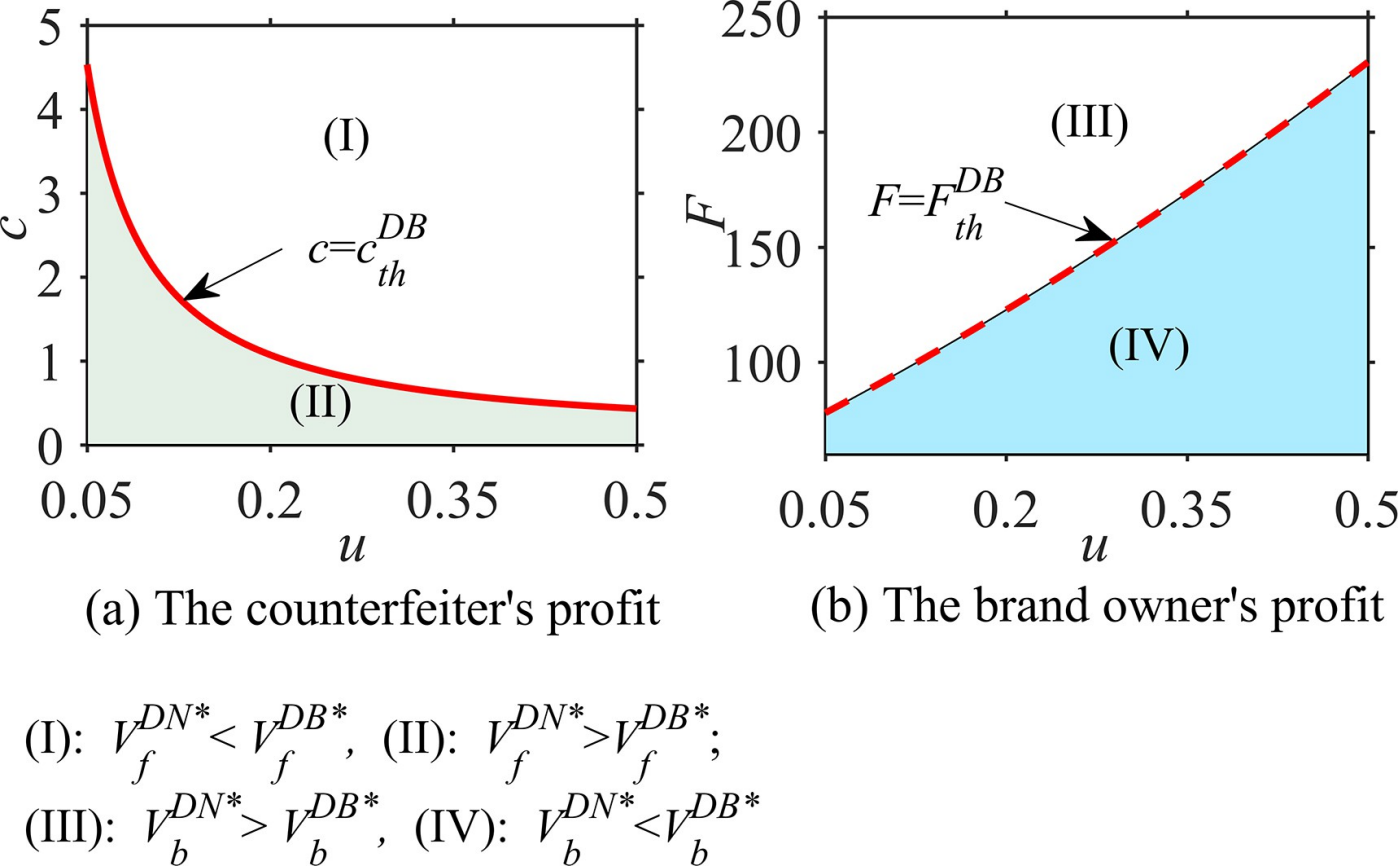
Impact of *u* on the supervisory effect of blockchain in the direct sales mode.

According to the analysis of Proposition 11, in the direct sales mode, if and only if the unit operating cost *c* and fixed cost *F* of the blockchain are small, that is, *c*≤*c*_*th*_^*DB*^ and *F*≤*F*_*th*_^*DB*^, are the best conditions for the brand owner to use blockchain to supervise counterfeit products in the supply chain. Therefore, the greater the threshold *c*_*th*_^*DB*^ of the blockchain unit operating cost and the threshold *F*_*th*_^*DB*^ of the fixed cost of the blockchain, the more conducive it is for brands to implement blockchain. Figs 6–8 show that the impacts of the key parameters *λ*, *ϕ* and *u* on the supervision effect of counterfeit products in the supply chain under the direct sales mode. In Fig 6, as parameter *λ* increases, *c*_*th*_^*DB*^ decreases and *F*_*th*_^*DB*^ increases, which indicates that before the implementation of blockchain, the greater the impact of counterfeit products on brand goodwill, the more conducive it is for brand owners to implement blockchain to improve their own profits, but the less conducive it is to combat the profits of counterfeiters. This result also implies that from the perspective of selfishness alone, the increase in parameter *λ* provides the brand owner with a greater incentive to implement blockchain.

Fig 7 shows that *c*_*th*_^*DB*^ is positively correlated with parameter *ϕ*, while *F*_*th*_^*DB*^ is negatively correlated with parameter *ϕ*, which is contrary to the result in Fig 5. Before the implementation of blockchain, if the probability *ϕ* of consumers perceiving the product as genuine is higher, the more it will help the brand owner to implement blockchain to combat counterfeiters’ profitability, but the less it will help to improve its own profitability. This relationship implies that from a self-interest perspective, an increase in parameter *ϕ* will reduce the incentive for brand owners to implement blockchain. Fig 8 shows that as parameter *u* increases, *c*_*th*_^*DB*^ decreases monotonically and levels off, while *F*_*th*_^*DB*^ increases monotonically. This phenomenon is similar to the result in Fig 6 and means that the stronger the quality reference effect of consumers on genuine and counterfeit products after the implementation of blockchain, the more conducive it is for brand owners to implement blockchain to improve their profits, but the less conducive it is to the profits of counterfeiters. However, with the continuous enhancement of the consumer quality reference effect, this disadvantage will gradually weaken.

**Fig 9:**
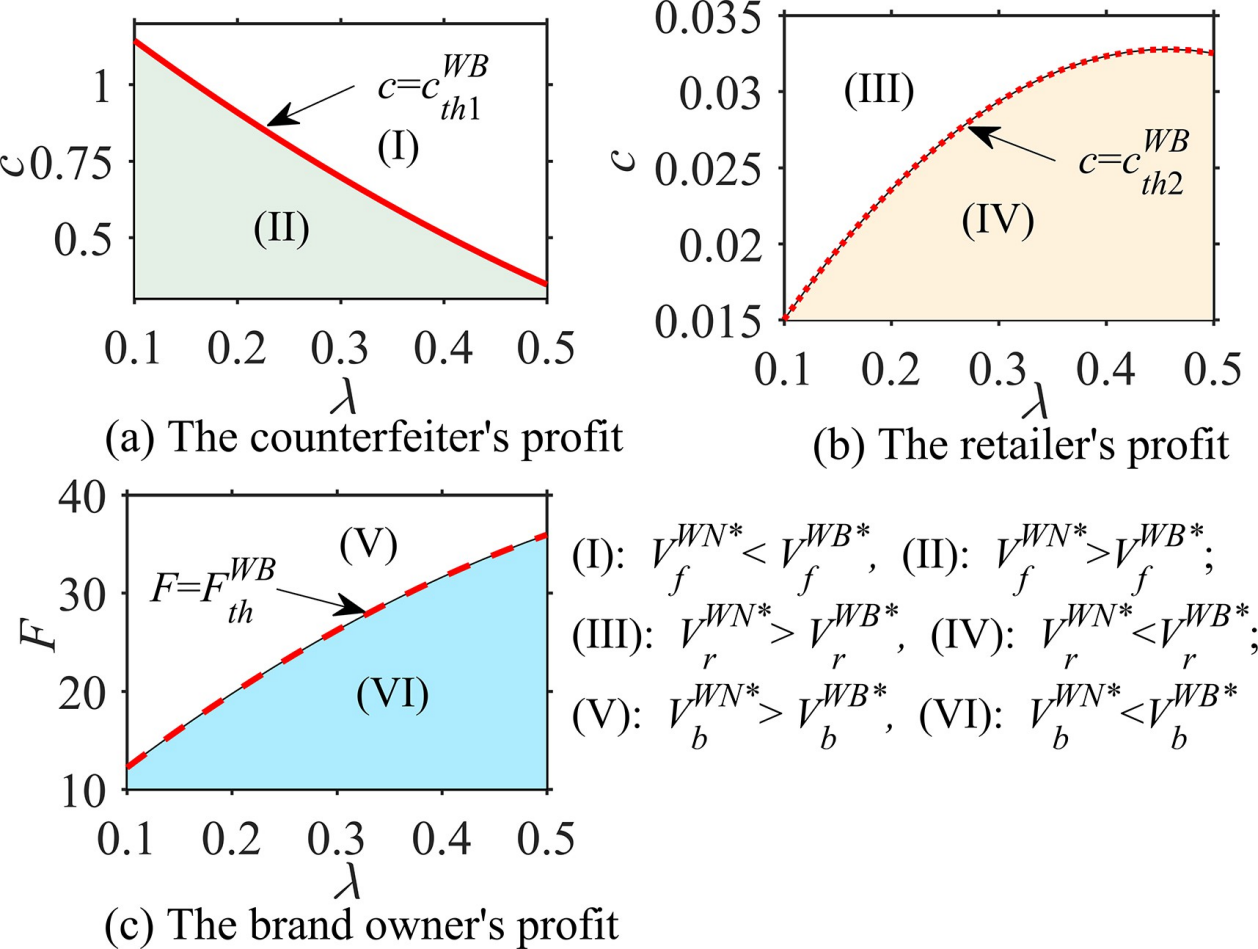
Impact of *λ* on the supervisory effect of blockchain in the *WB* case.

**Fig 10:**
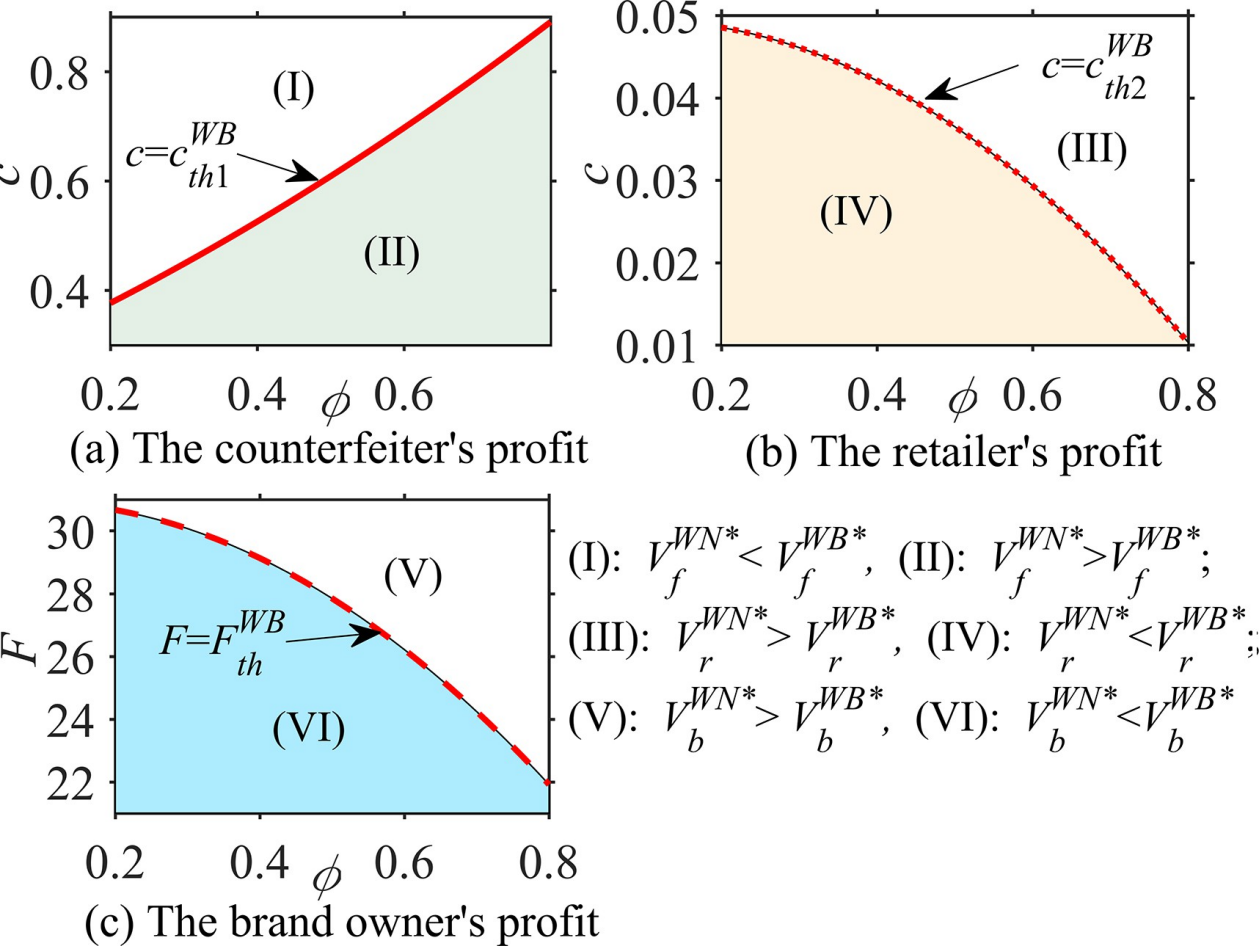
Impact of *ϕ* on the supervisory effect of blockchain in the *WB* case.

**Fig 11:**
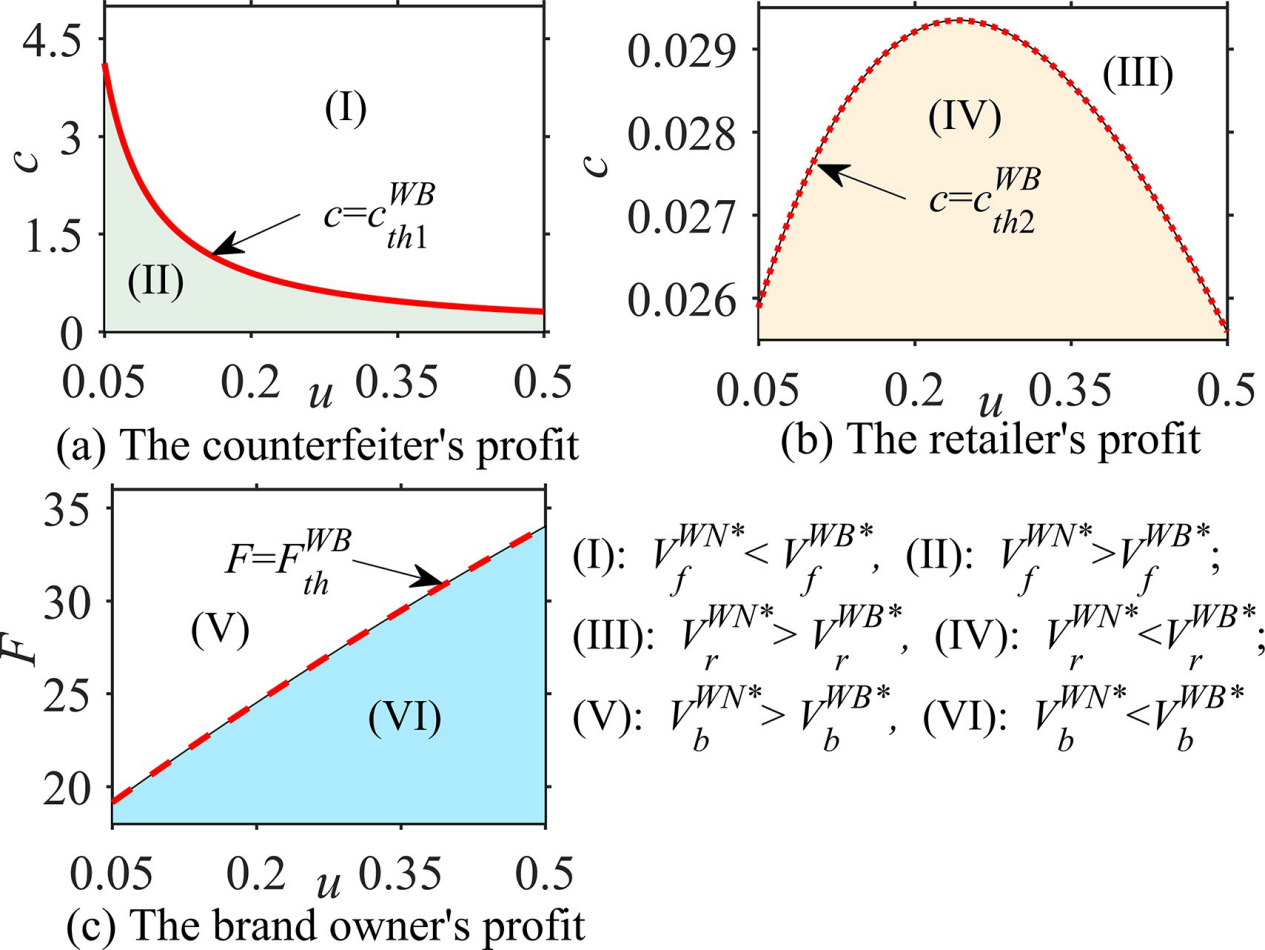
Impact of *u* on the supervisory effect of blockchain in the *WB* case.

Figs 9–11 show the impacts of key parameters *l*, *ϕ* and *u*, respectively, on the supervision effect of supply chain counterfeit products. According to the analysis of Proposition 12, if and only if the blockchain’s unit operating cost *c*≤min{*c*_*th*1_^*WB*^, *c*_*th*2_^*WB*^} and the fixed cost *F*≤*F*_*th*_^*WB*^ are there optimal conditions under which a brand owner can establish blockchain to supervise counterfeit products in the supply chain. In Fig 9, with the increase in parameter *l*, *c*_*th*1_^*WB*^ monotonically decreases; *c*_*th*2_^*WB*^ monotonically decelerates and increases, but *F*_*th*_^*WB*^ monotonically increases. This phenomenon shows that before the implementation of blockchain, the greater the impact of counterfeit products on brand goodwill, the more unfavorable it is for the brand owner to implement blockchain to crack down on the counterfeiter’s profit, but the more conducive it is to improve the profits for itself and the retailer. Therefore, from the perspective of genuine enterprise profitability alone, the increase in parameter *λ* improves the brand owner’s motivation to adopt blockchain and the retailer’s motivation to participate in blockchain.

According to the observation in Fig 10, *c*_*th*1_^*WB*^ is positively correlated with parameter *ϕ*, while *c*_*th*2_^*WB*^ and *F*_*th*_^*WB*^ are negatively correlated with parameter *ϕ*. This result suggests that the greater the probability *ϕ* of consumers perceiving a product as genuine before the implementation of blockchain, the more it will help the brand owner to implement blockchain to combat counterfeiters’ profitability, but the less it will help to improve its own and the retailer’s profitability. Therefore, the increase in parameter *ϕ* reduces not only the incentive for brand owners to adopt blockchain but also the incentive for retailers to participate in blockchain from the perspective of the profitability of the genuine enterprise alone.

Fig 11 shows that *c*_*th*1_^*WB*^, *c*_*th*2_^*WB*^ and *F*_*th*_^*WB*^ are convex functions with monotonic deceleration decreasing of parameter *u*, concave functions that first increase and then decrease, and monotonically increasing functions, respectively. This result indicates that the stronger the reference effect of consumers on the quality of genuine and counterfeit products after the implementation of blockchain, the more unfavorable it is for the brand owner to implement blockchain to reduce the counterfeiter’s profit, and the more conducive it is to improve its own profit. When parameter *u* is less than a critical value, the more conducive it is to improve the retailer’s profit; otherwise, it is more unfavorable in terms of improving the retailer’s profit. This result implies that from the perspective of the profits of genuine enterprises, when the quality reference effect of consumers is small, the increase in parameter *u* not only improves the brand owner’s motivation to adopt blockchain but also encourages retailers to participate in blockchain. However, when the quality reference effect is large, the increase in parameter *u* increases the brand owner’s motivation to adopt blockchain but reduces the retailer’s motivation to participate in blockchain.

**Fig 12:**
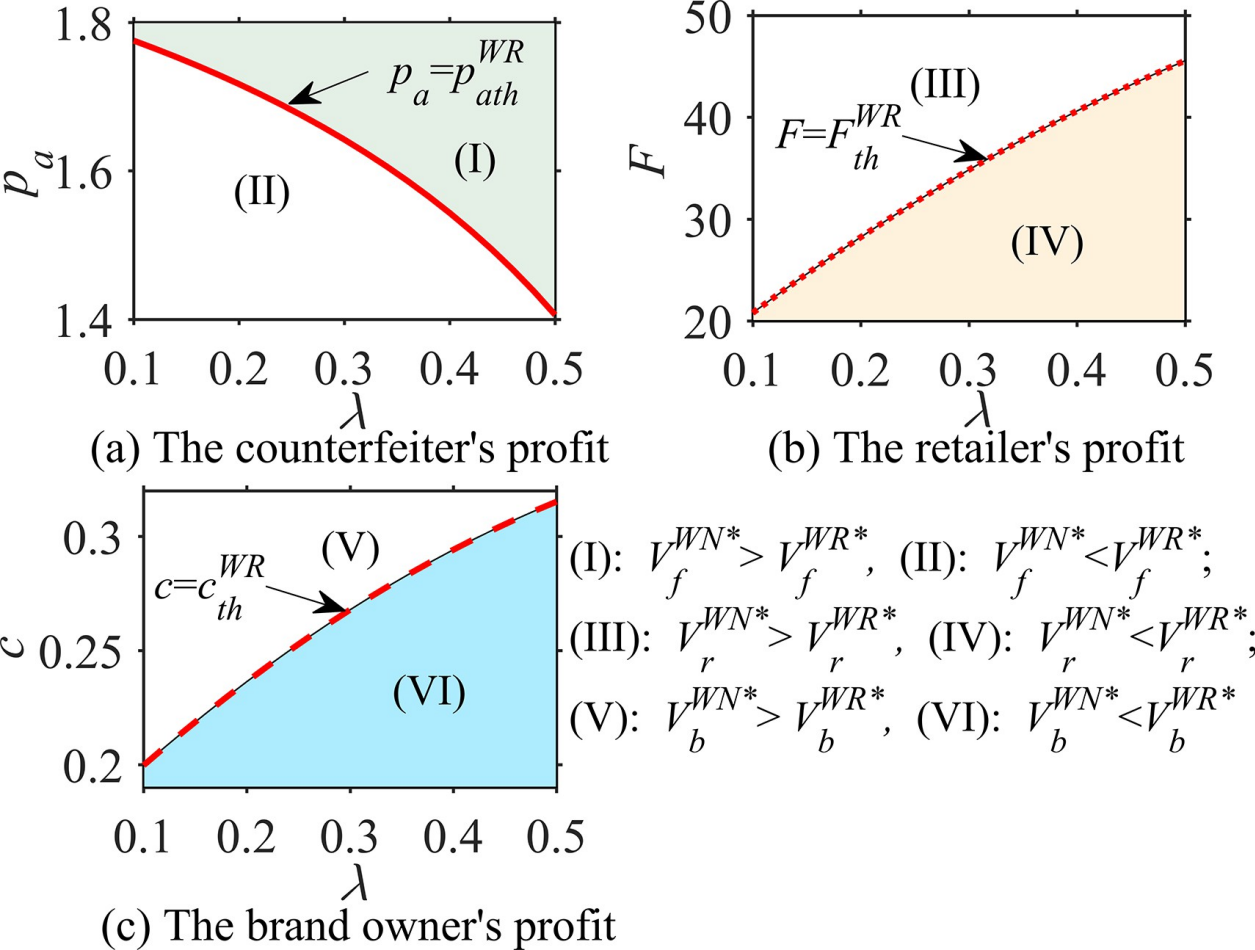
Impact of*λ* on the supervisory effect of blockchain in the *WR* case.

**Fig 13:**
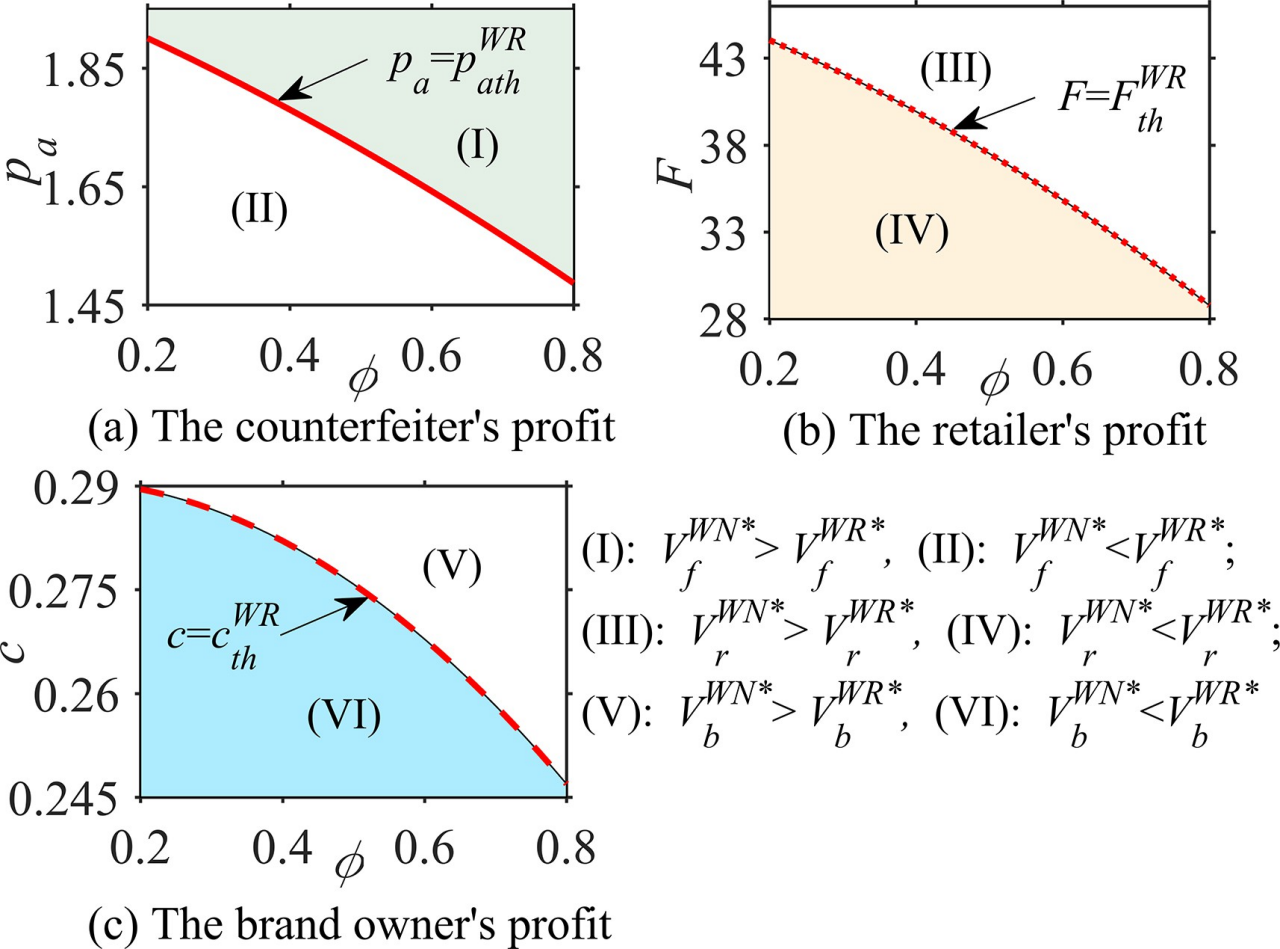
Impact of *ϕ* on the supervisory effect of blockchain in the *WR* case.

**Fig 14:**
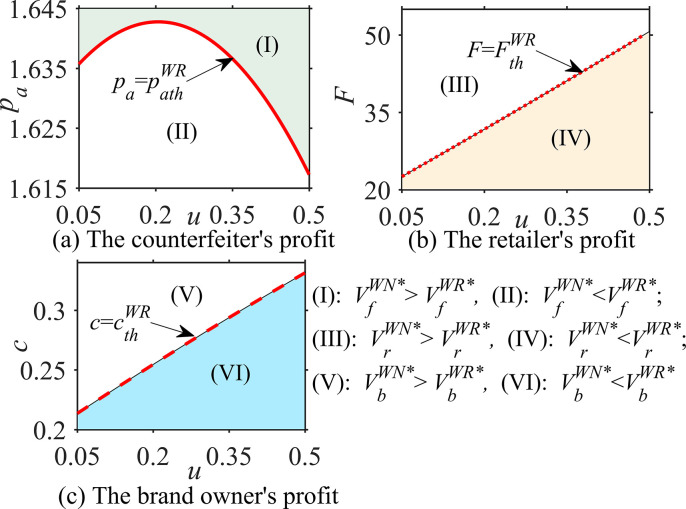
Impact of *u* on the supervisory effect of blockchain in the *WR* case.

From Proposition 13, it can be seen that in the wholesale sales mode, if and only if *p*_*a*_>*p*_*ath*_^*WR*^, *c*≤*c*_*th*_^*WR*^ and *F*≤*F*_*th*_^*WR*^ are there best conditions under which the retailer can establish blockchain to combat counterfeit products in the supply chain. Figs 12–14 describe the impacts of key parameters *l*, *ϕ* and *u*, respectively. In Fig 12, *p*_*ath*_^*WR*^ is negatively correlated with parameter *l*, while *c*_*th*_^*WR*^ and *F*_*th*_^*WR*^ are positively correlated with parameter *l*. This relationship shows that before the implementation of blockchain, the greater the influence degree of counterfeit products on brand goodwill, the more conducive it is for the retailer to establish blockchain to inhibit the counterfeiter, and the more conducive it is to improving the profits for both the retailer and the brand owner. This is different from the brand when establishing blockchain. At this time, for genuine enterprises, an increase in parameter *λ* is a win‒win‒win result. First, it enhances the possibility of the retailer opposing counterfeit products. Second, it increases the retailer’s motivation to establish blockchain, and third, it encourages the brand owner to participate in blockchain.

Fig 13 shows that *p*_*a*_>*p*_*ath*_^*WR*^, *c*≤*c*_*th*_^*WR*^ and *F*≤*F*_*th*_^*WR*^ are negatively correlated with parameter *ϕ*, which implies that before the implementation of blockchain, if consumers think that the product is genuine, the greater the probability *ϕ*, the more it will help the retailer to establish blockchain to combat the counterfeiter’s profitability, but the more detrimental it is in terms of improving the brand owner’s and retailer’s profits. This result is similar to the establishment of blockchain by the brand owner and will not be repeated to avoid redundancy. It can be seen from Fig 14 that with the increase in parameter *u*, *p*_*ath*_^*WR*^ is a concave function that increases first and then decreases, while *c*_*th*_^*WR*^ and *F*_*th*_^*WR*^ increase monotonically; this result indicates that the stronger the quality reference effect of consumers on genuine and counterfeit products after the implementation of blockchain, the more conducive it is for retailers to implement blockchain to improve their own and brand owners’ profits, and the more conducive it is in terms of reducing counterfeiters’ profits when parameter *u* is greater than a critical point. It can be seen that with the increase in parameter *u*, the retailer is more motivated to establish blockchain, and the brand owner is also more motivated to participate in blockchain; additionally, with the continuous increase in parameter *u*, the possibility of counterfeit products being opposed will also increase.

## 8 Conclusion

This paper constructs a supply chain system including a counterfeit product sales channel and a genuine product sales channel, in which the counterfeiter is in the counterfeit product sales channel, while the brand and retailer are in the genuine product sales channel. In this system, the choice of direct sales mode or wholesale sales mode by the brand owner is considered. At the same time, blockchain technology is considered to identify genuine and counterfeit products for consumers. Based on the state change of brand goodwill and the impact of blockchain technology on the quality information asymmetry of counterfeit products and genuine products and introducing the unit operation cost and fixed cost of blockchain, we constructed five cases of supply chain dynamic decision models for direct sales mode without blockchain (*DN* case) and the adoption of blockchain by the brand owner (*DB* case), and under the wholesale sales mode without blockchain (*WN* case), the establishment of blockchain by the brand owner (*WB* case) and the establishment of blockchain by the retailer (*WR* case) respectively. Firstly, we use the optimal control theory to solve the quality of counterfeit products, the optimal quality and service level of genuine products, as well as the optimal dynamic trajectories of consumer demand, brand goodwill and the expected discounted profits of supply chain members. Then, we systematically analyze these models based on equilibrium solutions under different cases and reveal the effect of blockchain technology in supervising counterfeit products in the supply chain by genuine enterprises. Finally, we further explore the impacts of key parameters on supply chain performance and the supervisory effect of counterfeit products in supply chain under different cases through numerical simulation.

### 8.1 Key findings and managerial insights

This paper results in the following main research conclusions and corresponding management insights, which point by point answer the RQ1~RQ4 raised in the Introduction section.

Answer to RQ1: According to the study described in Sections 4 and 5, we constructed models for different scenarios and used the HJB equation and backward induction to make equilibrium decisions about genuine enterprises and counterfeiters before and after blockchain application. Accordingly, in Section 6.2, we compared and analyzed the equilibrium solutions for the brand owner, retailer and counterfeiter in different cases (related to Propositions 4–8). In terms of RQ1, we obtain the following point-by-point findings. (1) We find that in the direct and wholesale sales modes, the adoption of blockchain by genuine enterprises forces the counterfeiter to improve the quality of their products, while the optimal quality and service level of genuine products may be improved or reduced depending on the specific blockchain adoption and threshold conditions. (2) In both the *WB* and *WR* blockchain adoption cases, the quality of the counterfeit products is the same; however, the optimal quality of genuine products is higher in the *WR* case, and the optimal service level is higher in the *WB* case. (3) Regardless of whether blockchain is adopted, the quality and service level of genuine products under the direct sales mode are higher than those under the wholesale sales mode, but the quality of counterfeit products has nothing to do with the brand owner’s sales mode. The above findings can provide a reference for the transformation of the sales mode of genuine enterprises and the optimal decision adjustment before and after the adoption of blockchain.

Answer to RQ2: According to the study described in Section 6.1, we reveal the influence of relevant parameters in the direct sales mode and wholesale sales mode on the optimal decisions of supply chain members before and after the adoption of blockchain (related to Propositions 1–3). In addition, the numerical simulation in Section 7 provides new insights for genuine enterprises in terms of the parameter sensitivity of supply chain performance and the supervisory effect of supply chain counterfeit products. In terms of RQ2, we obtain the following point-by-point findings. (1) We find that when the degree of influence of counterfeit products on brand goodwill increases; the optimal quality and service level of genuine products decrease, while the quality of counterfeit products increases. (2) If consumers believe that the product is more likely to be counterfeited, the optimal quality of genuine products is lower, while the quality of counterfeit products is greater to a certain extent. (3) Under blockchain technology, if the quality reference effect of consumers is enhanced, the quality of both genuine and counterfeit products will increase, while the optimal service level of genuine products will remain the same. (4) An increase in the blockchain unit operation cost reduces the optimal quality or service level of genuine enterprises but does not affect the optimal decision of counterfeiters. (5) The simulation results show that the sensitivity of the profits for genuine enterprises to key parameters is different from that of the counterfeiter. For example, in the case of *DB* of blockchain adopted by the brand owner in the direct sales mode, the brand owner’s profit is positively correlated with the quality reference effect coefficient of consumers but negatively correlated with the blockchain unit operating cost. In contrast, the counterfeiter’s profit is negatively correlated with the quality reference effect coefficient of consumers but positively correlated with the unit operation cost of blockchain. Our study warns that the sales of counterfeit products damage the quality and service level of genuine products by affecting brand goodwill. Meanwhile, these findings have a certain reference value for decision optimization of genuine enterprises in response to changes in the market environment. Additionally, genuine enterprises should adjust the conditions for the optimal supervisory effect of counterfeit products according to the changes in the market environment to inhibit counterfeiters and improve their profits.

Answer to RQ3: According to the study described in Section 6.3, we reveal the optimal dynamic trajectory change rules of the supply chain operation strategy in different cases (related to Propositions 9 and 10). In terms of RQ3, we obtain the following point-by-point findings. (1) We find that the optimal dynamic trajectories of the operation strategy for the counterfeiter and genuine enterprises without blockchain depend on the operation time, the initial value of brand goodwill and the sales price of genuine products. (2) The implementation of blockchain technology affects the evolution law of the supply chain operation strategy. (3) The optimal dynamic trajectories of the genuine enterprise operation strategy are also related to the unit operation cost of blockchain. The relevant conclusions can provide a reference and basis for the long-term dynamic development trend of genuine enterprises’ operation strategies, such as sales, brand goodwill and profit, in different sales modes, especially in the era of blockchain technology.

Answer to RQ4: According to the study described in Section 6.4, we determine the conditions for using blockchain to achieve a supervisory effect over counterfeit products in the direct sales mode and wholesale sales mode (related to Propositions 11 and 13). In terms of RQ4, we obtain the following point-by-point findings. (1) We find that if both the blockchain unit operating cost and fixed cost are less than the corresponding thresholds, the brand owner can not only combat the counterfeiter but also increase its own profitability by adopting blockchain. (2) In the wholesale sales mode and if and only if the sales price of genuine products is large and the unit operating cost and fixed cost of blockchain are small, the retailer can not only combat the counterfeiter but also improve its own and the brand owner’s profits by using blockchain. These research results can provide some management insights for genuine enterprises in terms of using blockchain technology to inhibit counterfeit products in the supply chain under different sales modes.

The differences between the results in this paper and those from existing studies consist of three aspects: First, we obtain the conclusion regarding the dynamic optimization of product quality and service decisions under brand reputation, which is different from the previous conclusion, which only considered the decisions made by genuine enterprises and ignored the impact of counterfeit products (e.g., Ma et al. [8]). Our conclusion is that optimal decision-making by genuine enterprises is influenced by the sales of counterfeit products. Second, we conclude that the optimal dynamic trajectory change rules of the supply chain operation strategy under blockchain technology are different from the existing research conclusions that explored supply chain dynamic operation without considering the impact of blockchain (e.g., Nerlove and Arrow [7]; Guan et al. [54]; Song et al. [55]). We find that the adoption of blockchain may change these rules. Third, we conclude that the effect of blockchain technology in terms of dynamically supervising counterfeit products is different from the existing research conclusions on using blockchain technology to combat counterfeiting in a static system (e.g., Shen et al. [1], Pun et al. [4]). We find that when the system is dynamic, the adoption conditions for blockchain technology are time-dependent and constrained by the state equation of brand goodwill. The significance of the findings in this paper is to provide a certain theoretical basis for related enterprises (e.g., Walmart and LVMH) to use blockchain technology to combat the sales of counterfeit products and to provide a decision-making reference for the dynamic optimization of product pricing and service decisions under blockchain technology. Managers can optimize the blockchain operation strategy of enterprises according to the research results in this paper to enhance brand goodwill and profits.

### 8.2 Limitations and future studies

Despite the significance of this study, there are still some limitations. On the one hand, although a numerical analysis was conducted in this paper, there is still a lack of empirical data used to test the model results, which may make our study insufficiently transparent in terms of the robustness of the results and methodology. On the other hand, this study is limited by the fact that market demand is deterministic, but uncertain market demand is more practical, which makes it very challenging to introduce the stochastic differential game method for further research. Future research directions that could be generalized are as follows: First, this paper only considers brand owners that have chosen the direct sales mode or wholesale sales mode, and further studies can be conducted on the mode in which the brand owner has both a direct sales mode and a wholesale sales mode. Second, this paper only considers the impact of blockchain on product quality information and further considers the impact of blockchain on demand information. Third, we can further expand the research on the strategies of multiple brand owners to combat counterfeit products in the supply chain based on blockchain technology in a competitive environment.

## Supporting information

S1 Appendix (DOCX)

S1 File Dataset. (DOCX)

## References

[1] Shen B, Dong C, Minner S. Combating Copycats in the Supply Chain with Permissioned Blockchain Technology. Production and Operations Management. 2022;31: 138–154.

[2] Luo Y, Sun J, Wang SL. Emerging Economy Copycats: Capability, Environment, and Strategy. Academy of Management Perspectives. 2011;25: 37–56.

[3] Li B, Kumar S. Should You Kill or Embrace Your Competitor: Cloud Service and Competition Strategy. Production and Operations Management. 2018;27: 822–838.

[4] Pun H, Swaminathan JM, Hou P. Blockchain Adoption for Combating Deceptive Counterfeits. Production and Operations Management. 2021;30: 864–882.

[5] Shen B, Xu X, Yuan Q. Selling secondhand products through an online platform with blockchain. Transportation Research Part E: Logistics and Transportation Review. 2020;142: 102066. doi: 10.1016/j.tre.2020.102066 32905037 PMC7462633

[6] Niu B, Mu Z, Cao B, Gao J. Should multinational firms implement blockchain to provide quality verification? Transportation Research Part E: Logistics and Transportation Review. 2021;145: 102121.

[7] Nerlove M, Arrow KJ. Optimal Advertising Policy under Dynamic Conditions. Economica. 1962;29: 129–142.

[8] Ma D, Hu J, Wang W. Differential game of product–service supply chain considering consumers’ reference effect and supply chain members’ reciprocity altruism in the online-to-offline mode. Annals of Operations Research. 2021;304: 263–297.

[9] Ma D, Hu J. The optimal combination between blockchain and sales format in an internet platform-based closed-loop supply chain. International Journal of Production Economics. 2022;254: 108633.

[10] Danese P, Mocellin R, Romano P. Designing blockchain systems to prevent counterfeiting in wine supply chains: a multiple-case study. International Journal of Operations & Production Management. 2021;41: 1–33.

[11] Haq I, Muselemu O. Blockchain Technology in Pharmaceutical Industry to Prevent Counterfeit Drugs. International Journal of Computer Applications. 2018;180: 8–12.

[12] Gao SY, Lim WS, Tang CS. Entry of Copycats of Luxury Brands. Marketing Science. 2017;36: 272–89.

[13] Pun H, DeYong GD. Competing with Copycats When Customers Are Strategic. Manufacturing & Service Operations Management. 2017;19: 403–418.

[14] Ghamat S, Pun H, Critchley G, Hou P. Using intellectual property agreements in the presence of supplier and third-party copycatting. European Journal of Operational Research. 2020;291: 680–92.

[15] Bian J, Zhang G, Zhou G. The strategic impact of vertical integration on non-deceptive counterfeiting. International Journal of Production Economics. 2023;260: 108863.

[16] Islam I, Muhammad Nazrul Islam. Digital intervention to reduce counterfeit and falsified medicines: A systematic review and future research agenda. Journal of King Saud University—Computer and Information Sciences. 2022;34: 6699–6718.

[17] Ziavrou KS, Noguera S, Boumba VA. Trends in counterfeit drugs and pharmaceuticals before and during COVID-19 pandemic. Forensic Science International. 2022;338: 111382. doi: 10.1016/j.forsciint.2022.111382 35882074 PMC9277998

[18] Ryu G, Kim B, Park K. Anti-counterfeiting advertisements for luxury brands in the post pandemic era: Roles of message type, visual presentation mode, and self-construal. Journal of Retailing and Consumer Services. 2023;73: 103354.

[19] Pittiglio R. Counterfeiting and firm survival. Do international trade activities matter? International Business Review. 2023;5: 102145.

[20] Houck MM. Counterfeit goods. Encyclopedia of Forensic Sciences. 2023;1: 664–667.

[21] Zhou Y, Gao X, Luo S, Xiong Y, Ye N. Anti-counterfeiting in a retail platform: A game-theoretic approach. Transportation Research Part E: Logistics and Transportation Review. 2022;165: 102839.

[22] Qian Y. Brand Management and Strategies Against Counterfeits. Journal of Economics & Management Strategy. 2014;23: 317–343.

[23] Cho SH, Fang X, Tayur S. Combating Strategic Counterfeiters in Licit and Illicit Supply Chains. Manufacturing & Service Operations Management. 2015;17: 273–289.

[24] Gao Y. On the Use of Overt Anti-Counterfeiting Technologies. Marketing Science. 2018;37: 403–424.

[25] Kumar S, Barua Mukesh Kumar. Exploring the hyperledger blockchain technology disruption and barriers of blockchain adoption in petroleum supply chain. Resources Policy. 2023;81: 103366.

[26] Kumar M, Choubey Vikas Kumar, Raut RD, Sandeep Jagtap. Enablers to achieve zero hunger through IoT and blockchain technology and transform the green food supply chain systems. Journal of Cleaner Production. 2023;405: 136894.

[27] Sunmola F, Burgess P. Transparency by Design for Blockchain-Based Supply Chains. Procedia Computer Science. 2023;217: 1256–1265.

[28] Di Vaio A, Varriale L. Blockchain technology in supply chain management for sustainable performance: Evidence from the airport industry. International Journal of Information Management. 2020;52: 102014.

[29] Xu M, Chen X, Kou G. A systematic review of blockchain. Financial Innovation. 2019;5: 1–14.

[30] Bhushan B, Sinha P, Sagayam KM, J A. Untangling blockchain technology: A survey on state of the art, security threats, privacy services, applications and future research directions. Computers & Electrical Engineering. 2020;90: 106897.

[31] Nazam M, Hashim M, Nută FM, Yao L, Zia MA, Malik MY, et al. Devising a Mechanism for Analyzing the Barriers of Blockchain Adoption in the Textile Supply Chain: A Sustainable Business Perspective. Sustainability. 2022;14: 16159.

[32] Tan TL, Salam I, Singh M. Blockchain-based healthcare management system with two-side verifiability. PLOS ONE. 2022;17: e0266916. 35421184 10.1371/journal.pone.0266916 PMC9009638

[33] Saeed H, Malik H, Bashir U, Ahmad A, Riaz S, Ilyas M, et al. Blockchain technology in healthcare: A systematic review. Vijayakumar P, editor. PLOS ONE. 2022;17: e0266462. doi: 10.1371/journal.pone.0266462 35404955 PMC9000089

[34] Choi TM. Blockchain-technology-supported platforms for diamond authentication and certification in luxury supply chains. Transportation Research Part E: Logistics and Transportation Review. 2019;128: 17–29.

[35] Sun Z, Xu Q, Shi B. Price and Product Quality Decisions for a Two-Echelon Supply Chain in the Blockchain Era. Asia-Pacific Journal of Operational Research. 2022;39: 2140016.

[36] Liu S, Hua G, Kang Y, Edwin Cheng TC, Xu Y. What value does blockchain bring to the imported fresh food supply chain? Transportation Research Part E: Logistics and Transportation Review. 2022;165: 102859.

[37] Guo Q, Zhao P, Cheng S, Ahmed M. Two-period price competition of second-hand product platforms with or without blockchain under different supply and demand levels. Computers & Industrial Engineering. 2023;178: 109131.

[38] Xu X, He P, Zhou L, Cheng TCE. Coordination of a platform-based supply chain in the marketplace or reselling mode considering cross-channel effect and blockchain technology. European Journal of Operational Research. 2023;309: 170–187.

[39] Liu J, Zhao H, Chen J. Blockchain-driven win–win strategy of economy and environment in the asymmetric competitive supply chain. Computers & Industrial Engineering 2023;176: 108978.

[40] Zhong Y, Yang T, Yu H, Zhong S, Xie W. Impacts of blockchain technology with government subsidies on a dual-channel supply chain for tracing product information. Transportation Research Part E: Logistics and Transportation Review. 2023;171: 103032.

[41] Xu L, Luo Y, Pu X. Information acquisition from data-driven analytics: A perspective of blockchain service in a duopoly market. Computers & Industrial Engineering. 2023;176: 108994.

[42] Pu S, Lam JSL. A game theoretic approach of optimal adoption time of blockchain: A case of ship operators. Computers & Industrial Engineering. 2022;169: 108219.

[43] Zhang Q, Jiang X, Zheng Y. Blockchain adoption and gray markets in a global supply chain. Omega. 2023;115: 102785.

[44] Emel Y. The role of blockchain technology in the sustainability of supply chain management: Grey based dematel implementation. Cleaner Logistics and Supply Chain. 2023;8: 100113.

[45] Choi TM, Guo S, Liu N, Shi X. Optimal pricing in on-demand-service-platform-operations with hired agents and risk-sensitive customers in the blockchain era. European Journal of Operational Research. 2020;284: 1031–1042.

[46] Gong B, Zhang H, Gao Y, Liu Z. Blockchain adoption and channel selection strategies in a competitive remanufacturing supply chain. Computers & Industrial Engineering. 2023;175: 108829.

[47] Wafaa A.H. Ahmed, MacCarthy. Blockchain-enabled supply chain traceability–How wide? How deep? International Journal of Production Economics. 2023;263: 108963.

[48] Li Y, Tan C, Ip WH, Wu CH. Dynamic blockchain adoption for freshness-keeping in the fresh agricultural product supply chain. Expert Systems with Applications. 2023;217: 119494.

[49] Li X. Inventory management and information sharing based on blockchain technology. Computers & Industrial Engineering. 2023;179: 109196.

[50] Meier O, Gruchmann T, Ivanov D. Circular supply chain management with blockchain technology: A dynamic capabilities view. Transportation Research Part E: Logistics and Transportation Review. 2023;176: 103177.

[51] Wang H, Wang C, Li M, Xie Y. Blockchain technology investment strategy for shipping companies under competition. Ocean & Coastal Management. 2023;243: 106696.

[52] Krishnamoorthy A, Prasad A, Sethi SP. Optimal pricing and advertising in a durable-good duopoly. European Journal of Operational Research. 2010;200: 486–497.

[53] Ma D, Qin H, Hu J. Achieving triple sustainability in closed-loop supply chain: The optimal combination of online platform sales format and blockchain-enabled recycling. Computers & Industrial Engineering. 2022;174: 108763.

[54] Guan Z, Ye T, Yin R. Channel coordination under Nash bargaining fairness concerns in differential games of goodwill accumulation. European Journal of Operational Research. 2020;285: 916–930.

[55] Song J, Chutani A, Dolgui A, Liang L. Dynamic innovation and pricing decisions in a supply-Chain. Omega. 2021;103: 102423.

[56] Liu Z, Huang YQ, Shang WL, Zhao YJ, Yang ZL, Zhao Z. Precooling energy and carbon emission reduction technology investment model in a fresh food cold chain based on a differential game. Applied Energy. 2022;326: 119945.

[57] Zhang Z, Yu L. Altruistic mode selection and coordination in a low-carbon closed-loop supply chain under the government’s compound subsidy: A differential game analysis. Journal of Cleaner Production. 2022;366: 132863.

[58] Wang W, Hao S, He W, Mohamed Abdulkadir Mohamed. Carbon emission reduction decisions in construction supply chain based on differential game with government subsidies. Building and Environment. 2022;222: 109149.

[59] Meng L, Wang J, Yan W, Han C. A differential game model for emission reduction decisions between ports and shipping enterprises considering environmental regulations. Ocean & Coastal Management. 2022;225: 106221.

[60] Tao W, Zhang C, Lu Y. Bilinear differential game for competitive advertising with stochastic disturbance and abrupt impact. Expert Systems with Applications. 2023;229: 120446.

[61] Wang Y, Yan Z. Pareto-based Stackelberg differential game for stochastic systems with multi-followers. Applied Mathematics and Computation. 2023;436: 127512.

[62] Feng J, Xie J. Research Note—Performance-Based Advertising: Advertising as Signals of Product Quality. Information Systems Research. 2012;23: 1030–1041.

[63] Li G, Li L, Sun J. Pricing and service effort strategy in a dual-channel supply chain with showrooming effect. Transportation Research Part E: Logistics and Transportation Review. 2019;126: 32–48.

[64] Tsay AA, Agrawal N. Channel Dynamics Under Price and Service Competition. Manufacturing & Service Operations Management. 2000;2: 372–391.

[65] Chiang WK, Chhajed D, Hess JD. Direct Marketing, Indirect Profits: A Strategic Analysis of Dual-Channel Supply-Chain Design. Management Science. 2003;49: 1–20.

[66] Taylor TA. Supply Chain Coordination Under Channel Rebates with Sales Effort Effects. Management Science. 2002;48: 992–1007.

