# Supplementary material for: Dynamic supervision of counterfeit products based on blockchain technology: A differential game on goodwill accumulation
Source: PLoS One. 2023 Oct 23;18(10):e0293346. doi: 10.1371/journal.pone.0293346 (PMC10593246; doi:10.1371/journal.pone.0293346)
Supplement: S1 Appendix — (DOCX) [file pone.0293346.s001.docx]

**Appendix**

**Proof of Theorem 1**

First, let *V_f_ ^DN^*(*G^DN^*) be the optimal value function of the counterfeiter, representing the total profit of the counterfeiter during the operation period. *V_f_ ^DN’^* (*G^DN^*) is the first derivative of the optimal value function with respect to *G^DN^*, representing the marginal contribution of brand goodwill to the profit of the counterfeiter. According to the optimal control theory, the optimal value function satisfies the following Hamilton-Jacobi-Bellman (HJB) equation:

|  |  |
| --- | --- |

According to the first-order optimality condition on *q_f_ ^DN^*(*t*) at the right end of HJB equation, we can get:

|  |  |
| --- | --- |

Substituting Eq. (A2) into HJB Eq. (A1) can obtain:

|  |  |
| --- | --- |

According to the structure of HJB Eq. (A3), let *V_f_ ^DN^*(*G^DN^*)=*E_f_ ^DN^*⋅*G^DN^* +*L_f_ ^DN^*, where *E_f_ ^DN^* and *L_f_ ^DN^* are undetermined constant coefficients. In order to obtain the undetermined constant coefficient, the optimal value function *V_f_ ^DN^*(*G^DN^*) and its derivative *V_f_ ^DN’^* (*G^DN^*)= *E_f_ ^DN^* are substituted into the HJB Eq. (A3), and according to the identity relationship, the parameter expressions of *E_f_ ^DN^* and *L_f_ ^DN^* can be obtained. If *E_f_ ^DN^* is substituted into Eq. (A2), Eq. (10) in Theorem 1 can be obtained.

Next, the optimal decisions of the brand owner are solved. Let *V_b_ ^DN^*(*G^DN^*) be the optimal value function of the brand, and *V_b_^DN’^* (*G^DN^*) be the first derivative of the optimal value function with respect to *G^DN^*. According to the optimal control theory, the HJB equation of the brand owner can be obtained as follows:

|  |  |
| --- | --- |

By solving the HJB equation of the brand owner according to the first-order optimality condition, we can get:

|  |  |
| --- | --- |
|  |  |

Substituting Eqs. (8), (A5) and (A6) into Eq. (A4), the HJB equation of the brand owner can be obtained as

|  |  |
| --- | --- |

Let the optimal value function of the brand owner *V_b_ ^DN^*(*G^DN^*)= *E_b_ ^DN^*⋅*G^DN^* +*L_b_^DN^*, where *E_b_^DN^* and *L_b_ ^DN^* are undetermined constant coefficients. Furthermore, the optimal value function *V_b_^DN^*(*G^DN^*) and its derivative *V_b_^DN’^* (*G^DN^*)= *E_b_^DN^* are substituted into the HJB Eq. (A7), and the *E_b_ ^DN^* and *L_b_ ^DN^* can be obtained according to the identity relationship. The Eqs. (8) and (9) in Theorem 1 can be obtained by substituting *E_b_ ^DN^* into equations (A5) and (A6). □

**Proof of Corollary 1**

According to *q_f_ ^DN*^*(*t*), *q_a_^DN*^*(*t*) and *s_a_^DN*^*(*t*) in Theorem 1, it is substituted into the state equation *G^DN^*(*t*) of brand goodwill, and *G^DN*^*(*t*) can be solved according to the first-order linear differential equation. Furthermore, the optimal decisions and *G^DN*^*(*t*) are contemporaneously incorporated into Eq. (6), Eq. (7), and *V_f_ ^DN^*(*G^DN^*)=*E_f_ ^DN^*⋅*G^DN^* +*L_f_ ^DN^* and *V_b_ ^DN^*(*G^DN^*)=*E_b_ ^DN^*⋅*G^DN^* +*L_b_ ^DN^*, and the Eqs. (12)~(15) in Corollary 1 can be obtained in turn. □

**Proofs of Theorems 2~5**

According to the optimal control theory and the backstepping induction method of dynamic game, the optimal decisions of the counterfeiter and genuine enterprises in different cases can be solved. Similar to the proof of equilibrium outcomes in Theorem 1 and thus omitted. □

**Proofs of Propositions 1~3**

According to the optimal solutions in Theorems 1-5, the quality of the counterfeit products and the optimal quality and service level of the genuine products in different case are subjected to first-order derivation of each basic parameter and judged to be positive or negative to obtain Propositions 1 to 3. □

**Proof of Proposition 4**

Proposition 4 can be obtained by subtracting the optimal decisions under *DN* case from the optimal decisions under *DB* case and judging the positive and negative. □

**Proof of Proposition 5**

Proposition 5 is obtained by subtracting the optimal decisions in the *WN* case from the optimal decisions in the *WB* and *WR* cases, respectively, and determining the positive and negative. □

**Proof of Proposition 6**

By comparing the equilibrium outcomes under *WR* case and *WB* case, we can derive the results in Proposition 6. □

**Proof of Proposition 7**

By comparing the equilibrium outcomes under *DN* case and *WN* case, we can derive the results in Proposition 7. □

**Proof of Proposition 8**

Proposition 8 is obtained by subtracting the optimal decisions in the *DB* case from the optimal decisions in the *WB* and *WR* cases, respectively, and determining the positive and negative. □

**Proof of Proposition 9**

According to the brand goodwill *G^DN^*^*^(*t*), consumers' demand for counterfeit products *d_f_^DN^*^*^(*t*), consumers' demand for authentic products *d_a_^DN^*^*^(*t*), the counterfeiter’s expected discounted profit *V_f_^DN^*^*^(*t*) and brand owner's expected discounted profit *V_a_^DN^*^*^(*t*) in the case of *DN* in Corollary 1, when *t*→+∞, e*^-σ⋅t^*→0, we can obtain *G^DN^*^*^(*t*)=*G*_∞_*^DN^*^*^=((*α_a_⋅q_a_^DN^*^*^ +*δ_a_⋅s_a_^DN^*^*^)(1-*λ*)+*λα_a_⋅q_f_^DN^*^*^)/*σ*, *d_f_^DN^*^*^(*t*)= *d_f_^DN^*^*^(*G*_∞_*^DN^*^*^), *d_a_^DN^*^*^(*t*)= *d_a_^DN^*^*^(*G*_∞_*^DN^*^*^), *V_f_^DN^*^*^(*t*)= *d_f_^DN^*^*^(*G*_∞_*^DN^*^*^) and *V_a_^DN^*^*^(*t*)= *d_f_^DN^*^*^(*G*_∞_*^DN^*^*^). Therefore, there is *t*=*t_th_^DN^*(*t_th_^DN^* →+∞). When *t* ≥ *t*_th_*^DN^*, ∂*G^DN^*^*^(*t*)/ ∂*t*=0, ∂*d_f_ ^DN^*^*^(*t*)/ ∂*t*=0, ∂*d_a_ ^DN^*^*^(*t*)/ ∂*t*=0, ∂*V_f_ ^DN^*^*^(*t*)/ ∂*t*=0 and ∂*V_a_ ^DN^*^*^(*t*)/ ∂*t*=0; When *t*< *t_th_^DN^*, *G^DN^*^*^(*t*), *d_f_^DN^*^*^(*t*), *d_a_^DN^*^*^(*t*), *V_f_^DN^*^*^(*t*) and *V_a_^DN^*^*^(*t*) are respectively calculated for the first derivative of time *t* and judged positive and negative, then the result in the case of *DN* in Proposition 9(*i*) can be obtained. Similarly, we can get the result of *WN* case in Proposition 9. □

**Proof of Proposition 10**

Referring to the proof of Proposition 9, we can similarly determine the optimal dynamic trajectories of the relevant variables in the *DB*, *WB* and *WR* cases, respectively. □

**Proof of Proposition 11**

According to Corollaries 1 and 2, the *V_f_ ^DB*^* and *V_b_^DB*^* in steady state (i.e., *t*→+∞) are subtracted from the corresponding *V_f_ ^DN*^* and *V_b_ ^DN*^* and judged positive and negative to obtain Proposition 11. □

**Proof of Proposition 12**

According to Corollaries 2 and 3, the *V_f_ ^WB*^*, *V_b_^WB*^* and *V_r_^WB*^* in steady state (i.e., *t*→+∞) are subtracted from the corresponding *V_f_ ^WN*^*, *V_b_ ^WN*^* and *V_r_^WN*^* and judged positive and negative to obtain Proposition 12. □

**Proof of Proposition 13**

According to Corollaries 2 and 3, the *V_f_ ^WR*^*, *V_b_^WR*^* and *V_r_^WR*^* in steady state (i.e., *t*→+∞) are subtracted from the corresponding *V_f_ ^WN*^*, *V_b_ ^WN*^* and *V_r_^WN*^* and judged positive and negative to obtain Proposition 13. □
