# Supplementary material for: Dynamic supervision of counterfeit products based on blockchain technology: A differential game on goodwill accumulation
Source: PLoS One. 2023 Oct 23;18(10):e0293346. doi: 10.1371/journal.pone.0293346 (PMC10593246; doi:10.1371/journal.pone.0293346)
Supplement: S1 File — (DOCX) [file pone.0293346.s002.docx]

Dataset

We construct several differential game models before and after blockchain adoption in a supply chain consisting of genuine enterprises and counterfeiter, respectively, analyze the effect of blockchain technology in supervising counterfeit products, examine the optimal dynamic trajectories of supply chain operation strategy, and present some useful results. Readers can replicate the results of our study through the proofs in the S1 Appendix.

In numerical analysis of this paper, the original data of parameters are: *a*=0.4; *p_f_*=0.28; *p_a_*=0.3; *w*=0.2; *ρ*=0.1; *F*=0.1; *G*_0_=0.1; *α_f_* =0.7; *α_a_* =0.8; *δ_f_* =0.4; *δ_a_*=0.5; *σ*=0.05; *λ*=0.3; *θ*=0.1; *k_a_*=0.01; *η_f_*=0.02; *η_a_* =0.01; *β*=0.6; *γ*=0.4; *φ*=0.6; *u*=0.25; *c*=0.03. Substituting these data to equations we obtained in the paper, the readers can redraw the figures in this paper.
